# Comparative efficacy and safety of immunomodulatory therapies for sepsis: a systematic review and network meta-analysis

**DOI:** 10.3389/fmed.2026.1808427

**Published:** 2026-05-12

**Authors:** Rong Luo, Shunyao Xu, Yuting Chen, Zhenmi Liu, Xiaofan Deng, Chenxi Li, Kangping Hui, Youlian Chen, Chengying Hong, ChunBo Chen, Huaisheng Chen

**Affiliations:** 1 Department of Critical Care Medicine, Shenzhen People’s Hospital, The Second Clinical Medical College of Jinan University, Shenzhen, Guangdong, China; 2 Department of Critical Care Medicine, Shenzhen People’s Hospital, The First Affiliated Hospital, Southern University of Science and Technology, The Second Clinical Medical College of Jinan University, Shenzhen, China; 3 Department of Geriatrics, Guangdong Provincial Clinical Research Center for Geriatrics, Shenzhen Clinical Research Center for Geriatrics, Shenzhen People’s Hospital, The First Affiliated Hospital, Southern University of Science and Technology, The Second Clinical Medical College of Jinan University, Shenzhen, Guangdong, China

**Keywords:** immunomodulator, immunotherapy, mortality, network meta-analysis, sepsis

## Abstract

**Background:**

As a standard therapy, immunotherapy is widely used for sepsis patients. Despite the presence of various immunomodulators, studies comparing their safety and efficacy synthetically are still lacking.

**Methods:**

Electronic databases (PubMed, Embase, and the Cochrane Library) were searched from inception to March 31, 2025. The primary endpoint assessed was all-cause mortality, whereas secondary outcomes included duration of mechanical ventilation (MV duration), length of intensive care unit (ICU-LOS) and hospital stay (hospital-LOS). Safety was evaluated by monitoring adverse events or serious adverse events (AEs/SAEs). For effect estimation, the risk ratio (RR) and mean difference (MD) with a 95% confidence interval (95% CI) were utilized. The network meta-analysis was executed via the ‘BUGSnet’ and ‘JAGS’ packages within R 4.4.2. Interventions were ranked by surface under the cumulative ranking curve (SUCRA) values. The risk of bias was assessed with the Cochrane RoB tool, and evidence quality was graded via GRADE.

**Results:**

A total of 76 randomized controlled trials (RCTs) involving 22,194 patients were included. Ulinastatin had the most favorable effect on reducing all-cause mortality [RR 0.37 (0.22, 0.59)]. Ulinastatin plus thymosin-α1, polyunsaturated fatty acids (PUFA), and monoclonal antibody (MAb) also lowered mortality versus other treatments [RRs 0.65 (0.54, 0.77), 0.74 (0.61, 0.91), 0.92 (0.84, 0.99)]. Ulinastatin plus thymosin-α1 reduced ICU-LOS [MD −2.91 (−5.39, −0.44)], while PUFA shortened hospital-LOS [MD −20.55 (−39.81, −0.51)]. Both ulinastatin alone and with thymosin-α1 shortened MV duration [MDs − 4.43 (−8.32, −0.49) and −1.86 (−3.14, −0.41)]. Ulinastatin (with/without thymosin-α1) and PUFA were linked to fewer SAEs.

**Conclusion:**

On the basis of the findings of this first network meta-analysis evaluating a broad spectrum of immunomodulators for sepsis, ulinastatin (alone or in combination with thymosin-α1), PUFA, and MAb have significant potential for reducing mortality and improving other clinical outcomes. However, the certainty of evidence for most comparisons remains low, which underscores the need for large-scale, direct-comparison RCTs to validate these findings and guide clinical practice.

## Background

Sepsis arises from a systemic infection characterized by a dysregulated immune response, which is further exacerbated by organ failure, rendering it a life-threatening condition (1, 2). Despite improvements in treatment modalities, the incidence of morbidity and mortality associated with sepsis remains significantly elevated (3). The pathophysiology of the disease progresses from an initial hyper-inflammatory phase to a later stage marked by immune suppression (4–6). It is noteworthy that an exaggerated immune inflammatory response may coincide with cellular immune suppression.

The innate inflammatory mechanism in sepsis is dominated by complement and coagulation systems, with cytokines secreted by leukocytes, endothelial and immune cells (7). Excessive expression of inflammatory mediators such as tumor necrosis factor (TNF) and interleukin-1 (IL-1) is a key contributor to tissue damage (8). The complement system is activated with the generation of C3a and C5a (9), which recruit immune cells and platelets (10), increase vascular permeability (11), and promote leukocyte migration (12). The body subsequently mounts an anti-inflammatory response (13) that, when excessive or prolonged, causes severe immune suppression (14). This state features elevated anti-inflammatory cytokines, lymphocyte exhaustion and apoptosis, impaired antigen presentation, increased PD-1 expression, and reduced HLA-DR levels. All contribute to the immunological characteristics of sepsis (15, 16).

With the deepening of our understanding of sepsis, immune modulation is considered to potentially improve the prognosis of patients (17). On the basis of the immunological characteristics of sepsis, immune modulation focuses on anti-inflammatory effects in the early stage and aims mainly to enhance cellular or humoral immune function in the late stage (18). Glucocorticoids and ulinastatin may effectively inhibit excessive inflammatory responses (19, 20). Immunoglobulin IgG was once thought to enhance the body’s humoral immune function and improve the prognosis of patients. However, many clinical studies have yielded opposite results (21). In contrast, monoclonal IgM may have certain positive effects (22). On the other hand, interventions to improve cellular immune function seem to achieve better clinical outcomes (23). These interventions include thymosin (24), granulocyte/macrophage-colony stimulating factor (25), PD-1-related therapeutic interventions (26), IL-7 (27), some monoclonal antibodies (28, 29), and n-3 polyunsaturated fatty acids (30). The immune modulation measures and their mechanisms of action are shown in Table 1.

**Table 1:** Main immunomodulators in sepsis.

Immune-modulator agent	Mechanism of action
Steroids	Suppress pro-inflammatory gene transcription via glucocorticoid receptor binding and NF-κB inhibition; reduce cytokine production (TNF-α, IL-1β, IL-6); stabilize endothelial barriers; improve hemodynamics
Ulinastatin (UTI)	Inhibits neutrophil elastase and other proteases; stabilizes lysosomal membranes; reduces NF-κB activation and subsequent TNF-α and IL-1β production; protects endothelial glycocalyx; attenuates tissue damage
Immunoglobulin (PIg)	Neutralizes bacterial endotoxins and exotoxins; modulates complement activation; enhances opsonization; Fc receptor-mediated immune modulation; restores serum immunoglobulin levels in deficiency states
Thymosin-α1 (Tα1)	Enhances T-cell differentiation and maturation; promotes Th1 cytokine response (IFN-γ, IL-2); reduces T-cell apoptosis; improves antigen presentation via dendritic cell activation; restores cell-mediated immunity
G/M-CSF	Stimulates hematopoietic stem/progenitor cells (HSPCs) in bone marrow; promotes neutrophil (G-CSF) and monocyte/macrophage (GM-CSF) generation and differentiation; enhances phagocytic activity and microbial killing
PD-1/PD-L1	Blocks PD-1/PD-L1 interaction; reverses T-cell exhaustion and apoptosis; restores T-cell proliferation and cytokine secretion (IFN-γ); enhances anti-pathogen immune responses; improves adaptive immunity
IL-7	Promotes T-cell survival, proliferation, and homeostatic expansion; enhances thymic output; reverses lymphopenia; improves T-cell trafficking and functionality; restores adaptive immune responses
Monoclonal antibodies (MAb)	Targets specific immune molecules or pathogens; includes anti-endotoxin (e.g., anti-LPS), anti-cytokine (e.g., anti-TNF), and immune checkpoint inhibitors (e.g., anti-PD-1); neutralizes harmful mediators or blocks inhibitory signals
N-3 polyunsaturated fatty acids (PUFA)	Incorporates into cell membranes; modulates lipid raft composition; reduces NF-κB activation; decreases pro-inflammatory cytokine production (TNF-α, IL-1β, IL-6); promotes pro-resolving mediators (resolvins, protectins); improves T-lymphocyte function

NF-κB, nuclear factor-kappa B; TNF-α, tumor necrosis factor-alpha; IL-1β, interleukin-1 beta; IL-6, interleukin-6; IL-2, interleukin-2; IFN-γ, interferon-gamma; HSPCs, hematopoietic stem/progenitor cells; G-CSF, granulocyte colony-stimulating factor; GM-CSF, granulocyte-macrophage colony-stimulating factor; PD-1, programmed death-1; PD-L1, programmed death-ligand 1; IL-7, interleukin-7; LPS, lipopolysaccharide; Th1, T helper type 1.

Since these interventions that affect immune mechanisms currently exist only in clinical research, they have not yet been accepted by the guidelines and most of the clinical research results also vary. Therefore, we intend to conduct this study to perform a meta-analysis of previously published clinical trials, evaluate the effects of existing immune modulation measures on the mortality rate of patients with sepsis, and compare the differences in the effects of different interventions.

## Methods

This systematic review follows the Preferred Reporting Items for Systematic Reviews and Meta-Analyses for Network Meta-Analyses (PRISMA-NMA) checklist (31). Ethics approval was not considered necessary as all analyses used previously published data.

### Data sources and search strategy

A comprehensive search was conducted across three databases (PubMed, EMBASE, and the Cochrane Central Register of Controlled Trials) using database specific search methods without any language limitation from the beginning until March 2025 to identify trials that may be eligible for inclusion. The search strategy is presented in Appendix 1.

### Study selection and data extraction

Each record was independently reviewed by two reviewers, first by title and abstract and then by full-text. Data from each included primary study were independently extracted by two reviewers using a pre-specified online form. Disagreements between reviewers were solved by consensus or by a third reviewer.

### Eligibility criteria

The eligibility criteria were based on the “Population-Intervention-Control-Outcomes-Studies” (PICOS) approach (31).

#### Participants

Adult critically ill patients with sepsis who received immunomodulatory drugs were enrolled in this network meta-analysis. The inclusion and exclusion criteria of each study are provided in Appendix 3.

#### Interventions

Interventions were limited to clinically relevant immunomodulators that are commonly used or widely investigated in sepsis trials, irrespective of dose, duration, or co-intervention. The following agents were included corticosteroids, ulinastatin (UTI), polyclonal immunoglobulin (PIg), thymosin-α1 (Tα1), G/GM-CSF, PD-1, IL-7, MAb, and PUFA. Agents such as statins, IL-1 inhibitors or other immunomodulators not listed were excluded due to insufficient direct comparative evidence or limited clinical use in sepsis. Additionally, monoclonal antibodies included, but not limited to, anti-tumor necrosis factor (anti-TNF), anti-endotoxin, and anti-PD-1 antibodies.

#### Comparisons

The comparators included patients receiving no intervention, placebo or standard of care (SOC). SOC was defined as guideline-recommended management according to the Surviving Sepsis Campaign guidelines applicable at the time of each trial. Standard care included but was not limited to early antimicrobial therapy, fluid resuscitation, vasopressor support, and organ support as clinically indicated.

#### Outcomes

The primary endpoint of this network meta-analysis was all-cause death. The secondary endpoints included the intensive care unit (ICU) and overall hospital length of stay (LOS), duration of mechanical ventilation, and incidence of adverse events (AEs) and serious adverse events (SAEs). These factors are major indicators of treatment efficacy and safety. All outcomes were analyzed as originally defined in component studies for consistency, to be inclusive and because of the possibility of clinical heterogeneity in definition across research environments. Specifically, SAEs were defined as events requiring specific therapeutic intervention (e.g., vasopressor support, renal replacement therapy, or mechanical ventilation), prolonging ICU or hospital length of stay, or leading to life-threatening consequences or fatal outcomes (e.g., septic shock, multiple organ dysfunction syndrome, or death) in this analysis.

#### Types of studies

RCTs were incorporated into the analysis without regard to factors such as blinding, publication status, or language. Conversely, quasi-randomized trials and historically controlled clinical trials were excluded from consideration.

### Risk of bias and quality of evidence

The risk of bias was evaluated via the Cochrane Collaboration tool (32). Methodological quality bias was assessed on the basis of the following domains: random sequence generation (selection bias), allocation concealment (selection bias), blinding of participants and personnel (implementation bias), blinding of outcome assessment (detection bias), incomplete outcome data (attrition bias), selective reporting, and other biases. The risk of bias in each domain for each study was judged to be low, unclear, or high. Studies at low risk of bias for all domains were considered to be at low risk of bias. Any study with a high risk of bias for one or more domain(s) was considered at high risk of bias. Two reviewers independently evaluated the performance and detection bias. Discrepancies were resolved through discussion.

We describe the confidence in the evidence by using the Grading of Recommendations, Assessment, Development, and Evaluation (GRADE) approach to describe the certainty in the evidence for each comparison as high, moderate, low, or very low (33, 34).

### Data extraction and synthesis

Data were extracted independently by two reviewers for each therapeutic (Ulinastatin: RL and ZL. Ulinastatin plus Thymosin a1: RL and ZL. PUFA: RL and KH. Immunoglobulin: RL and YLC. PD-1: RL and CXL. Steroid: YTC and SX. MAb: SX and RL. G/GM-CSF: CXL and CH) via a self-developed data extraction form. The subsequent characteristics and data were systematically extracted from each trial included in the analysis: bibliographic details (first author, countries involved, year of publication); study attributes (design and quality of the study, criteria for patient inclusion and exclusion, sample size, dosage and duration of interventions, primary and secondary outcomes, incidence and classification of adverse events, evaluation metrics); and baseline characteristics of the patient population (mean age, sex ratio). Data regarding the number of patients who achieved each outcome, categorized by assigned treatment group, regardless of compliance or follow-up, were sought to facilitate an intention-to-treat analysis.

All the statistical analyses were carried out via the “BUGSnet” and “JAGS” packages within the R statistical software (Version 4.4.2) (35). Risk ratios (RRs) with 95% confidence intervals (CIs) were computed for dichotomous variables, and mean differences (MDs) with 95% CIs were determined for continuous variables. If the data were presented as medians and interquartile ranges in the initial documents, we estimated the means and SDs via the online tool at https://www.math.hkbu.edu.hk/~tongt/papers/median2mean.html (36).

Assumptions of homogeneity, transitivity and consistency underpinned the validity of conclusions arising from NMA analyses (37). Both random-effects models and fixed-effects models were applied, and the more fitting model was chosen for subsequent analysis. Model fit was assessed via the deviance information criterion (DIC) (38). Lower values of the DIC are indicative of a better balance of fit and model complexity. We assessed the statistical heterogeneity among direct comparisons of included trials via a random-effects model with an inverse variance weighting method. The assumption of homogeneity was considered as being fulfilled if the degree of heterogeneity with direct pairwise comparisons was acceptable. The I^2^ statistic was used to assess heterogeneity. In particular, I^2^ values of 0–24.9%, 25–49.9%, 50–74.9%, and 75–100% indicated no, low, moderate, and high heterogeneity, respectively (39). The transitivity assumption was fulfilled when the studies included were sufficiently similar in terms of their methodological and clinical characteristics. Similarly, statistical consistency was also evaluated via DIC. A difference in DIC of more than 5 points was considered substantial evidence favoring the model with the lower DIC. If the consistency model demonstrated a lower or similar DIC (difference < 5), it was preferred for its parsimony. Additionally, the leverage diagram was used to select either the consistency model or the inconsistency model.

A network plot was constructed to elucidate the comparative relationships among various interventions. League plots were generated to depict the relative effects of each intervention pair. Surface under the cumulative ranking curve (SUCRA) values were employed to systematically rank each immunomodulator from highest to lowest. Elevated SUCRA values signify a more favorable treatment ranking.

## Results

### Study selection, characteristics and risk of bias assessment

**Figure 1:**
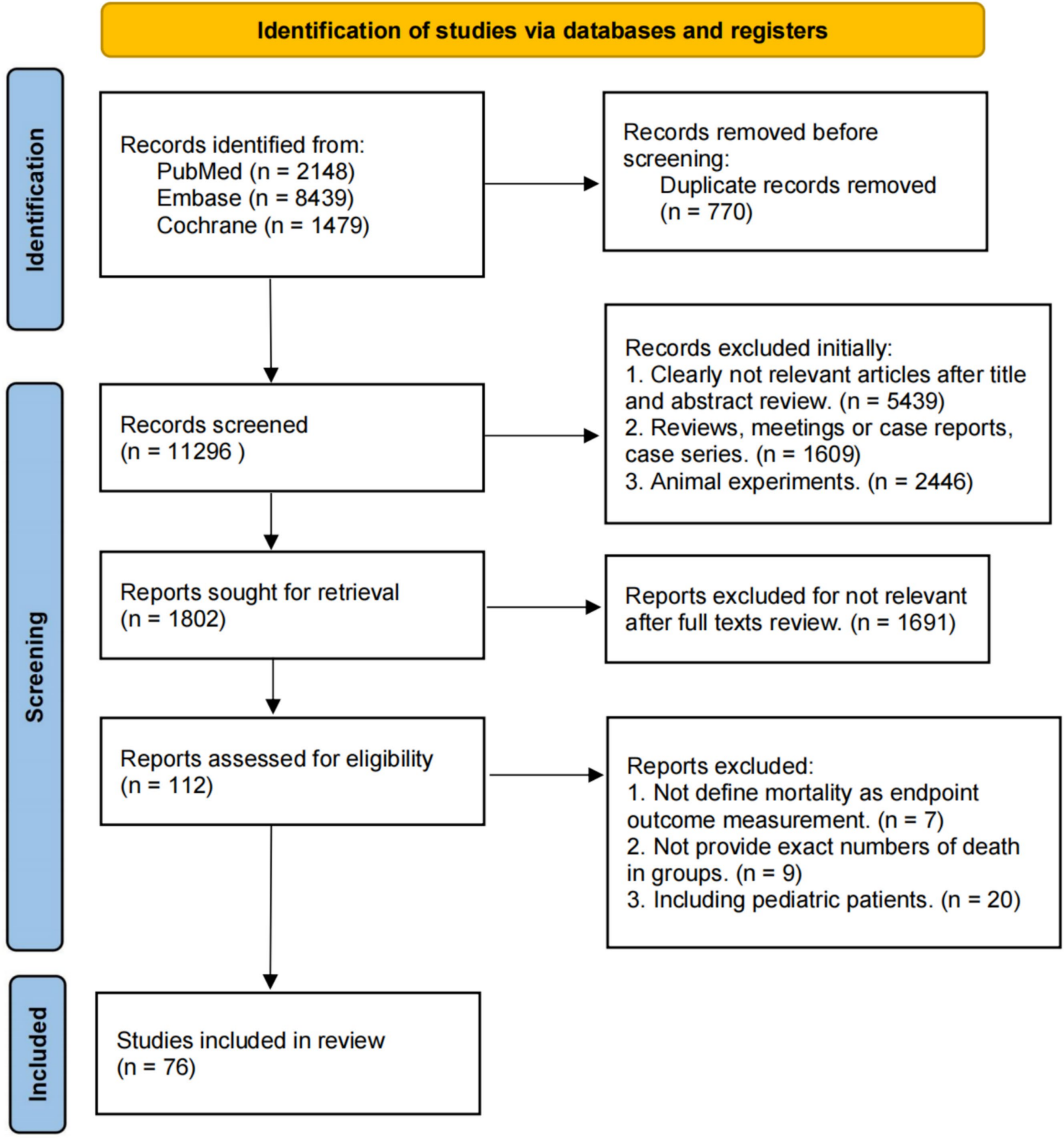
The PRISMA flow diagram of study selection.

A flow diagram of the study selection process is shown in Figure 1. The systematic search conducted in this study resulted in the identification of 11,296 records, followed by the removal of duplicate entries. Following a thorough screening of titles and abstracts, a total of 1,802 full-text articles were evaluated for potential inclusion. Ultimately, this meta-analysis included 76 eligible randomized controlled trials (RCTs), which collectively enrolled 22,194 patients (Appendix 2). The studies were published in English or Chinese and were conducted primarily in Asia (34.2%), Europe (31.6%) and North America (27.6%) between 1987 and 2025. Among the included studies, 43 were conducted in multicenter ICUs, and the other 33 RCTs were conducted in a single-center ICU. A total of 9 immunotherapies were investigated including monoclonal antibody (MAb, 18 RCTs), polyclonal immunoglobulin (PIg, 7 RCTs), n-3 polyunsaturated fatty acids (PUFA, 10 RCTs), corticosteroid (steroid, 18 RCTs), interleukin-7 (IL-7, 2 RCTs), granulocyte/macrophage-colony stimulating factor (G/M-CSF, 5 RCTs), ulinastatin (UTI, 5 RCTs), thymosin α1 (Tα1, 5 RCTs), and ulinastatin combined with thymosin α1 (UTI + Tα1, 6 RCTs). During the full-text screening and data extraction process, we identified that among all studies investigating PD-1 pathway modulation in sepsis, only one eligible RCT evaluated an anti-PD-1 antibody. To avoid creating an isolated node with a single study and to align with its pharmacological class, the data from this trial were merged into the MAb group for analysis. The mean age at randomization ranged from 36.7 to 72 years, and 41 (53.9%) trials reported a mean age of 60 years or older. The characteristics of the included studies, treatment plans of the trials and outcomes are shown in Appendix 4.

**Figure 2:**
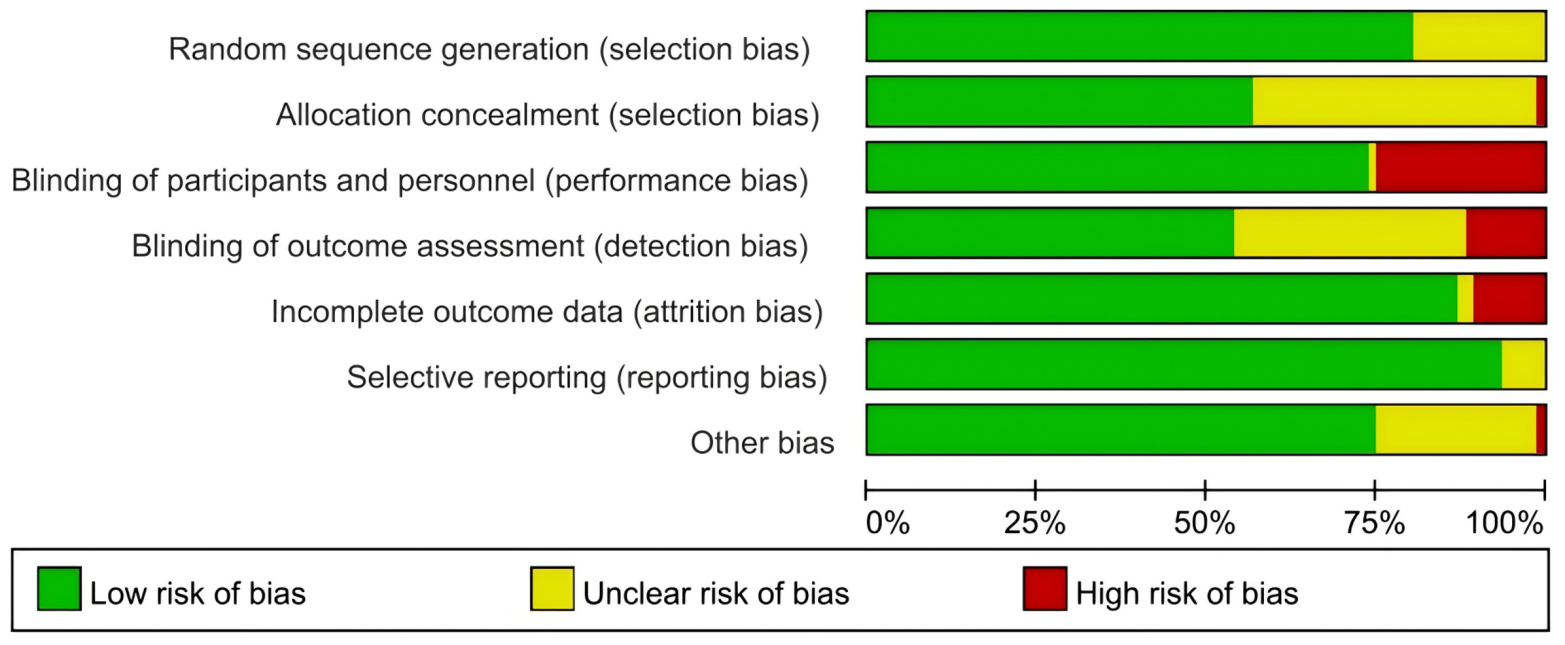
Summarized risk of the included randomized controlled trials.

As shown in Figure 2, 35.5% (27) of the trials were at high risk of bias, mostly owing to a lack of blinding of (participants, personnel and outcome assessment) and incomplete data on outcomes; the other 24 trials were at moderate risk and 25 trials were at low risk for bias. Notably, the lowest risk is related to selective reporting, with more than 85% of studies estimated to be at low risk for bias. More than two-thirds of the trials were judged as having a low risk of bias in random sequence generation and attrition. The details for the risk of bias of individual RCTs are presented in Appendix 5.

### Assessment of heterogeneity, transitivity and inconsistency

The findings of heterogeneity in the direct pairwise comparisons are shown in Appendix 6. In general, there was no significant heterogeneity in the majority of direct pairwise comparisons except for some pairs of direct comparisons in length of ICU stay and duration of hospital stay (Appendix 6). Publication bias was not formally assessed because the number of trials in each direct comparison was insufficient (k < 10) to support funnel plot interpretation. We assessed the inclusion and exclusion criteria used in the eligible studies, with a special focus on critically ill adult patients with sepsis (Appendix 3). We have not observed any significant violations to the transitivity in our network. We found that the enrolled patients were sufficiently clinically similar and identified the likelihood of major imbalances in effect modifiers across trials. The comparison between the consistency and inconsistency models yielded DIC values that were similar. When the difference was less than 5, there was no strong statistical evidence to favor one model over the other. Residual analysis under the consistency model revealed one standardized residual with an absolute value for the comparison, suggesting potential but inconclusive localized inconsistency. Given the parsimony and clinical interpretability of the consistency model, and the lack of strong evidence against it, we present the results from the consistency model as the primary analysis.

### All-cause mortality

**Figure 3:**
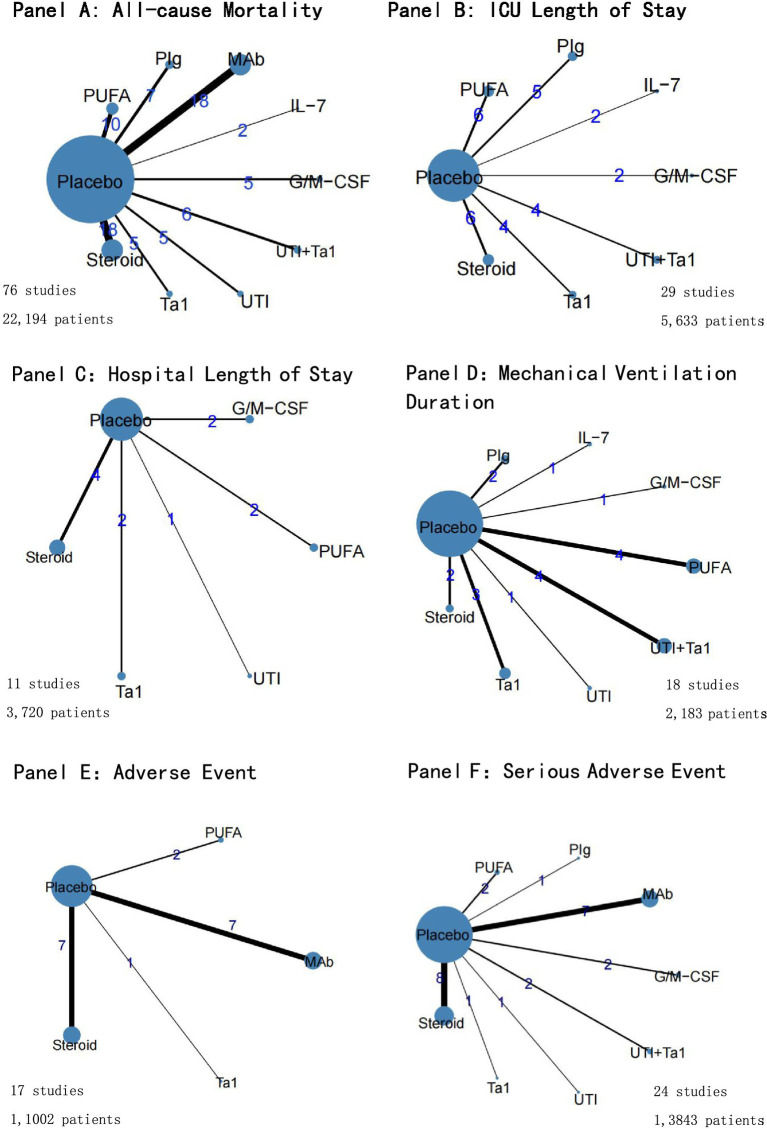
Network plots for outcomes of immunotherapy. Panel **(A)**: All-cause Mortality; Panel **(B)**: ICU Length of Stay; Panel **(C)**: Hospital Length of Stay; Panel **(D)**: Mechanical Ventilation Duration; Panel **(E)**: Adverse Events; Panel **(F)**: Serious Adverse Events.

**Figure 4:**
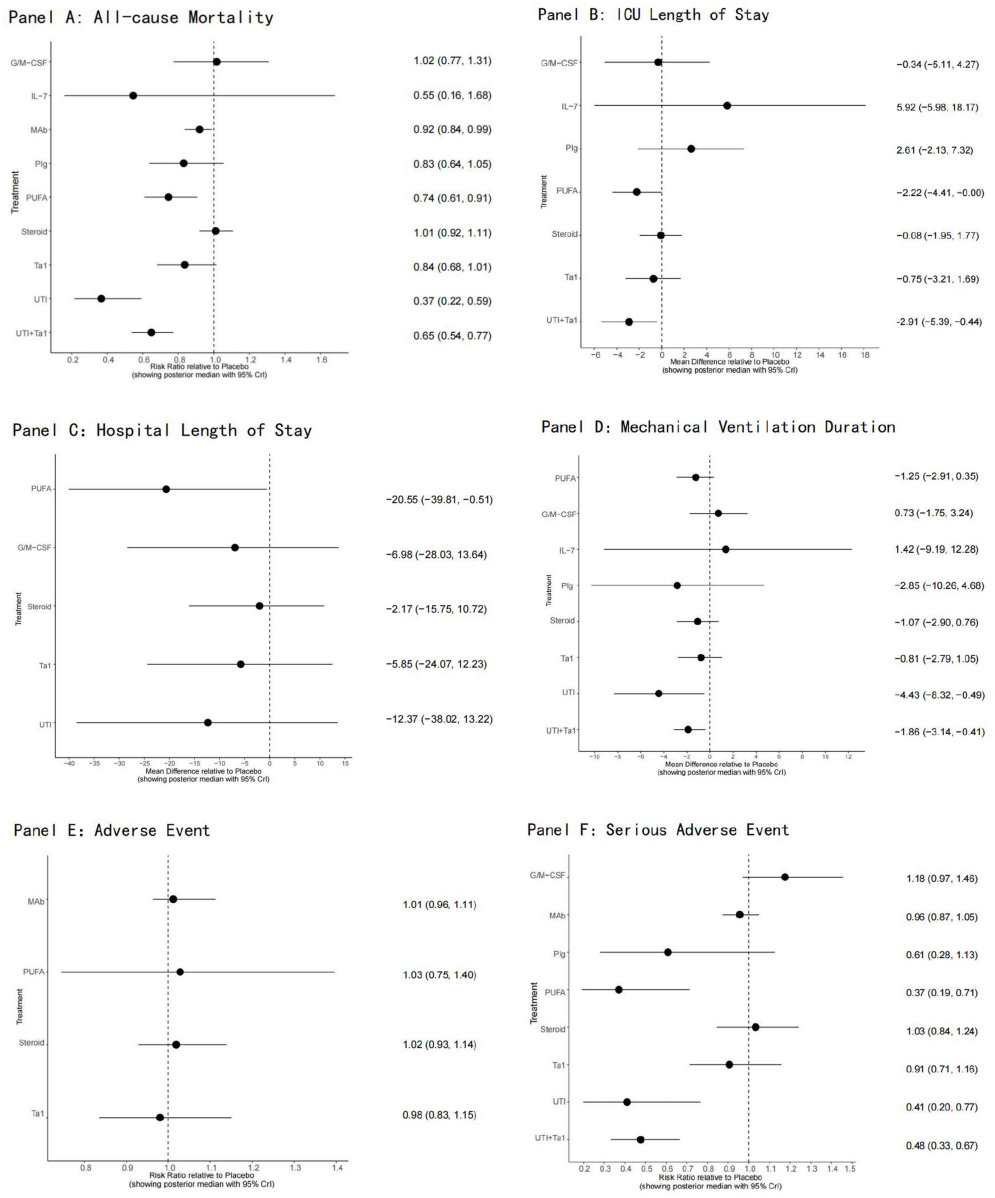
Forest plots for outcomes of immunotherapy. Panel **(A)**: All-cause Mortality; Panel **(B)**: ICU Length of Stay; Panel **(C)**: Hospital Length of Stay; Panel **(D)**: Mechanical Ventilation Duration; Panel **(E)**: Adverse Events; Panel **(F)**: Serious Adverse Events.

**Figure 5:**
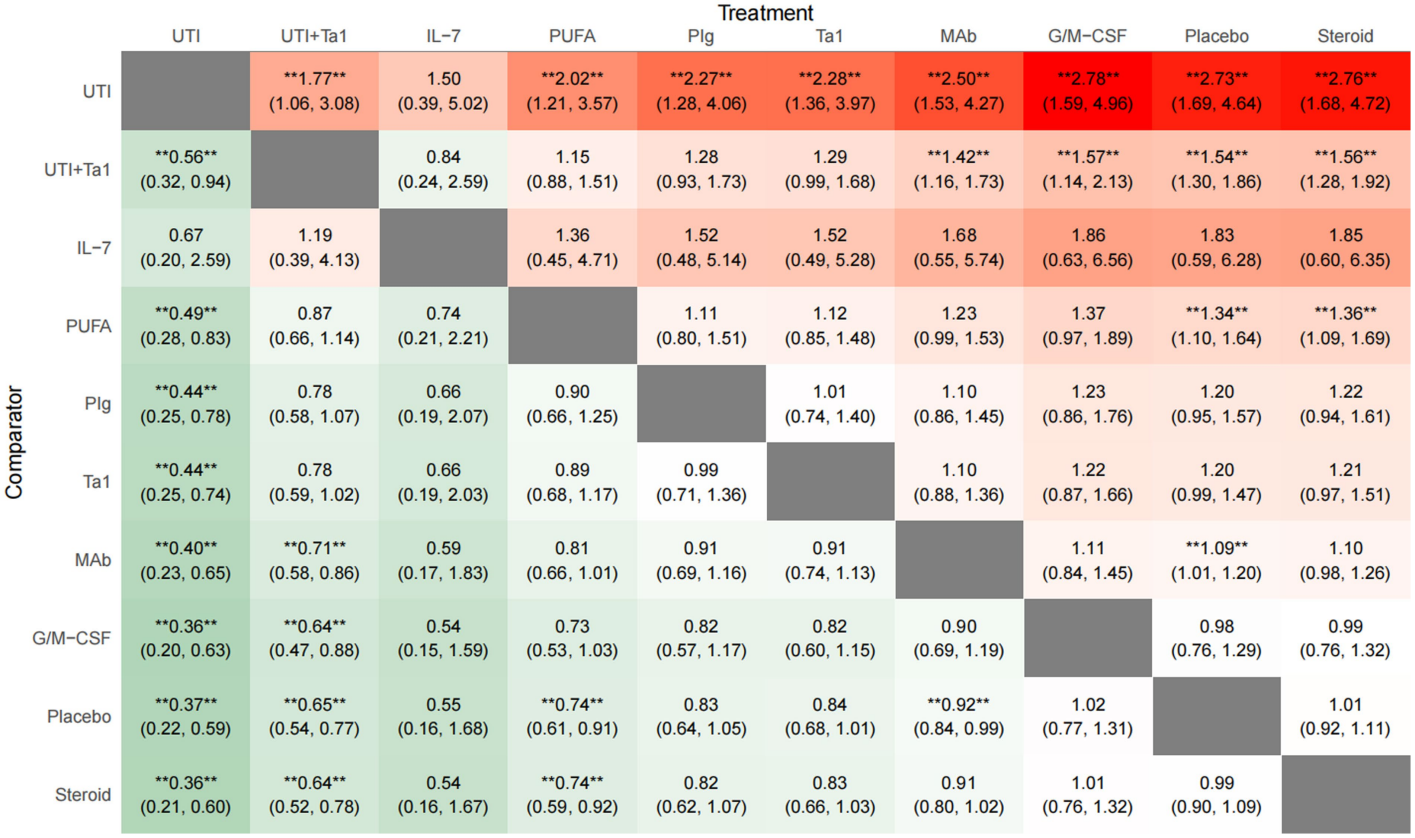
League heat table for all-cause mortality. Interventions are arranged from left to right (rows) and top to bottom (columns) in descending order of the Surface Under the Cumulative Ranking Curve (SUCRA) values. The leftmost/topmost intervention has the highest probability of being the best, while the rightmost/bottommost intervention has the lowest. The interventions are arranged in descending order of SUCRA values (from best to worst) along both the rows (left to right) and columns (top to bottom). The lower-left triangle (below the diagonal) reports the effect estimate for the row intervention versus the column intervention (RR < 1 favors the row intervention, shown in green). The upper-right triangle (above the diagonal) reports the effect estimate for the column intervention versus the row intervention (equivalent to the reciprocal of the lower-left triangle value, shown in red). Thus, the two triangles provide complementary perspectives on the same pairwise comparisons. Color intensity increases with effect magnitude. Darker shades indicate larger effect magnitudes (greater deviation of RR from 1); lighter shades indicate smaller differences between interventions. Statistical significance is indicated with an asterisk (*), defined by a 95% credible interval that does not include the null value (RR = 1).

**Figure 6:**
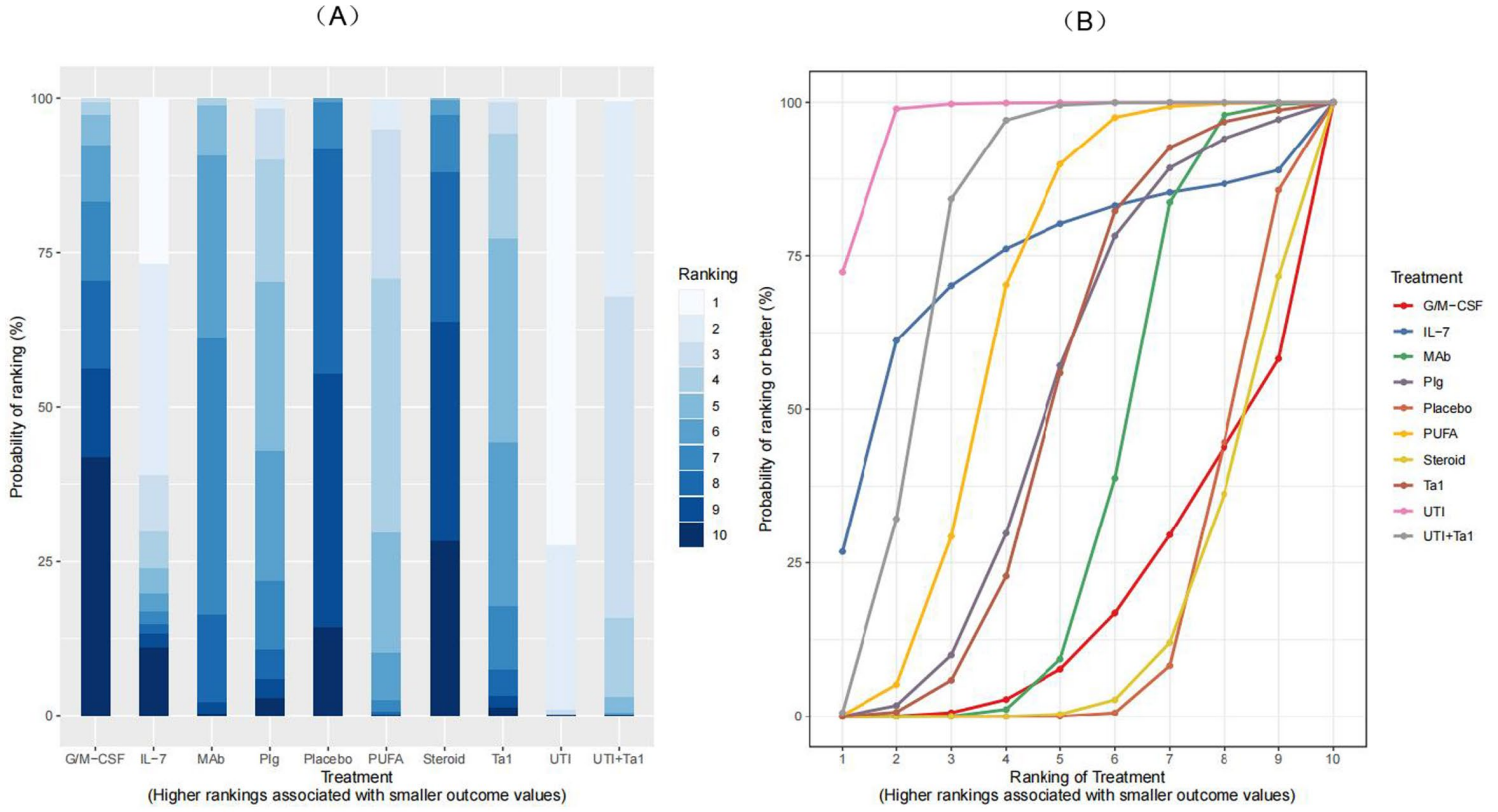
Treatment ranking for all-cause mortality. **(A)** Rank probabilities and **(B)** SUCRA plot for all immunomodulators. A larger SUCRA value indicates a better rank of treatment.

All 76 included trials provided data on all-cause mortality at the last follow-up, including 22,194 participants, of whom 7,659 (34.5%) died. A network plot for all-cause mortality is displayed in Figure 3A. According to the RRs with 95 %CIs (Figures 4A, 5), MAb (RR 0.92, 95% CrI 0.84–0.99), high certainty of evidence, PUFA (RR 0.74, 95% CrI 0.61–0.91), low certainty of evidence, UTI (RR 0.37, 95% CrI 0.22–0.59), low certainty of evidence and UTI plus Tα1 (RR 0.65, 95% CrI 0.54–0.77), low certainty of evidence most likely reduced all-cause mortality compared with the placebo. The rank probabilities of each intervention are shown in Figure 6A. Treatments ranking based on SUCRA values, from top to bottom, were as follows: UTI 96.76%, UTI + Tα1 79.28%, IL-7 73.16%, PUFA 65.72%, PIg 50.83%, Tα1 50.63%, MAb 36.74%, G/M-CSF 17.73%, Placebo 15.46% and Steroid 13.67% (Figure 6B). The Bayesian NMA summary of the findings with GRADE is presented in Table 2.

**Table 2:** Bayesian NMA summary of findings-all-cause mortality.

Total studies: 76, Total particiant:22,194	Relative effect (95% Crl)	Anticipated absolute effect (95% Crl)			Certainty of the evidence	Number of participants (trials)	Ranking (95% Crl)
		Placebo	Intervention	Risk difference			
MAb vs. Placebo	RR 0.92 (0.84–0.99) NMA estimate	386 per 1,000 (2086/5410 based on 18 trials)	356 per 1,000 (2,206/6200 based on 18 trials)	31 fewer per 1,000 (from 4 to 62 fewer)	⨁⨁⨁⨁ High	Direct evidence: 11,475 (18 trials)	7 (5–8)
PIg vs. Placebo	RR 0.83(0.64–1.05) NMA estimate	375 per 1,000 (78/208 based on 7 trials)	314 per 1,000 (66/210 based on 7 trials)	68 fewer per 1,000 (from 139 fewer to 19 more)	⨁⨁◯◯ Low Due to risk of bias and imprecision	Direct evidence: 418 (7 trials)	5 (3–10)
PUFA vs. Placebo	RR 0.74 (0.61–0.91) NMA estimate	309 per 1,000 (146/473 based on 10 trials)	246 per 1,000 (130/529 based on 10 trials)	80 fewer per 1,000 (from 28 to 121 fewer)	⨁⨁◯◯ Low Due to risk of bias and inconsistency	Direct evidence: 1,002 (10 trials)	4 (2–6)
Steroid vs. Placebo	RR 1.01 (0.92–1.11) NMA estimate	332 per 1,000 (821/2474 based on 18 trials)	349 per 1,000 (999/2861 based on 18 trials)	3 more per 1,000 (from 27 fewer to 37 more)	⨁⨁◯◯ Low Due to inconsistency and imprecision	Direct evidence: 5,335 (18 trials)	9 (7–10)
G/M-CSF vs. Placebo	RR 1.02 (0.77–1.31) NMA estimate	253 per 1,000 (120/474 based on 5 trials)	278 per 1,000 (131/472 based on 5 trials)	5 more per 1,000 (from 56 fewer to 81 more)	⨁⨁◯◯ Low Due to risk of bias and imprecision	Direct evidence: 946 (5 trials)	9 (5–10)
IL-7 vs. Placebo	RR 0.55 (0.16–1.68) NMA estimate	188 per 1,000 (3/16 based on 2 trials)	156 per 1,000 (5/32 based on 2 trials)	86 fewer per 1,000 (from 156 fewer to 135 more)	⨁◯◯◯ Very low Due to imprecision	Direct evidence: 48 (2 trials)	2 (1–10)
UTI vs. Placebo	RR 0.37 (0.22–0.59) NMA estimate	218 per 1,000 (45/206 based on 5 trials)	87 per 1,000 (17/196 based on 5 trials)	140 fewer per 1,000 (from 87 to 172 fewer)	⨁⨁◯◯ Low Due to risk of bias	Direct evidence: 402 (5 trials)	1 (1–2)
Ta1 vs. Placebo	RR 0.84 (0.68–1.01) NMA estimate	288 per 1,000 (235/815 based on 5 trials)	252 per 1,000 (204/810 based on 5 trials)	46 fewer per 1,000 (from 92 fewer to 3 more)	⨁⨁◯◯ Low Due to risk of bias	Direct evidence: 1,625 (5 trials)	5 (4–9)
UTI + Ta1 vs. Placebo	RR 0.65 (0.54–0.77) NMA estimate	473 per 1,000 (219/463 based on 6 trials)	308 per 1,000 (148/481 based on 6 trials)	166 fewer per 1,000 (from 109 to 218 fewer)	⨁⨁◯◯ Low Due to risk of bias	Direct evidence: 943 (6 trials)	3 (2–4)
Placebo	Reference comparator	/	/	/		/	9 (7–10)

GRADE Working Group grades of evidence (or certainty in the evidence).

High certainty: We possess a high degree of confidence that the true effect is situated in proximity to the estimated effect.

Moderate certainty: Our confidence in the effect estimate is moderate; it is probable that the true effect is near the estimated effect, although there exists a possibility of a substantial divergence.

Low certainty: our confidence in the effect estimate is constrained; the true effect may significantly differ from the estimated effect.

Very low certainty: we exhibit minimal confidence in the effect estimate; it is likely that the true effect diverges substantially from the estimated effect.

Risk of bias: limitations in study design or execution (e.g., lack of blinding, incomplete outcome data). Downgraded if serious limitations present.

Inconsistency: unexplained heterogeneity across studies (I^2^ > 50%) or incoherence between direct and indirect evidence. Downgraded if important inconsistency detected.

Indirectness: differences between study characteristics (population, intervention, comparator, outcome) and the target review question. Downgraded if important differences exist.

Imprecision: downgraded by one level for each of the following criteria met: (1) small sample size; (2) wide confidence/credible intervals; (3) confidence/credible intervals crossing the null value (RR = 1). One level = serious imprecision; two levels = very serious imprecision; three levels = extremely serious imprecision.

### ICU length of stay

A total of 29 trials with 5,633 patients reported data on ICU length of stay, and 7 immunomodulators were assessed (Figure 3B). Compared with placebo, UTI plus Tα1 (MD −2.91, 95% CrI −5.39–0.44; very low certainty) likely reduced the ICU length of stay (Figure 4B; Appendices 10A, 11A). UTI + Tα1 ranked the best according to the SUCRA statistic (89.31%), followed by PUFA (81.39%,) Tα1 (57.44%), G/M-CSF (49.88%), Steroid (44.08%), Placebo (41.06%), PIg (18.91%), and IL-7 (17.92%) (Appendix 9A).

### Hospital length of stay

Only a small subset of trials provided information on the hospital length of stay, with 11 RCTs (N = 3,720) involving 5 interventions connected to the evidence network (Figure 3C). Compared to placebo, only PUFA (MD −20.55, 95% Crl − 39.81 to −0.51; very low certainty) probably reduced hospital length of stay (Figure 4C; Appendices 10B, 11B). Treatments ranking based on SUCRA values, from largest to smallest, were as follows: PUFA 87.88%, UTI 66.1%, G/M-CSF 49.78%, Tα1 46.32%, Steroid 31.13% (Appendix 9B).

### Duration of mechanical ventilation

Eight interventions studied in 18 trials (*N* = 2,183) connected the evidence network (Figure 3D). Compared to placebo, duration of mechanical ventilation may be reduced by UTI (MD −4.43, 95% CrI −8.32 to −0.49; low certainty) and UTI plus Tα1 (MD −1.86, 95% CrI −3.14 to −0.41; very low certainty) (Figure 4D; Appendices 10C, 11C). Treatments ranking based on SUCRA values, from largest to smallest, were as follows: UTI 89.53%, UTI + Tα1 69.55%, PIg 66.55%, PUFA 55.57%, steroid 51.44%, Tα1 44.96%, IL-7 32.03%, placebo 24.59% and G/M-CSF 15.8% (Appendix 9C).

### Adverse events

A comprehensive analysis encompassing 17 studies with a total of 11,002 patients was conducted, with a focus on the incidence of adverse events (Figure 3E). Nonetheless, the available trials did not provide a sufficient basis for establishing an evidence network owing to the lack of comparable interventions. The treatment effect estimates derived from pairwise meta-analyses revealed that none of the interventions demonstrated efficacy in reducing adverse events compared with the placebo (Figure 4E).

### Serious adverse events

A total of 24 studies involving 13,843 patients were included in the analysis of serious adverse events (Figure 3F). Compared to placebo, PUFA (RR 0.37, 95% CrI 0.17–0.71; moderate certainty), UTI (RR 0.40, 95% CrI 0.20–0.72; moderate certainty) and UTI plus Tα1 (RR 0.48, 95% Crl 0.35–0.65; moderate certainty) probably reduce serious adverse events (Figure 4F; Appendices 10E, 11E). Treatments ranking based on SUCRA values, from largest to smallest, were as follows: PUFA 89.92%, UTI 87.04%, UTI + Tα1 78.79%, PIg 63.46%, Tα1 42.92%, MAb 38.59%, steroid 23.1%, placebo 22.77% and G/M-CSF 3.41% (Appendix 9E).

## Discussion

Sepsis is a clinical entity characterized by high incidence, elevated mortality, and substantial consumption of healthcare resources (3, 40). Its pathophysiology centers on a dysregulated host immune response, manifesting as a biphasic imbalance: an early hyperinflammatory phase followed by a state of immunosuppression (4–6). Traditional therapies, such as anti-infectives and supportive care, do not directly address this dynamic immune dysfunction (41). Consequently, immunomodulatory therapies have emerged as a promising adjunctive strategy. However, the comparative efficacy and safety of the various available immunomodulators remain unclear, and to our knowledge, no comprehensive network meta-analysis has previously synthesized this evidence. We therefore conducted this systematic review and network meta-analysis to compare the effects of commonly used immunomodulatory agents in patients with sepsis.

### Main findings

Our network meta-analysis of 76 randomized controlled trials, comprising 22,194 patients, yielded several key findings. First, ulinastatin, both as monotherapy and in combination with thymosin-α1, was associated with the most favorable outcomes, significantly reducing all-cause mortality, duration of mechanical ventilation, and serious adverse events. Notably, the combination of ulinastatin and thymosin-α1 also significantly shortened ICU length of stay. Second, n-3 polyunsaturated fatty acids (PUFAs) demonstrated significant benefits in reducing mortality, total hospital stay, and serious adverse events, although the effect sizes were smaller than those observed with ulinastatin. Third, monoclonal antibodies (MAbs) were associated with a reduction in all-cause mortality, supported by high-certainty evidence. Finally, other immunomodulators, including corticosteroids, polyclonal immunoglobulins, G/GM-CSF, IL-7, and thymosin-α1 alone, did not demonstrate statistically significant benefits on the primary outcome of mortality in this analysis.

Our findings extend and contextualize previous research in several important ways. For instance, while a recent large trial of thymosin-*α*1 (TESTS) did not show a mortality benefit (24), our analysis suggests that its combination with ulinastatin may be more effective, potentially due to synergistic effects on both the innate and adaptive immune systems. Furthermore, our results align with prior evidence suggesting that targeting the hyperinflammatory phase with agents like ulinastatin and PUFAs may be beneficial, while also highlighting that agents primarily modulating adaptive immunity (e.g., G-CSF, IL-7) did not show overall benefits in this unselected sepsis population.

The potential mechanisms underlying the observed benefits of specific immunomodulators are rooted in the biphasic pathophysiology of sepsis. Ulinastatin, a urinary trypsin inhibitor, exerts potent anti-inflammatory effects by inhibiting the NF-κB pathway, stabilizing lysosomal membranes, and reducing the production of pro-inflammatory cytokines such as TNF-α and IL-1β (42, 43). Its ability to mitigate the initial cytokine storm may explain its impact on early mortality and organ dysfunction. The addition of thymosin-α1, a peptide that enhances T-cell function, may address the subsequent immunosuppressive phase, providing a rationale for the improved outcomes observed with the combination (44, 45). Similarly, n-3 PUFAs are known to suppress cytokine expression and have been shown to improve lymphocyte counts and reduce organ injury (46–48). Monoclonal antibodies achieve precise treatment by targeting specific immune molecules. They act via two main strategies: one group of mAbs targets pathogens or their components, whereas the other group targets inflammatory signals to directly curb the production of inflammatory mediators (49).

In contrast, the lack of clear benefit for other agents, such as corticosteroids or G-CSF, may reflect the complex, time-dependent nature of the immune response, where general immunosuppression or stimulation may be detrimental depending on the phase of illness. G-CSF and GM-CSF function to stimulate hematopoietic stem/progenitor cells (HSPCs) in the bone marrow, thereby promoting the generation and differentiation of neutrophils and monocyte-macrophages, respectively (50). Although G-CSF helps control infection in early sepsis by enhancing innate immunity, the impaired responsiveness of HSPCs to G-CSF during the ensuing immunosuppressive phase may contribute to an increased risk of secondary infections (51). A similar principle applies to corticosteroids. By inhibiting the excessive inflammation triggered by TLR7/8 agonists, corticosteroids help control the immunoinflammatory response in sepsis (52). Despite their widespread clinical utility, corticosteroids are associated with multiple adverse effects, including immunosuppression. Prolonged oral corticosteroids (OCSs) administration impairs leukocyte function and inhibits transcription factors, leading to marked CD4 + lymphopenia and frequently reduced serum immunoglobulin G (IgG) levels (53). If administered during the immunosuppressive phase, the aforementioned factors may substantially elevate the risk of infection.

Most of the adverse events (AEs) and serious adverse events (SAEs) reported in the included trials were disease-related complications of sepsis (e.g., septic shock, multiple organ dysfunction syndrome, acute kidney injury, disseminated intravascular coagulation, and death), rather than drug-related adverse reactions. Drug-related AEs were rarely reported across the included studies, precluding a meaningful quantitative analysis of drug safety. Therefore, our analysis of AEs/SAEs should be interpreted as reflecting the impact of immunomodulatory therapies on sepsis-related complications, rather than their direct safety profiles. A reduction in these events likely indicates improvement in the underlying disease course.

### Strengths and limitations

This study has several strengths. It is the first network meta-analysis to comprehensively compare a wide range of immunomodulatory agents for sepsis, incorporating data from 76 RCTs and 22,194 patients. The primary comparison in this research, mirroring that of most RCTs cited, was between immunomodulatory therapy and placebo. However, unlike previous pairwise meta-analyses that focused on single agents, such as corticosteroids (19) or ulinastatin (54), our study allows for indirect comparisons across therapies. We employed rigorous methodology, including a comprehensive literature search, dual independent review, and a Bayesian framework for analysis. The use of the GRADE system to rate the certainty of evidence for each comparison adds a critical dimension to the interpretation of our findings, highlighting where confidence in the effect estimates is high and where it remains limited.

However, our review also has important limitations that should be acknowledged. First, and most significantly, the certainty of evidence for most comparisons was rated as low or very low, primarily due to risk of bias (particularly lack of blinding) and imprecision. This limits the strength of recommendations that can be made based on these findings. Second, although we performed a secondary update of the literature search, the search cutoff date was set to March 31, 2025, due to the substantial workload and limited personnel resources. A third round of search update would have considerably prolonged the revision timeline. To ensure transparency, we have provided the full search strategy for each database in Appendix 1, allowing readers to assess the comprehensiveness of our search and to refine it in future updates. Third, there was considerable heterogeneity across the included trials in terms of patient populations, sepsis definitions (e.g., SIRS vs. Sepsis-3), and co-interventions, which may affect the transitivity assumption underpinning the network meta-analysis. Fourth, we were unable to perform subgroup analyses based on immune phenotype (e.g., hyperinflammatory vs. immunosuppressed) or disease severity due to the lack of individual patient data, which may be crucial as the effect of immunomodulation likely differs across patient subgroups (52, 55). Fifth, the network structure was largely based on indirect evidence; for many comparisons, there were few or no direct head-to-head trials, which can introduce statistical inconsistency. Furthermore, formal assessment of incoherence using node-splitting methods was not possible due to the absence of closed loops in the network structure. Sixth, the definition and reporting of SAEs or AEs varied across the included trials, as no universal standardization exists. This heterogeneity may affect the comparability of SAE outcomes across studies. Besides, many trials did not systematically assess for harm, potentially underestimating safety concerns associated with these agents (56–58). Seventh, protocol registration (e.g., in PROSPERO) was not performed prior to study initiation, as this was not mandatory at the time of study conception. Nevertheless, we have adhered to PRISMA-NMA reporting guidelines to ensure methodological transparency. Eighth, when original studies reported only medians and interquartile ranges (IQRs), we converted these to means and standard deviations (SDs) using the method proposed by Luo et al. (36). We acknowledge that this conversion assumes that the underlying data follow a normal distribution, an assumption that could not be verified without access to individual patient data. Finally, publication bias cannot be completely ruled out, although we were unable to formally assess this for many comparisons due to the limited number of trials per direct comparison.

Our findings highlight several avenues for future research. The most pressing need is for large, well-designed, multicenter randomized controlled trials that directly compare the most promising interventions—ulinastatin (alone and in combination with thymosin-α1), PUFAs, and MAbs—against each other and against placebo. Such trials should be designed with rigorous methodological standards, including blinding and complete outcome reporting. Crucially, future studies should incorporate a precision medicine approach, stratifying patients by their immune status (e.g., using biomarkers such as mHLA-DR, PD-1 expression, or cytokine profiles) to identify which patients are most likely to benefit from a given immunomodulatory strategy. This would move the field beyond a one-size-fits-all approach and toward personalized immunomodulation. Furthermore, standardized definitions and systematic collection of adverse events are essential to accurately characterize the safety profile of these therapies.

## Conclusion

To our knowledge, this network meta-analysis represents the first comprehensive comparative evaluation of a wide spectrum of immunomodulatory therapies for sepsis. Our findings indicate that ulinastatin, both as a monotherapy and in combination with thymosin-α1, is associated with the most pronounced reductions in all-cause mortality, duration of mechanical ventilation, and serious adverse events, with combination therapy also significantly shortening the ICU length of stay. Furthermore, n-3 polyunsaturated fatty acids (PUFAs) demonstrated significant benefits in lowering mortality, reducing total hospital stay, and decreasing serious adverse events. Monoclonal antibodies (MAbs) were also found to reduce mortality, an outcome supported by high-quality evidence. Despite these insights, the evidence for many interventions remains limited by the low to very low certainty of findings. Therefore, future research should prioritize large-scale, multi-center randomized controlled trials with direct comparisons between the most promising interventions to generate higher-quality evidence and conclusively establish their roles in clinical practice.

## Data Availability

The original contributions presented in the study are included in the article/Supplementary material, further inquiries can be directed to the corresponding authors.

## Glossary

AE: adverse event
CI: confidence interval
CrI: credible interval
DIC: deviance information criterion
G-CSF: granulocyte colony-stimulating factor
G/GM-CSF: granulocyte/macrophage colony-stimulating factor
GM-CSF: granulocyte-macrophage colony-stimulating factor
GRADE: Grading of Recommendations, Assessment, Development, and Evaluation
HLA-DR: human leukocyte antigen-DR
HSPCs: hematopoietic stem/progenitor cells
ICU: intensive care unit
ICU-LOS: length of intensive care unit stay
IFN-*γ*: interferon-gamma
IL: interleukin
IL-1β: interleukin-1 beta
IL-2: interleukin-2
IL-6: interleukin-6
IL-7: interleukin-7
IQR: interquartile range
LOS: length of stay
LPS: lipopolysaccharide
MAb: monoclonal antibody
MD: mean difference
MV duration: duration of mechanical ventilation
NF-κB: nuclear factor-kappa B
NMA: network meta-analysis
OCS: oral corticosteroid
PD-1: programmed death-1
PD-L1: programmed death-ligand 1
PICOS: Population-Intervention-Control-Outcomes-Studies
PIg: polyclonal immunoglobulin
PRISMA-NMA: Preferred Reporting Items for Systematic Reviews and Meta-Analyses for Network Meta-Analyses
PUFA: n-3 polyunsaturated fatty acid
RCT: randomized controlled trial
RoB: risk of bias
RR: risk ratio
SAE: serious adverse event
SD: standard deviation
SOC: standard of care
SUCRA: surface under the cumulative ranking curve
T*α*1: thymosin-α1
Th1: T helper type 1
TNF-α: tumor necrosis factor-alpha
UTI: ulinastatin

## References

[1] Singer M Deutschman CS Seymour CW Shankar-Hari M Annane D Bauer M . The third international consensus definitions for sepsis and septic shock (sepsis-3). JAMA. (2016) 315:801–10. doi: 10.1001/jama.2016.0287, 26903338 PMC4968574

[2] Evans L Rhodes A Alhazzani W Antonelli M Coopersmith CM French C . Surviving sepsis campaign: international guidelines for management of sepsis and septic shock 2021. Intensive Care Med. (2021) 47:1181–247. doi: 10.1007/s00134-021-06506-y, 34599691 PMC8486643

[3] Rudd KE Johnson SC Agesa KM Shackelford KA Tsoi D Kievlan DR . Global, regional, and national sepsis incidence and mortality, 1990-2017: analysis for the global burden of disease study. Lancet. (2020) 395:200–11. doi: 10.1016/S0140-6736(19)32989-7, 31954465 PMC6970225

[4] Nedeva C. Inflammation and cell death of the innate and adaptive immune system during sepsis. Biomolecules. (2021) 11:1011. doi: 10.3390/biom11071011, 34356636 PMC8301842

[5] Qiu Y Tu GW Ju MJ Yang C Luo Z. The immune system regulation in sepsis: from innate to adaptive. Curr Protein Pept Sci. (2019) 20:799–816. doi: 10.2174/1389203720666190305164128, 30843486

[6] Liu D Huang SY Sun JH Zhang HC Cai QL Gao C . Sepsis-induced immunosuppression: mechanisms, diagnosis and current treatment options. Mil Med Res. (2022) 9:56. doi: 10.1186/s40779-022-00422-y, 36209190 PMC9547753

[7] Jacobi J. The pathophysiology of sepsis-2021 update: part 1, immunology and coagulopathy leading to endothelial injury. Am J Health Syst Pharm. (2022) 79:329–37. doi: 10.1093/ajhp/zxab380, 34605875 PMC8500113

[8] Mollnes TE Huber-Lang M. Complement in sepsis-when science meets clinics. FEBS Lett. (2020) 594:2621–32. doi: 10.1002/1873-3468.13881, 32621378

[9] de Nooijer AH Kotsaki A Kranidioti E Kox M Pickkers P Toonen EJM . Complement activation in severely ill patients with sepsis: no relationship with inflammation and disease severity. Crit Care. (2023) 27:63. doi: 10.1186/s13054-023-04344-6, 36797757 PMC9933299

[10] Jiang H Guo Y Wang Q Wang Y Peng D Fang Y . The dysfunction of complement and coagulation in diseases: the implications for the therapeutic interventions. MedComm (2020). (2024) 5:e785. doi: 10.1002/mco2.785, 39445002 PMC11496570

[11] Langston JC Rossi MT Yang Q Ohley W Perez E Kilpatrick LE . Omics of endothelial cell dysfunction in sepsis. Vasc Biol. (2022) 4:R15–34. doi: 10.1530/VB-22-0003, 35515704 PMC9066943

[12] Fattahi F Grailer JJ Parlett M Lu H Malan EA Abe E . Requirement of complement C6 for intact innate immune responses in mice. J Immunol. (2020) 205:251–60. doi: 10.4049/jimmunol.1900801, 32444389

[13] Cicchinelli S Pignataro G Gemma S Piccioni A Picozzi D Ojetti V . PAMPs and DAMPs in sepsis: a review of their molecular features and potential clinical implications. Int J Mol Sci. (2024) 25:962. doi: 10.3390/ijms25020962, 38256033 PMC10815927

[14] Zhang W Fang X Gao C Song C He Y Zhou T . MDSCs in sepsis-induced immunosuppression and its potential therapeutic targets. Cytokine Growth Factor Rev. (2023) 69:90–103. doi: 10.1016/j.cytogfr.2022.07.007, 35927154

[15] Chen G Chong H Zhang P Wen D Du J Gao C . An integrative model with HLA-DR, CD64, and PD-1 for the diagnostic and prognostic evaluation of sepsis. Immun Inflamm Dis. (2024) 12:e1138. doi: 10.1002/iid3.1138, 38270311 PMC10777881

[16] Tang J Shang C Chang Y Jiang W Xu J Zhang L . Peripheral PD-1+NK cells could predict the 28-day mortality in sepsis patients. Front Immunol. (2024) 15:1426064. doi: 10.3389/fimmu.2024.1426064, 38953031 PMC11215063

[17] Rao M McGonagill PW Brackenridge S Remy KE Caldwell CC Hotchkiss RS . Funcional immunophenotyping for precision therapies in sepsis. Shock. (2024) 63:189–201. doi: 10.1097/SHK.0000000000002511 39617419 PMC12447363

[18] Zhang Q. Immune dysfunction and immuno-modulatory therapy on sepsis. Chin J Appl Clin Pediatr. (2015) 30:405–8. doi: 10.3760/cma.j.issn.2095-428X.2015.06.002

[19] Lazar A. Recent data about the use of corticosteroids in sepsis—review of recent literature. Biomedicine. (2024) 12:984. doi: 10.3390/biomedicines12050984 PMC11118609 38790946

[20] Min Y He Y Tuo L Fan M Xie Y. Application of a combination of lactated ringer's solution and ulinastatin for early resuscitation in sepsis. Trop J Pharm Res. (2023) 22:2525–30. doi: 10.4314/tjpr.v22i12.15

[21] Akatsuka M Masuda Y Tatsumi H Sonoda T. Efficacy of intravenous immunoglobulin therapy for patients with sepsis and low immunoglobulin G levels: a single-center retrospective study. Clin Ther. (2022) 44:295–303. doi: 10.1016/j.clinthera.2021.12.008 35000795

[22] Monastra L Viola G Fraganza F. IgM-enriched immunoglobulin versus standard therapy in the meningococcal sepsis: a retrospective study. J Biol Regul Homeost Agents. (2022) 36:795–6.

[23] Klinkmann G Altrichter J Reuter DA Mitzner S. Therapeutic apheresis in sepsis. Ther Apher Dial. (2022) 26:64–72. doi: 10.1111/1744-9987.13815 36468315

[24] Wu J Pei F Zhou L Li W Sun R Li Y . The efficacy and safety of thymosin α1 for sepsis (TESTS): multicentre, double blinded, randomized, placebo controlled, phase 3 trial. BMJ. (2025) 388:e082583. doi: 10.1136/bmj-2024-082583 39814420 PMC11780596

[25] Bo L Wang F Zhu J Li J Deng X. Granulocyte-colony stimulating factor (G-CSF) and granulocyte-macrophage colony stimulating factor (GM-CSF) for sepsis: a meta-analysis. Crit Care. (2011) 15:R58. doi: 10.1186/cc10031, 21310070 PMC3221991

[26] Gao DN Yang ZX Qi QH. Roles of PD-1, Tim-3 and CTLA-4 in immunoregulation in regulatory T cells among patients with sepsis. Int J Clin Exp Med. (2015) 8:18998. 26770525 PMC4694425

[27] Unsinger J Mcglynn M Kasten KR Hoekzema AS Watanabe E Muenzer JT . IL-7 promotes T cell viability, trafficking, and functionality and improves survival in sepsis. J Immunol. (2010) 184:3768–79. doi: 10.4049/jimmunol.0903151, 20200277 PMC2914630

[28] Angus DC Birmingham MC Balk RA Scannon PJ Collins D Kruse JA. E5 murine monoclonal antiendotoxin antibody in gram-negative sepsis: a randomized controlled trial. JAMA. (2000) 283:1723–30. doi: 10.1001/jama.283.13.1723 10755499

[29] Abraham E Wunderink R Silverman H Perl TM Nasraway S Levy H . Efficacy and safety of monoclonal antibody to human tumor necrosis factor alpha in patients with sepsis syndrome. A randomized, controlled, double-blind, multicenter clinical trial. JAMA J Am Med Assoc. (1995) 273:934–41. 7884952

[30] Chen H Wang S Zhao Y Luo Y Tong H Su L. Correlation analysis of omega-3 fatty acids and mortality of sepsis and sepsis-induced ARDS in adults: data from previous randomized controlled trials. Nutr J. (2018) 17:57. doi: 10.1186/s12937-018-0356-8, 29859104 PMC5984323

[31] Amir-Behghadami M Janati A. Population, intervention, comparison, outcomes and study (PICOS) design as a framework to formulate eligibility criteria in systematic reviews. Emerg Med J. (2020) 37:387. doi: 10.1136/emermed-2020-209567, 32253195

[32] Higgins JP Altman DG Gøtzsche PC Jüni P Moher D Oxman AD . The Cochrane collaboration's tool for assessing risk of bias in randomised trials. BMJ. (2011) 343:d5928. doi: 10.1136/bmj.d5928 22008217 PMC3196245

[33] Puhan MA Schünemann HJ Murad MH Li T Brignardello-Petersen R Singh JA . A GRADE working group approach for rating the quality of treatment effect estimates from network meta-analysis. BMJ. (2014) 349:g5630. doi: 10.1136/bmj.g5630, 25252733

[34] Santesso N Glenton C Dahm P Garner P Akl EA Alper B . GRADE guidelines 26: informative statements to communicate the findings of systematic reviews of interventions. J Clin Epidemiol. (2020) 119:126–35. doi: 10.1016/j.jclinepi.2019.10.014, 31711912

[35] Béliveau A Boyne DJ Slater J Brenner D Arora P. BUGSnet: an R package to facilitate the conduct and reporting of Bayesian network meta-analyses. BMC Med Res Methodol. (2019) 19:196. doi: 10.1186/s12874-019-0829-2, 31640567 PMC6805536

[36] Luo D Wan X Liu J Tong T. How to estimate the sample mean and standard deviation from the sample size, median, extremes or quartiles? Chin J Evid Based Med. (2017) 17:1350–6. doi: 10.7507/1672-2531.201706060

[37] Donegan S Williamson P D'Alessandro U Tudur Smith C. Assessing key assumptions of network meta-analysis: a review of methods. Res Synth Methods. (2013) 4:291–323. doi: 10.1002/jrsm.1085, 26053945

[38] Dias S Sutton AJ Ades AE Welton NJ. Evidence synthesis for decision making 2: a generalized linear modeling framework for pairwise and network meta-analysis of randomized controlled trials. Med Decis Mak. (2013) 33:607–17. doi: 10.1177/0272989X12458724, 23104435 PMC3704203

[39] Higgins JP Thompson SG. Quantifying heterogeneity in a meta-analysis. Stat Med. (2002) 21:1539–58. doi: 10.1002/sim.1186, 12111919

[40] Chen H Lu B Xu Y Li N Zhou Y Ma X . Trend in sepsis burden among hospitalized non-child cancer patients in China, 2017-2019: a nationwide cross-sectional study. Med Plus. (2024) 1:100062. doi: 10.1016/j.medp.2024.100062 38702275

[41] Giamarellos-Bourboulis EJ Aschenbrenner AC Bauer M Bock C Calandra T Gat-Viks I . The pathophysiology of sepsis and precision-medicine-based immunotherapy. Nat Immunol. (2024) 25:19–28. doi: 10.1038/s41590-023-01660-5, 38168953

[42] Masui M Suzuki M Fujise Y Kanayama N. Calcium-induced changes in chondroitin sulfate chains of urinary trypsin inhibitor. Biochim Biophys Acta. (2001) 1546:261–7. doi: 10.1016/S0167-4838(00)00259-4, 11295432

[43] Li ST Dai Q Zhang SX Liu YJ Yu QQ Tan F . Ulinastatin attenuates LPS-induced inflammation in mouse macrophage RAW264.7 cells by inhibiting the JNK/NF-κB signaling pathway and activating the PI3K/Akt/Nrf2 pathway. Acta Pharmacol Sin. (2018) 39:1294–304. doi: 10.1038/aps.2017.143, 29323338 PMC6289329

[44] Gu B Zhou Y Nie Y Wang L Liang L Liao Z . Efficacy of thymosin α1 for sepsis: a systematic review and meta-analysis of randomized controlled trials. Front Cell Infect Microbiol. (2025) 15:1673959. doi: 10.3389/fcimb.2025.1673959, 40969554 PMC12440967

[45] Goldstein AL Low TL Thurman GB Zatz MM Hall N Chen J . Current status of thymosin and other hormones of the thymus gland. Recent Prog Horm Res. (1981) 37:369–415. doi: 10.1016/B978-0-12-571137-1.50012-8 7025134

[46] Lopez-Garcia E Schulze MB Manson JE Meigs JB Albert CM Rifai N . Consumption of (n-3) fatty acids is related to plasma biomarkers of inflammation and endothelial activation in women. J Nutr. (2004) 134:1806–11. doi: 10.1093/jn/134.7.1806, 15226473

[47] Chen H Wang W Hong C Zhang M Hong Y Wang S . Omega-3 fish oil reduces mortality due to severe sepsis with acute gastrointestinal injury grade III. Pharmacogn Mag. (2017) 13:407–12. doi: 10.4103/pm.pm_418_16, 28839364 PMC5551357

[48] Chen YL Xie YJ Liu ZM Chen WB Zhang R Ye HX . Omega-3 fatty acids impair miR-1-3p-dependent Notch3 down-regulation and alleviate sepsis-induced intestinal injury. Mol Med. (2022) 28:9. doi: 10.1186/s10020-021-00425-w, 35090386 PMC8796544

[49] Kharga K Kumar L Patel SKS. Recent advances in monoclonal antibody-based approaches in the management of bacterial sepsis. Biomedicine. (2023) 11:765. doi: 10.3390/biomedicines11030765, 36979744 PMC10045367

[50] Greenbaum AM Link DC. Mechanisms of G-CSF-mediated hematopoietic stem and progenitor mobilization. Leukemia. (2011) 25:211–7. doi: 10.1038/leu.2010.248, 21079612

[51] Goedhart M Slot E Pascutti MF Geerman S Rademakers T Nota B . Bone marrow harbors a unique population of dendritic cells with the potential to boost neutrophil formation upon exposure to fungal antigen. Cells. (2021) 11:55. doi: 10.3390/cells11010055, 35011617 PMC8750392

[52] Walton AH Mazer MB Remy KE Osborne DF Davitt EB Griffith TS . Determining potential immunomodulatory drug efficacy in sepsis using ELISpot. Sci Rep. (2025) 15:13464. doi: 10.1038/s41598-025-92016-6, 40251188 PMC12008245

[53] Mustafa SS. Steroid-induced secondary immune deficiency. Ann Allergy Asthma Immunol. (2023) 130:713–7. doi: 10.1016/j.anai.2023.01.010, 36681272

[54] Wang H Liu B Tang Y Chang P Yao L Huang B . Improvement of sepsis prognosis by ulinastatin: a systematic review and meta-analysis of randomized controlled trials. Front Pharmacol. (2019) 10:1370. doi: 10.3389/fphar.2019.01370, 31849646 PMC6893897

[55] Cajander S Kox M Scicluna BP Weigand MA Mora RA Flohé SB . Profiling the dysregulated immune response in sepsis: overcoming challenges to achieve the goal of precision medicine. Lancet Respir Med. (2024) 12:305–22. doi: 10.1016/S2213-2600(23)00330-2, 38142698

[56] Koshi EJ Young K Mostales JC Vo KB Burgess LP. Complications of corticosteroid therapy: a comprehensive literature review. J Pharm Technol. (2022) 38:360–7. doi: 10.1177/87551225221116266, 36311302 PMC9608099

[57] Zhang S Chen W Zhou J Liang Q Zhang Y Su M . The benefits and safety of monoclonal antibodies: implications for Cancer immunotherapy. J Inflamm Res. (2025) 18:4335–57. doi: 10.2147/JIR.S499403, 40162076 PMC11952073

[58] Guo Y Tian X Wang X Xiao Z. Adverse effects of immunoglobulin therapy. Front Immunol. (2018) 9:1299. doi: 10.3389/fimmu.2018.01299, 29951056 PMC6008653

