# Supplementary material for: Comparative efficacy and safety of immunomodulatory therapies for sepsis: a systematic review and network meta-analysis
Source: Front Med (Lausanne). 2026 May 12;13:1808427. doi: 10.3389/fmed.2026.1808427 (PMC13201415; doi:10.3389/fmed.2026.1808427)
Supplement: Supplementary file 1 [file Supplementary_file_1.docx]

**Appendix 1: Search strategies**

**PubMed**

(("immunomodulatory therapy" OR "immune modulation" OR "immunomodulation" OR "immunotherapy" OR "corticosteroids" OR "Thymosin" OR "PD-1" OR "PD-L1" OR "Interleukin-7" OR "immunoglobulin" OR "Granulocyte-Colony Stimulating Factor" OR "urinastatin" OR "omega-3 fatty acid" OR "fish oil" OR "immunomodulatory agents"[MeSH] OR "immunomodulation"[MeSH] OR "immunotherapy"[MeSH] OR "corticosteroids"[MeSH] OR "Thymosin"[MeSH] OR "Programmed Cell Death 1 Receptor"[MeSH] OR "Programmed Cell Death 1 Ligand 1"[MeSH] OR "Interleukin-7"[MeSH] OR "immunoglobulins"[MeSH] OR "Granulocyte Colony-Stimulating Factor"[MeSH] OR "urinastatin"[MeSH] OR "omega-3 fatty acids"[MeSH] OR "fish oils"[MeSH]) AND ("sepsis" OR "septicemia" OR "septic shock" OR "septic" OR "sepsis"[MeSH] OR "septicemia"[MeSH] OR "septic shock"[MeSH] OR "septic"[MeSH])) AND (randomized controlled trial[pt] OR controlled clinical trial[pt] OR randomized[tiab] OR placebo[tiab] OR drug therapy[sh] OR randomly[tiab] OR trial[tiab] OR groups[tiab]) NOT (animals[mh] NOT humans[mh])

**Embase**

('immunomodulatory therapy'/exp OR 'immune modulation'/exp OR 'immunomodulation'/exp OR 'immunotherapy'/exp OR 'corticosteroids'/exp OR 'Thymosin'/exp OR 'PD-1'/exp OR 'PD-L1'/exp OR 'Interleukin-7'/exp OR 'immunoglobulin'/exp OR 'Granulocyte-Colony Stimulating Factor'/exp OR 'urinastatin'/exp OR 'omega-3 fatty acid'/exp OR 'fish oil'/exp) AND ('sepsis'/exp OR 'septicemia'/exp OR 'septic shock'/exp OR 'septic'/exp) AND ('crossover procedure':de OR 'double-blind procedure':de OR 'randomized controlled trial':de OR 'single-blind procedure':de OR (random* OR factorial* OR crossover* OR cross NEXT/1 over* OR placebo* OR doubl* NEAR/1 blind* OR singl* NEAR/1 blind* OR assign* OR allocat* OR volunteer*):de,ab,ti)

**Cochrane**

("immunomodulatory therapy" OR "immune modulation" OR "immunomodulation" OR "immunotherapy" OR "corticosteroids" OR "Thymosin" OR "PD-1" OR "PD-L1" OR "Interleukin-7" OR "immunoglobulin" OR "Granulocyte-Colony Stimulating Factor" OR "urinastatin" OR "omega-3 fatty acid" OR "fish oil") AND ("sepsis" OR "septicemia" OR "septic shock" OR "septic") AND (randomized controlled trial[pt] OR controlled clinical trial[pt] OR randomized[tiab] OR placebo[tiab] OR drug therapy[sh] OR randomly[tiab] OR trial[tiab] OR groups[tiab]) NOT (animals[mh] NOT humans[mh])

**Appendix 2: References to trials included in the network meta-analysis**

1. Gallagher J, Fisher C, Sherman B, et al. A multicenter, open-label, prospective, randomized, dose-ranging pharmacokinetic study of the anti-TNF-alpha antibody afelimomab in patients with sepsis syndrome. *Intensive Care Medicine* 2001; **27**(7): 1169-78.

2. Cohen J, Carlet J. INTERSEPT: an international, multicenter, placebo-controlled trial of monoclonal antibody to human tumor necrosis factor-alpha in patients with sepsis. International Sepsis Trial Study Group. *Crit Care Med* 1996; **24**(9): 1431-40.

3. Panacek EA, Marshall JC, Albertson TE, et al. Efficacy and safety of the monoclonal anti-tumor necrosis factor antibody F(ab')2 fragment afelimomab in patients with severe sepsis and elevated interleukin-6 levels. *Crit Care Med* 2004; **32**(11): 2173-82.

4. Dhainaut JF, Vincent JL, Richard C, et al. CDP571, a humanized antibody to human tumor necrosis factor-alpha: safety, pharmacokinetics, immune response, and influence of the antibody on cytokine concentrations in patients with septic shock. CPD571 Sepsis Study Group. *Crit Care Med* 1995; **23**(9): 1461-9.

5. Abraham E, Anzueto A, Gutierrez G, et al. Double-blind randomised controlled trial of monoclonal antibody to human tumour necrosis factor in treatment of septic shock. NORASEPT II Study Group. *Lancet* 1998; **351**(9107): 929-33.

6. Clark MA, Plank LD, Connolly AB, et al. Effect of a chimeric antibody to tumor necrosis factor-alpha on cytokine and physiologic responses in patients with severe sepsis--a randomized, clinical trial. *Crit Care Med* 1998; **26**(10): 1650-9.

7. Abraham E, Wunderink R, Silverman H, et al. Efficacy and safety of monoclonal antibody to human tumor necrosis factor alpha in patients with sepsis syndrome. A randomized, controlled, double-blind, multicenter clinical trial. TNF-alpha MAb Sepsis Study Group. *JAMA* 1995; **273**(12): 934-41.

8. Reinhart K, Menges T, Gardlund B, et al. Randomized, placebo-controlled trial of the anti-tumor necrosis factor antibody fragment afelimomab in hyperinflammatory response during severe sepsis: The RAMSES Study. *Crit Care Med* 2001; **29**(4): 765-9.

9. Angus DC, Birmingham MC, Balk RA, et al. E5 murine monoclonal antiendotoxin antibody in gram-negative sepsis: a randomized controlled trial. E5 Study Investigators. *JAMA* 2000; **283**(13): 1723-30.

10. Wortel CH, von der Möhlen MA, van Deventer SJ, et al. Effectiveness of a human monoclonal anti-endotoxin antibody (HA-1A) in gram-negative sepsis: relationship to endotoxin and cytokine levels. *J Infect Dis* 1992; **166**(6): 1367-74.

11. McCloskey RV, Straube RC, Sanders C, Smith SM, Smith CR. Treatment of septic shock with human monoclonal antibody HA-1A. A randomized, double-blind, placebo-controlled trial. CHESS Trial Study Group. *Ann Intern Med* 1994; **121**(1): 1-5.

12. Ziegler EJ, Fisher CJ, Sprung CL, et al. Treatment of gram-negative bacteremia and septic shock with HA-1A human monoclonal antibody against endotoxin. A randomized, double-blind, placebo-controlled trial. The HA-1A Sepsis Study Group. *N Engl J Med* 1991; **324**(7): 429-36.

13. Greenberg RN, Wilson KM, Kunz AY, Wedel NI, Gorelick KJ. Observations using antiendotoxin antibody (E5) as adjuvant therapy in humans with suspected, serious, gram-negative sepsis. *Crit Care Med* 1992; **20**(6): 730-5.

14. Bigatello LM, Greene RE, Sprung CL, et al. HA-1A in septic patients with ARDS: results from the pivotal trial. *Intensive Care Medicine* 1994; **20**(5): 328-34.

15. Reinhart K, Glück T, Ligtenberg J, et al. CD14 receptor occupancy in severe sepsis: results of a phase I clinical trial with a recombinant chimeric CD14 monoclonal antibody (IC14). *Crit Care Med* 2004; **32**(5): 1100-8.

16. Hotchkiss RS, Colston E, Yende S, et al. Immune Checkpoint Inhibition in Sepsis: A Phase 1b Randomized, Placebo-Controlled, Single Ascending Dose Study of Antiprogrammed Cell Death-Ligand 1 Antibody (BMS-936559). *Crit Care Med* 2019; **47**(5): 632-42.

17. Albertson TE, Panacek EA, MacArthur RD, et al. Multicenter evaluation of a human monoclonal antibody to Enterobacteriaceae common antigen in patients with Gram-negative sepsis. *Crit Care Med* 2003; **31**(2): 419-27.

18. Laterre P-F, Pickkers P, Marx G, et al. Safety and tolerability of non-neutralizing adrenomedullin antibody adrecizumab (HAM8101) in septic shock patients: the AdrenOSS-2 phase 2a biomarker-guided trial. *Intensive Care Medicine* 2021; **47**(11): 1284-94.

19. Tugrul S, Ozcan PE, Akinci O, et al. The effects of IgM-enriched immunoglobulin preparations in patients with severe sepsis [ISRCTN28863830]. *Crit Care* 2002; **6**(4): 357-62.

20. Toth I, Mikor A, Leiner T, Molnar Z, Bogar L, Szakmany T. Effects of IgM-enriched immunoglobulin therapy in septic-shock-induced multiple organ failure: pilot study. *J Anesth* 2013; **27**(4): 618-22.

21. Rodríguez A, Rello J, Neira J, et al. Effects of high-dose of intravenous immunoglobulin and antibiotics on survival for severe sepsis undergoing surgery. *Shock* 2005; **23**(4): 298-304.

22. Hentrich M, Fehnle K, Ostermann H, et al. IgMA-enriched immunoglobulin in neutropenic patients with sepsis syndrome and septic shock: a randomized, controlled, multiple-center trial. *Crit Care Med* 2006; **34**(5): 1319-25.

23. Domizi R, Adrario E, Damiani E, et al. IgM-enriched immunoglobulins (Pentaglobin) may improve the microcirculation in sepsis: a pilot randomized trial. *Ann Intensive Care* 2019; **9**(1): 135.

24. De Simone C, Delogu G, Corbetta G. Intravenous immunoglobulins in association with antibiotics: a therapeutic trial in septic intensive care unit patients. *Crit Care Med* 1988; **16**(1): 23-6.

25. Brunner R, Rinner W, Haberler C, et al. Early treatment with IgM-enriched intravenous immunoglobulin does not mitigate critical illness polyneuropathy and/or myopathy in patients with multiple organ failure and SIRS/sepsis: a prospective, randomized, placebo-controlled, double-blinded trial. *Crit Care* 2013; **17**(5): R213.

26. Hall TC, Bilku DK, Neal CP, et al. The impact of an omega-3 fatty acid rich lipid emulsion on fatty acid profiles in critically ill septic patients. *Prostaglandins Leukot Essent Fatty Acids* 2016; **112**.

27. Galbán C, Montejo JC, Mesejo A, et al. An immune-enhancing enteral diet reduces mortality rate and episodes of bacteremia in septic intensive care unit patients. *Crit Care Med* 2000; **28**(3): 643-8.

28. Bower RH, Cerra FB, Bershadsky B, et al. Early enteral administration of a formula (Impact) supplemented with arginine, nucleotides, and fish oil in intensive care unit patients: results of a multicenter, prospective, randomized, clinical trial. *Crit Care Med* 1995; **23**(3): 436-49.

29. Burkhart CS, Dell-Kuster S, Siegemund M, et al. Effect of n-3 fatty acids on markers of brain injury and incidence of sepsis-associated delirium in septic patients. *Acta Anaesthesiol Scand* 2014; **58**(6): 689-700.

30. Chen H, Wang W, Hong Y, Zhang H, Hong C, Liu X. Single-blinded, randomized, and controlled clinical trial evaluating the effects of Omega-3 fatty acids among septic patients with intestinal dysfunction: A pilot study. *Exp Ther Med* 2017; **14**(2): 1505-11.

31. Hosny M, Nahas R, Ali S, Elshafei SA, Khaled H. Impact of oral omega-3 fatty acids supplementation in early sepsis on clinical outcome and immunomodulation. *The Egyptian Journal of Critical Care Medicine* 2013; **1**(3): 119-26.

32. Ibrahim ES. Enteral nutrition with omega-3 fatty acids in critically ill septic patients: A randomized double-blinded study. *Saudi J Anaesth* 2018; **12**(4): 529-34.

33. Pontes-Arruda A, Aragão AMA, Albuquerque JD. Effects of enteral feeding with eicosapentaenoic acid, gamma-linolenic acid, and antioxidants in mechanically ventilated patients with severe sepsis and septic shock. *Crit Care Med* 2006; **34**(9): 2325-33.

34. Barbosa VM, Miles EA, Calhau C, Lafuente E, Calder PC. Effects of a fish oil containing lipid emulsion on plasma phospholipid fatty acids, inflammatory markers, and clinical outcomes in septic patients: a randomized, controlled clinical trial. *Crit Care* 2010; **14**(1): R5.

35. Chen H, Wang W, Hong C, et al. Omega-3 Fish Oil Reduces Mortality Due to Severe Sepsis with Acute Gastrointestinal Injury Grade III. *Pharmacogn Mag* 2017; **13**(51): 407-12.

36. Bone RC, Fisher CJ, Clemmer TP, Slotman GJ, Metz CA, Balk RA. A controlled clinical trial of high-dose methylprednisolone in the treatment of severe sepsis and septic shock. *N Engl J Med* 1987; **317**(11): 653-8.

37. Briegel J, Möhnle P, Keh D, et al. Corticotropin-stimulated steroid profiles to predict shock development and mortality in sepsis: From the HYPRESS study. *Crit Care* 2022; **26**(1): 343.

38. Agarwal M, Dhar M, Agarwal D, Murlidharan A. Early Initiation of Low-Dose Hydrocortisone Therapy for Septic Shock in Geriatric Patients: A Randomized Control Trial. *J Assoc Physicians India* 2022; **70**(2): 11-2.

39. Lv Q-Q, Gu X-H, Chen Q-H, Yu J-Q, Zheng R-Q. Early initiation of low-dose hydrocortisone treatment for septic shock in adults: A randomized clinical trial. *Am J Emerg Med* 2017; **35**(12): 1810-4.

40. Annane D, Sébille V, Bellissant E. Effect of low doses of corticosteroids in septic shock patients with or without early acute respiratory distress syndrome. *Crit Care Med* 2006; **34**(1): 22-30.

41. Sevransky JE, Rothman RE, Hager DN, et al. Effect of Vitamin C, Thiamine, and Hydrocortisone on Ventilator- and Vasopressor-Free Days in Patients With Sepsis: The VICTAS Randomized Clinical Trial. *JAMA* 2021; **325**(8): 742-50.

42. Moskowitz A, Huang DT, Hou PC, et al. Effect of Ascorbic Acid, Corticosteroids, and Thiamine on Organ Injury in Septic Shock: The ACTS Randomized Clinical Trial. *JAMA* 2020; **324**(7): 642-50.

43. Arabi YM, Aljumah A, Dabbagh O, et al. Low-dose hydrocortisone in patients with cirrhosis and septic shock: a randomized controlled trial. *CMAJ* 2010; **182**(18): 1971-7.

44. Bollaert PE, Charpentier C, Levy B, Debouverie M, Audibert G, Larcan A. Reversal of late septic shock with supraphysiologic doses of hydrocortisone. *Crit Care Med* 1998; **26**(4): 645-50.

45. Birudaraju D, Hamal S, Tayek JA. Solumedrol Treatment for Severe Sepsis in Humans with a Blunted Adrenocorticotropic Hormone-Cortisol Response: A Prospective Randomized Double-Blind Placebo-Controlled Pilot Clinical Trial. *J Intensive Care Med* 2021; **37**(5): 693-7.

46. Annane D, Sébille V, Charpentier C, et al. Effect of treatment with low doses of hydrocortisone and fludrocortisone on mortality in patients with septic shock. *JAMA* 2002; **288**(7): 862-71.

47. Peduzzi P. Termination of the Department of Veterans Affairs Cooperative Study of steroid therapy for systemic sepsis. *Control Clin Trials* 1991; **12**(3): 395-407.

48. Russell JA, Walley KR, Gordon AC, et al. Interaction of vasopressin infusion, corticosteroid treatment, and mortality of septic shock. *Crit Care Med* 2009; **37**(3): 811-8.

49. Oppert M, Schindler R, Husung C, et al. Low-dose hydrocortisone improves shock reversal and reduces cytokine levels in early hyperdynamic septic shock. *Crit Care Med* 2005; **33**(11): 2457-64.

50. Thompson KJ, Taylor CB, Venkatesh B, et al. The cost-effectiveness of adjunctive corticosteroids for patients with septic shock. *Crit Care Resusc* 2020; **22**(3): 191-9.

51. Moreno R, Sprung CL, Annane D, et al. Time course of organ failure in patients with septic shock treated with hydrocortisone: results of the Corticus study. *Intensive Care Medicine* 2011; **37**(11): 1765-72.

52. Antcliffe DB, Burnham KL, Al-Beidh F, et al. Transcriptomic Signatures in Sepsis and a Differential Response to Steroids. From the VANISH Randomized Trial. *Am J Respir Crit Care Med* 2019; **199**(8): 980-6.

53. Mohanty R, Dillip Kumar Das, Sahu SK, Mallik B, Sahu RK. Effect of corticosteroid supplementation on adrenal suppression and survival in patients with sepsis. *Journal of Cardiovascular Disease Research* 2024; **15**(5): 310-8.

54. Wunderink R, Leeper K, Schein R, et al. Filgrastim in patients with pneumonia and severe sepsis or septic shock. *Chest* 2001; **119**(2): 523-9.

55. Root RK, Lodato RF, Patrick W, et al. Multicenter, double-blind, placebo-controlled study of the use of filgrastim in patients hospitalized with pneumonia and severe sepsis. *Crit Care Med* 2003; **31**(2): 367-73.

56. Stephens DP, Thomas JH, Higgins A, et al. Randomized, double-blind, placebo-controlled trial of granulocyte colony-stimulating factor in patients with septic shock. *Crit Care Med* 2008; **36**(2): 448-54.

57. Tanaka H, Nishino M, Nakamori Y, et al. Granulocyte colony-stimulating factor (G-CSF) stiffens leukocytes but attenuates inflammatory response without lung injury in septic patients. *J Trauma* 2001; **51**(6): 1110-6.

58. Meisel C, Schefold JC, Pschowski R, et al. Granulocyte-macrophage colony-stimulating factor to reverse sepsis-associated immunosuppression: a double-blind, randomized, placebo-controlled multicenter trial. *Am J Respir Crit Care Med* 2009; **180**(7): 640-8.

59. Francois B, Jeannet R, Daix T, et al. Interleukin-7 restores lymphocytes in septic shock: the IRIS-7 randomized clinical trial. *JCI Insight* 2018; **3**(5).

60. Daix T, Mathonnet A, Brakenridge S, et al. Intravenously administered interleukin-7 to reverse lymphopenia in patients with septic shock: a double-blind, randomized, placebo-controlled trial. *Ann Intensive Care* 2023; **13**(1): 17.

61. Zhou S, Gao H, Chen J, Liu W. The protective effect of ulinastatin in severe sepsis. a mechanistic approach. *FARMACIA* 2020; **68**(1).

62. Wu T, Zhang L, Kang C. The effect of ulinastatin on disbalance of inflammation and immune status in patients with severe sepsis. *Chin Crit Care Med* 2013; **25**(4).

63. He Y, Chen X, Zhang G, Guan L, Yu X. Clinical efficacy and safety of norepinephrine combined with ulinastatin in the treatment of septic shock. *Pak J Pharm Sci* 2022; **35**(2(Special)): 657-63.

64. Karnad DR, Bhadade R, Verma PK, et al. Intravenous administration of ulinastatin (human urinary trypsin inhibitor) in severe sepsis: a multicenter randomized controlled study. *Intensive Care Medicine* 2014; **40**(6): 830-8.

65. Guo M, Zhou B. Clinical efficacy of ulinastatin in the treatment of unliquefied pyogenic liver abscess complicated by septic shock: A randomized controlled trial. *Immun Inflamm Dis* 2023; **11**(4): e822.

66. Wu J, Zhou L, Liu J, et al. The efficacy of thymosin alpha 1 for severe sepsis (ETASS): a multicenter, single-blind, randomized and controlled trial. *Crit Care* 2013; **17**(1): R8.

67. Zhou L, Tan J, Li Y, et al. Immune and inflammation confusion in severe sepsis and effects of bi-immunomodulation therapy: a prospective, randomized, controlled clinical trial. *Natl Med J China* 2009; **89**(15): 1028-33.

68. Bai L, Qiu X, Ding X, et al. Value of Thymosin α1 Combined With Blood Purification to Increase Successful Rescues of Shock Patients. *Altern Ther Health Med* 2022; **28**(7): 146-52.

69. Chen J. Effects of thymosin-α1 on cell immunity function in patients with septic shock. *Chin Crit Care Med* 2007; **19**(3).

70. Wu J, Pei F, Zhou L, et al. The efficacy and safety of thymosin α1 for sepsis (TESTS): multicentre, double blinded, randomised, placebo controlled, phase 3 trial. *BMJ* 2025; **388**: e082583.

71. Huang S-w, Chen J, Ouyang B, Yang C-h, Chen M-y, Guan X-d. Immunotherapy improves immune homeostasis and increases survival rate of septic patients. *Chin J Traumatol* 2009; **12**(6): 344-9.

72. Su L, Meng F, Tang Y, et al. Clinical effects of ulinastatin and thymosin α1 on immune-modulation in septic patients. *Chin Crit Care Med* 2009; **21**(3).

73. Lin H, Guan X, Zhou L, Ai Y, Wang K. Clinical trial with a new immunomodulatory strategy: treatment of severe sepsis with Ulinastatin and Maipuxin. *Natl Med J China* 2007; **87**(07): 451-7.

74. Chen H, He M-y, Li Y-m. Treatment of patients with severe sepsis using ulinastatin and thymosin alpha1: a prospective, randomized, controlled pilot study. *Chin Med J (Engl)* 2009; **122**(8): 883-8.

75. Li Y, Chen H, Li X, et al. A New Immunomodulatory Therapy for Severe Sepsis: Ulinastatin Plus Thymosin α1. *Journal of Intensive Care Medicine* 2009; **24**(1).

76. Zhang Y, Chen H, Li Y-m, et al. Thymosin alpha1- and ulinastatin-based immunomodulatory strategy for sepsis arising from intra-abdominal infection due to carbapenem-resistant bacteria. *J Infect Dis* 2008; **198**(5): 723-30.

**Appendix 3: Inclusion criteria and exclusion criteria of participants in the includerandomized controlled trials**

| **id** | **Author,year** | **Inclusion Criteria** | **Exclusion Criteria** |
| --- | --- | --- | --- |
| 1 | Gallagher 2001 | • Age ≥18 years • Clinical diagnosis of sepsis • Within 24h: fever (≥38.0°C) or hypothermia (＜35.6°C), tachycardia (≥90 bpm), tachypnea (≥20 bpm or mechanical ventilation), and either: - Hypotension (SBP ≤90 mmHg or drop ≥40 mmHg) without antihypertensives/vasopressors, OR - Evidence of systemic toxicity/poor perfusion (≥2 of: metabolic acidosis, arterial hypoxia, elevated lactate, acute renal failure, coagulation abnormality, thrombocytopenia, acute mental status change, high cardiac index with low SVR) | • Investigational drug within 30 days • Previous murine monoclonal antibody • Steroids within 7 days • HIV positive • Sepsis due to major burns or organ transplant |
| 2 | Cohen 1996 | • Evidence of acute infection • Temperature ＞38.3°C or ＜35.6°C • Heart rate ＞90 bpm • Respiratory rate ＞20 bpm or mechanical ventilation • Evidence of acute organ dysfunction (e.g., altered mental status, hypoxemia, acidosis, oliguria, DIC) | • Age＜18 years • Pregnancy or nursing • Uncontrolled hemorrhage or burns ＞20% BSA • Neutrophil count ＜0.1×10⁹/L • Recent pentoxifylline (48h) or high-dose steroids (＞0.5 mg/kg/day prednisone equivalent) |
| 3 | Panacek 2004 | • Hospitalized, ≥18 years • Sepsis syndrome within 24h: - Evidence of acute infection - Temperature ≥38.6°C or ≤35.6°C - Heart rate ≥90 bpm - Respiratory rate ≥20 bpm or mechanical ventilation - Hypotension or evidence of end-organ dysfunction | • Age ＜18 years • Pregnancy • Weight ＞150 kg • Major burns ＞30% BSA • Allergy to murine proteins • Prior murine protein therapy • Recent corticosteroids (＞0.5 mg/kg/day hydrocortisone equivalent within 2 weeks) • Recent anticytokine therapy or pentoxifylline • DNR order • End-stage disease with life expectancy ＜2 months • WBC ＜500/μL |
| 4 | Dhainaut 1995 | • Septic shock within 12h of onset • Clinical evidence of infection • Temperature ≥38°C or ≤35.6°C • Heart rate ≥90 bpm • Respiratory rate ≥20 or mechanical ventilation • Hypotension requiring vasopressors after fluid resuscitation • Evidence of organ hypoperfusion (lactate ↑, PaO₂/FiO₂ ≤280, oliguria, altered mental status) | • Septic shock ＞12h duration • Moribund/terminal illness • Age ＜18 years • Pregnancy (if suspected) • Participation in another trial • Organ transplant • Major burns • Non-septic cardiogenic shock • Acute major hemorrhage • Prior monoclonal antibody use • Immunosuppressive therapy (including corticosteroids) |
| 5 | Abraham 1998 | • Adults ＞18 years • Septic shock present at randomization and within 12h before • Clinical evidence of acute infection • Temperature ＞38°C or ＜35°C • Heart rate ＞90 bpm, respiratory rate ＞20 or mechanical ventilation • Hypotension (SBP ＜90 mmHg for ＞30 min or drop ＞40 mmHg) refractory to ≥500 mL fluid • Evidence of organ dysfunction within 12h (altered mental status, hypoxemia, lactic acidosis, oliguria, coagulopathy) | • Previous enrollment in anti-TNF study • No informed consent • Life expectancy ＜2 months due to non-septic disease • Investigational drug within 30 days • Murine antibody exposure • Pentoxifylline within 48h • Chronic steroids (≥0.5 mg/kg/day for ＞3 days) • Allergy to human albumin • Pregnancy/lactation • Immunosuppression (cyclosporine, azathioprine, chemotherapy) • Granulocytes ＜1000/μL (unless sepsis-related) • Known hypersensitivity to mice • Uncontrolled hemorrhage requiring transfusion • Burns ＞20% TBSA • Weight ＞130 kg |
| 6 | Clark 1998 | • Severe sepsis (ACC/SCCM criteria) • Within 12h of onset • Age ≥18 years | • Age ＜18 years • Pregnancy • Expected death within 1 week from non-septic cause • Investigational drug within 30 days • HIV/AIDS • Major burns • Solid organ transplant • Uncontrolled hemorrhage • Chemotherapy-induced neutropenia • Hypersensitivity to human/murine antibodies |
| 7 | Abraham 1995 | • Adults ≥18 years • Sepsis syndrome: acute infection + temperature abnormality + tachycardia/tachypnea + evidence of organ dysfunction/hypoperfusion • Enrollment within 12 hours of organ dysfunction onset | • Expected survival ＜2 months due to underlying disease • Chronic vegetative state • Use of naloxone, investigational drugs, murine antibodies, high-dose steroids, pentoxifylline • Hypersensitivity to human albumin • Pregnancy/lactation • Granulocytopenia due to malignancy/chemotherapy • Uncontrolled hemorrhage • Burns ＞20% TBSA |
| 8 | Reinhart 2001 | - Adults ＞18 years - Sepsis with infection + temperature abnormality + cardiovascular/respiratory signs + organ dysfunction/hypoperfusion - IL-6 ＞1000 pg/mL (test kit positive) | - Prior murine protein exposure - Recent anticytokine therapy - Expected survival ＜2 months - High-dose steroids (＞0.5 mg/kg/day hydrocortisone equivalent) - Hypersensitivity to murine proteins or human albumin - Pregnancy/lactation - Immunosuppression - Burns ＞30% TBSA - DNR order - Weight＞100 kg |
| 9 | Angus 2000 | - Adults ≥18 years - Signs/symptoms of severe sepsis - Documented or probable gram-negative infection - Admitted to ICU - Not improving despite standard therapy and antibiotics - Enrollment within 12 hours of meeting criteria | - Granulocyte count ＜1×10⁹/L prior to sepsis - Burn-related infections - Pregnancy or lactation - Prior murine antibody therapy or allergy - HIV infection - Recent acute myocardial infarction - Refractory shock - Concurrent investigational therapy - No commitment to full life-support |
| 10 | Wortel 1992 | - Adults with suspected Gram-negative sepsis - Met sepsis criteria within 24h before enrollment - Participated in the larger HA-1A trial | Not explicitly listed in the excerpt |
| 11 | McCloskey 1994 | - Adults with septic shock within 6h before enrollment - Shock onset within 24h of enrollment - Presumptive Gram-negative infection as cause - Commitment to full supportive care | - Age ＜18 years - Pregnancy - Expected survival ＜3 months due to fatal underlying disease - Organ or bone marrow transplant within 6 months - Leukocyte count ＜500/mm³ - Burn injury ＞10% body surface within 2 months - Previous anti-endotoxin monoclonal antibody - DNR orders - Participation in other sepsis drug trials |
| 12 | Ziegler 1991 | - Adults with sepsis + presumed Gram-negative infection - Fever/hypothermia (＞38.3°C or ＜35.6°C) - Tachycardia (＞90 bpm) + tachypnea (＞20/min or mechanical ventilation) - Hypotension (SBP ≤90 mmHg or drop ≥40 mmHg) or ≥2 signs of systemic toxicity | - Age ＜18 years - Pregnancy - Rapidly fatal condition - Organ transplant recipient - Uncontrolled hemorrhage, cardiogenic shock, or burns as primary issue - Received monoclonal antibodies or IVIG (except FFP) within 21 days |
| 13 | Greenberg 1992 | - Adults ＞18 years - Clinical evidence of serious infection (e.g., sepsis, peritonitis, urosepsis, pneumonia) - Admitted to ICU for monitoring and intensive care - Moderate to severe illness | - Known hypersensitivity to animal serum - Serum creatinine ＞2.5 mg/dL (＞220 µmol/L) - Positive skin test to murine immunoglobulin |
| 14 | Bigatello 1994 | - Patients from multicenter sepsis trial with ARDS - PaO₂/FiO₂ ≤200 - Bilateral pulmonary opacities on chest radiograph - PAOP ≤18 mmHg or no clinical CHF - Study drug given within 24 hrs of meeting criteria | - Refractory hypotension - Lack of commitment to aggressive care - Irreversible underlying disease with rapid fatality expected - Unacceptable quality chest radiographs or missing PaO₂/FiO₂ data |
| 15 | Reinhart 2004 | - Adults with severe sepsis - ≥2 SIRS criteria due to infection - Sustained hypotension or organ hypoperfusion - Study drug within 24 hrs of meeting criteria | - Refractory hypotension despite vasopressors - Lack of commitment to aggressive care - Irreversible rapidly fatal underlying disease - Pregnancy, lactation, or other protocol-specific contraindications |
| 16 | Hotchkiss 2019 | • Age ≥18 years • Sepsis with organ dysfunction (hypotension, acute respiratory failure, or AKI) within 24h • Absolute lymphocyte count ≤1100 cells/μL within 96h • ICU admission | • Advanced directive limiting care • Active autoimmune disease • History of transplantation • Cancer diagnosis/treatment in past 6 months • Previous sepsis with ICU admission during same hospitalization |
| 17 | Albertson 2003 | • Age ≥18 years • Clinical diagnosis of sepsis with shock or MODS • Presumptive evidence of Gram-negative infection (Gram stain or culture within 72h) • SIRS criteria (temperature, HR, RR, WBC) + organ dysfunction | • Age ＜18 years • Pregnancy • Weight ＞150 kg • Irreversible non-septic fatal illness • Lung transplant, burns, prior monoclonal antibody therapy • Cardiogenic shock, uncontrolled hemorrhage |
| 18 | Laterre 2021 | • Age ≥18 years • Early septic shock (start of vasopressor therapy ＜12h before inclusion) • Bio-ADM ＞70 pg/mL | • Moribund state • Severe chronic liver disease (Child-Pugh C) • Other protocol-specific exclusions (not fully detailed in abstract) |
| 19 | Tugrul 2002 | Severe sepsis (temperature ＞38°C or ＜36°C, HR ＞90/min, RR ＞20/min or PaCO₂ ＜32 mmHg, WBC ＞12,000 or ＜4,000/mm³, documented infection, organ dysfunction or hypotension) | Not explicitly listed in the provided text |
| 20 | Toth 2013 | - Early septic shock (＜24h from diagnosis) - Reversible with inotropic/vasopressor support - Severe respiratory failure (PaO₂/FiO₂ ＜225 mmHg on mechanical ventilation) | - Chronic cardiovascular, respiratory, renal (on RRT), or liver failure - Expected survival ＜24h |
| 21 | Rodríguez 2005 | - Severe sepsis or septic shock of intra-abdominal origin - Admitted to ICU within 24h of symptom onset - Surgically confirmed abdominal focus - Purulent material or Gram stain confirmation | - Severe immunosuppression - Irreversible end-stage organ damage - GCS = 3 - Severe heart failure (NYHA IV) - Pregnancy |
| 22 | Hentrich 2006 | - Age ＞18 years - Diagnosis of acute leukemia, high-grade NHL, or other high-grade hematologic malignancies - Neutropenia (granulocytes ＜1000/μL) - Sepsis syndrome or septic shock - Written informed consent | - Age ＜18 years - Pregnancy or lactation - Known allergy to study medication - HIV infection - IVIg within past 4 weeks - Refusal of informed consent |
| 23 | Domizi 2019 | - Adult patients (≥18 years) - Diagnosis of sepsis or septic shock (based on 2001 criteria) - Enrollment within 24 h of sepsis onset | - Contraindications to immunoglobulin - Sepsis ＞24 h before enrollment - Chronic renal failure - Life expectancy ＜24 h - Pregnancy - Oral surgery or maxillofacial trauma impeding sublingual microcirculation assessment - Participation in other interventional studies |
| 24 | De Simone 1988 | - ICU patients with severe sepsis - Hospitalized in ICU between Jan 1984–Mar 1985 - Clinical and laboratory evidence of sepsis | Not explicitly listed in the provided text |
| 25 | Brunner 2013 | • Age 18–80 years • Multiple organ failure (≥2 organs) • SIRS/Sepsis diagnosis • Early clinical signs of CIPNM (decreased reflexes, muscle weakness, etc.) | • Age ＜18 or ＞80 years • Body weight ＞135 kg • Pregnancy or breastfeeding • Known IgA deficiency • IVIG intolerance • Pre-existing neuromuscular or CNS disorders • Severe pulmonary edema due to heart failure • Expected survival ＜28 days • Moribund state • HIV with CD4+ ＜50/mm³ • Chronic ventilator support for non-respiratory reasons |
| 26 | Hall 2016 | • Adult patients admitted to ICU/HDU • Sepsis (suspected or proven infection + ≥2 SIRS criteria) • Enrolled within 12 hours of sepsis diagnosis | Not explicitly listed in the provided text |
| 27 | Galbán 2000 | • Age ＞14 years • Sepsis (confirmed or suspected infection + ≥2 SIRS criteria) • APACHE II score ≥10 • Admitted to ICU and requiring enteral nutrition | • Previous radiotherapy • Immunosuppressive therapy • AIDS • Neoplasia or metastases • Prior immunonutrition (e.g., arginine, ω-3, nucleotides) |
| 28 | Bower 1995 | • Age 18–80 years • Admitted to ICU after trauma, surgery, or sepsis • APACHE II score ≥10 or TISS score ≥20 • Expected to receive enteral nutrition for ≥7 days • Enrollment within 48 hrs of qualifying event | • Immunosuppressive therapy (past 6 months) • Immunosuppressive conditions (e.g., AIDS) • Autoimmune disorders • Organ transplantation • Recent chemotherapy/radiation • Active cancer with residual tumor • Type 1 diabetes • Morbid obesity (＞200% IBW or BMI ＞40) • Isolated severe head injury (GCS ＜5) • COPD with Pco₂ ＞45 torr • NYHA class III/IV cardiac disease • Renal disease requiring dialysis or creatinine ＞2.5 mg/dL • Liver cirrhosis or bilirubin ＞3.0 mg/dL |
| 29 | Burkhart 2014 | • Age ≥18 years • Sepsis, severe sepsis, or septic shock (per 2001 SCCM/ESICM/ACCP/ATS/SIS criteria) • Admitted to ICU within 24 hrs | • CNS infection • Brain trauma • Coagulopathy (INR ＞2.4, platelets ＜50×10⁹/L, active bleeding) • History of cerebrovascular disease • CNS neoplasia • Long-term psychiatric medication • Allergy to fish or egg protein |
| 30 | CHEN 2017-01 | • Age ≥18 years • SIRS due to infection/trauma • Marshall score ＞3 • Intestinal dysfunction + unable to tolerate enteral feeding • Treated with carbapenems • ICU stay ≥7 days | • Marshall score ≥20 • Life expectancy ＜28 days due to chronic/incurable disease • Life expectancy ＜24 hrs • ICU stay ＜7 days • Signed DNR order |
| 31 | Hosny 2013 | - Age ≥ 18 years - Diagnosis of early sepsis (within 24h of meeting sepsis criteria) - Signed informed consent | - Age ＜ 18 years - Significant immunologic suppression (WBC ＜5000/mm³) - Imminence of receiving parenteral nutrition - Uncontrolled diarrhea - Recent GI bleeding - End-stage liver or renal disease - Life expectancy ＜24h - Life expectancy ＜28 days due to chronic/incurable disease - Pregnancy - Allergy to omega-3 FA - ＞16h after meeting inclusion criteria - Recent omega-3 use - Severe sepsis, MODS, or septic shock on admission - APACHE II ＞25 |
| 32 | Ibrahim 2018 | - Age ＞18 years - Diagnosis of sepsis (clinical + lab criteria) - Able to receive enteral nutrition - Informed consent from relatives | - End-stage liver or renal disease - Hemodynamic instability - Immunosuppression - Steroid use - GI comorbidity - Allergy to omega-3 - Omega-3 use within 1 week before ICU admission - Expected survival ＜ 24h - Mechanically ventilated on ICU admission |
| 33 | Pontes-Arruda 2006 | - Age ＞18 years - Mechanical ventilation with PaO₂/FiO₂ ＜200 - Enteral access - Diagnosis of severe sepsis or septic shock | - Pregnancy or breastfeeding - Age ＜18 years - Life expectancy ＜ 28 days - Chronic renal insufficiency - Acute pancreatitis of unknown origin - Participation in another trial within 30 days - Head trauma with GCS ≤ 5 - Recent stroke or SAH - Immunosuppression (WBC＜5000/mm³) - HIV infection - No indication for enteral nutrition or imminent parenteral nutrition - Uncontrolled diarrhea - Recent GI bleeding - Planned extubation before study day 4 - Physician decision to exclude |
| 34 | Barbosa 2010 | - Adult patients with SIRS or sepsis - Expected to require PN for ≥5 days - Admitted to medical ICU | Did not start PN |
| 35 | CHEN 2017-02 | 1. Age ≥ 18 years 2. Severe SIRS due to severe infection, requiring mechanical ventilation 3. Marshall score ＞ 3 4. AGI Grade III (abdominal infection, abdominal or intestinal surgery) 5. Severe infection requiring carbapenems after ICU admission | 1. Marshall score ≥ 20 2. Life expectancy ＜ 28 days due to chronic/incurable disease 3. Life expectancy ＜ 24 hours or discharge 4. ICU stay ＜ 7 days after inclusion 5. Do Not Resuscitate order 6. Hematological or rheumatic disease 7. Severe liver dysfunction (Child-Pugh ＞10 or Grade C) |
| 36 | Bone 1987 | - Clinical suspicion of infection - Fever/hypothermia (＞38.3°C or ＜35.6°C) - Tachypnea (＞20/min), tachycardia (＞90/min) - One organ dysfunction: altered mental status, hypoxemia, elevated lactate, oliguria - Shock ≤2 hours before enrollment (if present) | - Shock ＞2 hours before enrollment - Use of corticosteroids or other experimental treatments |
| 37 | Briegel 2022 | - Adults with severe sepsis but not in shock - Participating in HYPRESS trial - Corticotropin test performed before randomization | - Received etomidate or steroids before enrollment - Corticotropin test failure (no change in analytes) |
| 38 | Agarwal 2021 | 1. Age ＞ 60 years 2. Admitted to ICU with septic shock or developed septic shock during hospital stay | 1. Not meeting septic shock criteria 2. Systemic corticosteroid therapy within 3 months before septic shock 3. Refusal of consent |
| 39 | Lv 2017 | 1. Age ≥ 18 years 2. Onset of septic shock within 6 hours | 1. Systemic corticosteroid therapy within 3 months before septic shock 2. High-dose steroid therapy 3. Immunosuppression 4. Refusal by attending staff or family |
| 40 | Annane 2006 | - Documented or suspected infection - Temperature ＞38.3°C or ＜35.6°C - Heart rate ＞90 bpm - Systolic arterial pressure ＜90 mmHg for ≥1 hr despite fluids and dopamine ＞5 µg/kg/min or epinephrine/norepinephrine - Urine output ＜0.5 mL/kg for ≥1 hr or PaO₂/FiO₂ ＜280 mmHg - Arterial lactate ＞2 mmol/L - Mechanical ventilation - ARDS criteria: bilateral infiltrates, PaO₂/  FiO₂ ＜200 mmHg, PAOP ≤18 mmHg or no left atrial hypertension | Not explicitly listed in the provided excerpt |
| 41 | Sevransky 2021 | - Age ≥18 years - Suspected infection with planned ICU admission - Respiratory dysfunction: PaO₂/FiO₂ ≤300 or SpO₂/FiO₂ ≤315, and need for: - Intubation & mechanical ventilation - Noninvasive positive pressure ventilation - High-flow nasal cannula ≥40 L/min with FiO₂ ≥0.40 - Cardiovascular dysfunction: Vasopressor ＞1 hr to maintain MAP ≥65 mmHg after ≥1 L IV crystalloid | - Need for organ support for non-sepsis diagnosis - Home oxygen use - Limitations in care - Patient refusal - Other administrative or clinical reasons |
| 42 | Moskowitz 2020 | - Age ≥18 years - Suspected or confirmed infection - Receiving vasopressor for sepsis | - Allergy to study drug components - Clinical indication for any study drug - Symptomatic kidney stones in past year - G6PD deficiency or hemochromatosis - Receiving kidney replacement therapy - Not expected to survive 24 hours - Pregnancy, imprisonment - Other (steroid contraindication, supplemental thiamine use, etc.) |
| 43 | Arabi 2010 | • Age ≥ 18 years • Liver cirrhosis • Septic shock (within 72 hours of hypotension onset) | Not explicitly listed in the excerpt |
| 44 | Bollaert 1998 | • Age ＞18 years • Septic shock requiring catecholamines for ＞48 hours • Mechanical ventilation and signs of infection + organ dysfunction | • Underlying fatal disease with life expectancy ＜1 week • Recent gastroduodenal ulcer, GI bleeding, or corticosteroid treatment • Absolute adrenal insufficiency (post-ACTH cortisol ＜18 µg/dL) |
| 45 | Birudaraju 2022 | • Age ＞18 years • Severe sepsis or septic shock • Blunted cortisol response to ACTH (delta cortisol ＜13 µg/dL) | • HIV • Pregnancy • Do not resuscitate (DNR) status • Chronic steroid use • Adrenal insufficiency (all cortisol values ＜20 µg/dL before and after ACTH test) |
| 46 | Annane 2002 | • Adult patients (≥18 years) • Septic shock, defined by: 1. Documented or suspected site of infection 2. Temperature ＞38.3°C or ＜35.6°C 3. Heart rate ＞90 bpm 4. Systolic arterial pressure ＜90 mmHg for ≥1 hour despite fluid resuscitation and ＞5 µg/kg/min dopamine or treatment with epinephrine/norepinephrine 5. Urine output ＜0.5 mL/kg/h for ≥1 hour or PaO₂/FiO₂ ＜280 mm Hg 6. Arterial lactate ＞2 mmol/L 7. Need for mechanical ventilation • Randomized within 3 hours (later extended to 8 hours) of shock onset • Short corticotropin test performed • Written informed consent obtained | • Pregnancy • Acute myocardial infarction or pulmonary embolism • Advanced cancer or AIDS • Contraindication or formal indication for corticosteroids • Received etomidate within 6 hours prior to randomization |
| 47 | Peduzzi 1991 | • Clinical suspicion of sepsis • Development of at least 4 of the following 7 signs within an 8-hour period: 1. Fever (＞102°F) or hypothermia (＜96°F) 2. Tachypnea (＞28 breaths/min) or hypocapnia (PaCO₂ ＜32 mmHg) 3. Tachycardia (heart rate ＞100 bpm) 4. Hypotension (systolic BP ＜90 mm Hg) 5. Abnormal WBC count (＜3500/mm³ or ≥15,000/mm³) or abnormal neutrophil count 6. Thrombocytopenia (＜100,000 platelets/mm³) 7. Surgical/invasive procedure in prior 48 hours or obvious primary septic site | Inability to give consent due to altered sensorium |
| 48 | Russell 2009 | • Age ≥16 years • Septic shock unresponsive to fluids • Requiring at least 5 µg/min of norepinephrine (or equivalent) for 6 hours • Meeting all of the following: 1. ≥2 Systemic Inflammatory Response Syndrome (SIRS) criteria 2. Proven or suspected infection 3. Hypotension requiring vasopressors 4. At least one new organ dysfunction | Not explicitly listed in the substudy paper |
| 49 | Oppert 2005 | - Adult patients with early hyperdynamic septic shock - Two or more SIRS criteria (tachycardia ＞90 bpm, temp ≥38.5°C or ＜36°C, leukocytosis ≥12,000/nL or ＞10% immature cells, RR ＞20/min or mechanical ventilation) - Evidence or suspicion of infection - SBP ＜90 mmHg for ≥1 hr despite fluid resuscitation (CVP ≥10 mmHg or PAOP ≥15 mmHg) - Cardiac index ≥3.5 L/min/m² - Need for vasopressor support - Inclusion within 24 hrs of shock onset | - Pregnancy - HIV positive - Organ transplant recipients - Contraindication or formal indication for steroids - Glucocorticoid use (including inhaled) within 4 weeks prior |
| 50 | Thompson 2020 | - Mechanically ventilated adult patients with septic shock - Enrolled in ADRENAL trial in NSW or Queensland, Australia - Consent for data linkage and follow-up | - Not specified in the extracted text beyond ADRENAL trial exclusions (e.g., life expectancy ＜24 hrs, long-term corticosteroid use, etc.) |
| 51 | Moreno 2011 | - Age ≥18 years - Clinical evidence of infection - Systemic response to infection - Shock within previous 72 hrs (SBP ＜90 mmHg despite fluid or need for vasopressors ≥1 hr) - Hypoperfusion or organ dysfunction due to sepsis - Informed consent | - Underlying disease with poor prognosis - Immunosuppression - Prior corticosteroid administration |
| 52 | Antcliffe 2018 | ·Diagnosis of sepsis with persistent hypotension requiring vasopressors after adequate fluid resuscitation. ·Meeting 2/4 SIRS criteria with a suspected or confirmed infection within 24 hours. | 1.Prior continuous vasopressor infusion during this ICU admission (excluding emergency use for ＜6 hours for stabilization)； 2.Regular systemic corticosteroid therapy within the previous 3 months (excluding inhaled corticosteroids)； 3.Known adrenal insufficiency； 4.End-stage renal disease requiring long-term dialysis； 5.Lack of commitment to full active care by the treating team； 6.Pregnancy； 7.Known acute mesenteric ischemia； 8.History of Raynaud's phenomenon, systemic sclerosis, or other vasospastic diseases； 9.Current enrollment in another interventional trial or recent participation (within 30 days) in an investigational drug study that may interact with the study protocol； 10.History of anaphylaxis or hypersensitivity to any study drug. |
| 53 | Mohanty 2024 | 1. Evidence of sepsis (≥2 SIRS criteria + infection) 2. Both sexes 3. Serum albumin ＞2.5 g/dL | 1. Known HPA axis disease 2. Current glucocorticoid use 3. Multi-organ dysfunction syndrome (MODS) 4. Established septic shock on inotropic support |
| 54 | Wunderink 2001 | • Men and women ≥18 years • Hospitalized in ICU • Diagnosis of pneumonia within 72 hrs before randomization • Diagnosis of severe sepsis or septic shock within 24 hrs before randomization • Pneumonia criteria: fever (≥38°C) or hypothermia (≤35.5°C), tachycardia (≥90 beats/min), new/changing radiographic infiltrate, need for mechanical ventilation • Microbiological evidence: Gram's stain of sputum/tracheal aspirate (＜10 epithelial cells and ＞25 WBCs per low-power field), or BAL/protected-specimen brush/pleural fluid with pathogen morphology, or positive culture (blood, pleural fluid, or bronchoscopy specimen with threshold: BAL ≥10⁴ cfu/mL or protected-specimen brush ≥10³ cfu/mL) | • Irreversible disease (other than sepsis) with rapidly fatal course • Burns as primary injury • Cardiogenic shock as primary acute condition • Uncontrolled hemorrhage • Myeloid malignancies • Bone marrow transplant within past year • WBC count ＜1.0 × 10⁹/L or ＞40 × 10⁹/L |
| 55 | root 2003 | • Adult patients (＞18 years old) • Bacterial pneumonia (community-acquired or nosocomial) confirmed by chest radiograph and positive culture or Gram-negative stain • Severe sepsis (sepsis-induced hypotension or organ dysfunction) | • Pregnant or breastfeeding • Life expectancy ＜72 hrs unrelated to acute infection • Cardiogenic shock as primary acute condition • Uncontrolled hemorrhage • Full-thickness thermal or chemical burns (＞20% body surface) • White blood cell count ＞40 × 10⁹/L • History of NYHA class IV heart failure • Do-not-resuscitate orders • Known hypersensitivity to E. coli-derived products |
| 56 | stephens 2008 | • Adult patients (＞18 yrs) • Admitted to ICU • Met criteria for septic shock (ACCP/SCCM consensus) • Assessed for eligibility within 24 hrs of meeting criteria • Time from screening to consent and drug administration ≤36 hrs | • Culture-confirmed melioidosis • Hematologic malignancy • Febrile neutropenia • Myelodysplasia or congenital neutropenia • Splenomegaly • Acute myocardial infarction in previous 24 hrs • Pregnancy • Known hypersensitivity to G-CSF • Known objection to participation • Previous transplantation • Active orders limiting treatment • Expected survival ＜24 hrs • Previously enrolled or received G-CSF within previous month |
| 57 | Tanaka 2001 | - Septic patients with relative neutropenia (peripheral total leukocyte count ＜8,000/mm³) - Serum C-reactive protein concentration ＞10 mg/dL - Diagnosed with sepsis according to ACCP/SCCM Consensus Conference criteria | - Age ＜15 years - Malignant tumor - Intestinal pneumonia (likely a typo for "interstitial pneumonia") |
| 58 | Meisel 2009 | - Patients with severe sepsis or septic shock - Sepsis-induced immunosuppression (defined as monocytic HLA-DR ＜8,000 monoclonal antibodies per cell for 2 consecutive days) | 1.Pregnancy.  2.Known hypersensitivity to GM-CSF, mannitol, citrate, or NaHCO3⎯  3.Systemic autoimmune disease, hematologic disease (neoplasma, acute leukemia), transplant patients, or patients on steroid medication receiving a predisolon equivalent of ＞10 mg per day  4.Human immunodeficiency virus positivity  5.Presence of an advanced directive to withhold or to withdraw life sustaining treatment  6.Underlying disease with a prognosis for survival ＜ 3 months, or moribund patient highly likely to die within 24 hours  7. Cardiopulmonary resuscitation (＜ 72 hrs.) before enrolment  8.Acute myocardial infarction or pulmonary embolisation (＜ 72 hrs.)  9.Participation in a clinical trial until 30 days prior to inclusion |
| 59 | Francois 2018 | • Age 18-80 years • ≥2 criteria for systemic inflammatory response syndrome (SIRS) • Clinically or microbiologically suspected infection • SOFA score ≥2 at 48-120 hours after ICU admission • Require vasopressor treatment • Absolute lymphocyte count ≤900 cells/μl within 24 hours of informed consent | • Current chemotherapy or radiotherapy for cancer • Current or history of hematologic malignancy or lymphoma • Cardiopulmonary resuscitation within 4 weeks • History or current evidence of autoimmune disorder • Organ transplant recipient • HIV/AIDS, hepatitis B or C • Corticosteroids ≥300 mg/day hydrocortisone equivalent • Biologics that block cytokines |
| 60 | Daix 2023 | • Age 18-85 years • Meet Sepsis-2 criteria • Vasopressor-dependent septic shock (hypotension requiring vasopressor for ≥6 h to maintain SBP ≥90 mmHg or MAP ≥65 mmHg) • Acute respiratory failure requiring mechanical ventilation and/or acute kidney injury (creatinine >2.0 mg/dL or urine output ＜0.5 mL/kg/h for ＞4 h despite fluid resuscitation) • Persistent lymphopenia: two absolute lymphocyte counts (ALCs) ≤900 cells/mm³ at least 12 h apart within 48 h after sepsis diagnosis | • Evidence of autoimmune disorders • Active hematological diseases • Cancer with current chemotherapy or radiation therapy • Treatment with corticosteroids equivalent to ≥300 mg/day hydrocortisone • Treatment with immunosuppressive medications |
| 61 | Zhou 2020 | - Patients meeting SIRS diagnostic criteria (≥2 of: temperature ＞38°C or ＜36°C, HR ＞90 bpm, RR ＞20 bpm or PaCO₂ ＜32 mmHg, WBC ＞12×10⁹/L or ＜4×10⁹/L or immature granulocytes ＞10%). - Suspected or definite infection focus. - Severe sepsis or septic shock (hypotension, lactic acid above normal, urine output ＜0.5 mL/kg/h for ≥2 h after fluid resuscitation, acute lung injury with PaO₂/FiO₂ ≤250 or ≤200 if pneumonia, creatinine ＞2.0 mg/dL, platelets ＜100,000/μL, INR ＞1.5). | - History of diabetes mellitus. - Age ＜18 years. - Expected hospital stay ≤24 h. - Chemotherapy within past 2 weeks. - Pregnant or lactating women. |
| 62 | Tiejun 2013 | 1.admission to the general intensive care unit (ICU) of our hospital with a diagnosis of severe sepsis between October 2011 and October 2012; 2.fulfillment of the severe sepsis criteria defined by the 2001 International Sepsis Definitions Conference. | 1.Age＜18 years. 2.Presence of autoimmune diseases. 3.Acute cerebrovascular disease. 4.Acute myocardial infarction. 5.Viral hepatitis. 6.Use of corticosteroids or immunosuppressants within 3 months prior to admission. 7.Failure to complete the immunomodulatory therapy protocol (due to death or discharge). |
| 63 | He 2022 | - Patients meeting diagnostic criteria for septic shock (2016 International Guideline): persistent hypotension, RR ≥22/min, GCS ≤13, SBP ≤100 mmHg. - Complete and authentic medical records. - No allergy to study drugs. - Informed consent provided. | - Other infectious diseases. - Schizophrenia, inability to communicate, or refusal to cooperate. - Complicated immune function disease or prior similar treatment. - Unable to participate in the entire study for uncontrollable reasons. |
| 64 | Karnad 2014 | - Adults aged 18-60 years with severe sepsis admitted to ICU. - Evidence of infection and ≥3 SIRS criteria. - Dysfunction of at least one organ/system (cardiovascular, renal, respiratory, hematologic) of ≤48 h duration. | - Pregnant or breastfeeding women. - Platelet count ＜30,000/mm³. - History of organ transplantation. - Poorly controlled neoplasm. - End-stage chronic kidney or liver disease. - Weight ＞135 kg. - Limitation of care planned or expected to die within 24 h. |
| 65 | Guo 2023 | Unliquefied PLA: - Symptoms of digestive system infection (chills, high fever, pain in liver region). - Unliquefied PLA confirmed by ultrasound, CT, MRI. - WBC, CRP, PCT, blood culture confirming infectious lesions. - If no clear pathogenic evidence, anti-infection treatment led to symptom control and lesion resolution. Septic Shock (Sepsis-3): - Persistent hypotension despite fluid resuscitation, requiring vasoactive agents to maintain MAP ＞65 mmHg. - Blood lactate ＞2 mmol/L. - SOFA score ≥2 from 48 h before to 24 h after infection. | - LA caused by amoebae, Mycobacterium tuberculosis, or fungi. - PLA that has liquefied or requires puncture/surgery. - Age ＜18 or ＞80 years. - Pregnancy or lactation. - NYHA Class IV heart failure, non-infectious cardiogenic shock, uncontrolled hemorrhagic shock. - Severe pre-existing liver disease (portal hypertension, Child-Pugh C cirrhosis, acute liver failure). - Prior solid organ or bone marrow transplantation. - Severe pulmonary fibrosis or noninvasive ventilation prior to onset. - Myocardial infarction within 3 months prior. - Cardiopulmonary resuscitation within 72 h prior. - Invasive fungal infection or active tuberculosis. - Third-degree burns ≥30% body surface. - Immunosuppression due to drugs/disease (e.g., AIDS). - Unremitting hematologic/lymphatic tumors. - History of UTI allergy. - Prior participation in clinical trials. - Inability to obtain informed consent. - Expected survival ＜2 months or vegetative state. - Patients declining comprehensive, aggressive treatment. |
| 66 | Jianfeng 2013 | • known or suspected infection based on clinical data at the time of screening • two or more signs of systemic inflammation • sepsis-induced dysfunction of at least one organ or system | 1. Pregnant or lactation period. 2. Age ＜18 yrs or ＞85 yrs 3. Receiving immunosuppressive therapy such as cyclosporine, azathioprine or cancer chemotherapy within one month.  4. History of bone marrow, lung, liver, kidney, pancreas or small bowel transplantation; 5. Acute pancreatitis with no established source of infection. 6. Not expected to survive 28 days because of end-stage diseases. 7. Participation in another clinical trial. |
| 67 | Zhou 2009 | - Severe sepsis (sepsis with organ dysfunction) - Age ＞18 years - Severe trauma (after 3 days) - Marshall score ＞ 5 | - Use of other drugs that affect immune function - Chronic liver or kidney failure - Advanced malignant tumor - Post-randomization exclusion: death or discharge within 24 hours; failure to adhere to systematic treatment |
| 68 | Bai 2022 | • Age ＞18 years • Met the diagnostic criteria for Septic Shock (SS) • Acute Physiology and Chronic Health Enquiry II (APACHE II) score ＞12 • Complete medical records available at the hospital • Agreed to voluntarily participate in the study | • Autoimmune deficiency disease, blood disease, malignant tumor, or mental diseases; • Recently used immunosuppressants, hormones, or other immune stimulants; • Pregnant or lactating; • Drug contraindications; • Referrals; • Death during treatment (if the patient died before the treatment was completed we cannot confirm whether T-α1 plays a role in the end, so such patients are excluded). |
| 69 | Chen 2007 | - Septic shock meeting 2001 International Sepsis Definitions Conference criteria - APACHE II score between 15 and 25 | - Use of immunosuppressive drugs or hormones in the past 3 months - Tumor, organ transplantation, chronic end-stage diseases |
| 70 | Wu 2025 | 1. Age 18-85 years 2. Diagnosis of sepsis (Sepsis-3 criteria) | 1. Pregnancy or lactation 2. Hematological malignancies 3. Organ or bone marrow transplant 4. Acute autoimmune disease or glomerulonephritis 5. Allergy to thymosin α1 6. CPR within 72 hrs with GCS ≤8 7. Recent immunosuppression or steroids ＞10 mg/day prednisolone equivalent 8. Recent participation in immunology trials 9. Undrained infection focus 10. Expected death within 28 days 11. End-of-life care decision |
| 71 | Huang 2009 | - Diagnosis of sepsis according to ACCP/SCCM Consensus Conference criteria. - SIRS with documented infection (positive culture). - SIRS criteria: ≥2 of: temperature ＞38°C or ＜36°C; heart rate ＞90 beats/min; respiratory rate ＞20 breaths/min; WBC ＞12,000 or ＜4,000 cells/mm³ or immature cells ＞10%. | - Age ＜18 or ＞80 years. - Incurable malignancies with metastases. - Long-term high-dose immunosuppressive drugs or NSAIDs within previous 2 days. - Acute myocardial infarction. - Chronic compensated organ dysfunction (e.g., dialysis-dependent renal failure, moderate to severe chronic heart failure). |
| 72 | Su 2009 | Sepsis meeting 2004 sepsis diagnostic criteria with SIRS according to 1992 ACCP/SCCM criteria | - Use of immunosuppressive drugs (e.g., hormones) - Tumor and organ transplantation - Incomplete treatment (combined use of drugs less than 10 days) - Withdrawn during treatment - Age ＜ 18 years |
| 73 | Lin 2007 | - Age ≥ 18 years - Meet the diagnostic criteria for sepsis (SIRS + evidence or suspected evidence of infection) - Marshall score between 5 and 20 | Patients with poor prognosis of underlying diseases that may become the main cause of death in the short term (e.g., severe traumatic brain injury, post-cardiopulmonary resuscitation, advanced malignant tumors) |
| 74 | Chen 2009 | - Diagnosis of severe sepsis fulfilling criteria of the 2001 International Sepsis Definitions Conference. - Informed consent obtained. | - Age ＜18 or ＞80 years. - Incurable malignancies with documented metastases. - Chronic treatment with high-dose immunosuppressive drugs or high-dose NSAIDs within previous 2 days. - Acute myocardial infarction. - Chronic compensated organ dysfunction (e.g., chronic liver disease, dialysis-dependent renal failure, moderate to severe chronic heart failure |
| 75 | Li 2009 | - Patients with a diagnosis of sepsis fulfilling the criteria of the Consensus Conference on Sepsis and related syndromes. - Informed consent obtained. | Not explicitly listed in the excerpt |
| 76 | Zhang 2008 | 1. Diagnosis of sepsis (≥2 SIRS criteria + documented infection) 2. Confirmed infection with carbapenem-resistant bacteria (by culture and susceptibility testing) 3. Admitted to ICU | 1. Age ＜18 or ＞80 years 2. Incurable malignancies with metastases 3. Long-term high-dose immunosuppressive therapy or high-dose NSAIDs within previous 2 days 4. Acute myocardial infarction 5. Chronic compensated organ dysfunction (e.g., chronic liver disease, dialysis-dependent renal failure, moderate to severe chronic heart failure) |

Abbreviations: SBP＝Systolic Blood Pressure; SVR＝Systemic Vascular Resistance; HIV＝Human Immunodeficiency Virus; DIC＝Disseminated Intravascular Coagulation; BSA＝Body Surface Area; DNR＝Do Not Resuscitate; WBC＝White Blood Cell; IVIG＝Intravenous Immunoglobulin; FFP＝Fresh Frozen Plasma; ARDS＝Acute Respiratory Distress Syndrome; PAOP＝Pulmonary Artery Occlusion Pressure; CHF＝Chronic Heart Failure; SIRS＝Systemic Inflammatory Response Syndrome; AKI＝Acute Kidney Injury; MODS＝Multiple Organ Dysfunction Syndrome; ICU = Intensive Care Unit; GCS = Glasgow Coma Scale; NYHA = New York Heart Association; CVP = Central Venous Pressure; RRT = Renal Replacement Therapy; NHL = Non-Hodgkin Lymphoma; CIPNM = Critical Illness Polyneuropathy and Myopathy; CNS = Central Nervous System; HDU = High Dependency Unit; APACHE = Acute Physiology and Chronic Health Evaluation; TISS = Therapeutic Intervention Scoring System; IBW = Ideal Body Weight; BMI = Body Mass Index; COPD = Chronic Obstructive Pulmonary Disease; INR = International Normalized Ratio; PaO₂/FiO₂ = Partial Pressure of Arterial Oxygen / Fraction of Inspired Oxygen; LA = Liver Abscess; PLA = Pyogenic Liver Abscess; UTI = Ulinastatin; T-α1 = Thymosin α1; HPA = Hypothalamic Pituitary Adrenal; G6PD = Glucose-6-Phosphate Dehydrogenase; SAH = Subarachnoid Hemorrhage; MV = Mechanical Ventilation; NSAIDs = Non-Steroidal Anti-Inflammatory Drugs; HLA-DR = Human Leukocyte Antigen–DR isotype; GM-CSF = Granulocyte-Macrophage Colony-Stimulating Factor; ALC = Absolute Lymphocyte Count; MAP = Mean Arterial Pressure; PVD = Perfused Vessel Density; MFI = Microvascular Flow Index; SS = Septic Shock; AIDS = Acquired Immunodeficiency Syndrome; TBSA = Total Body Surface Area; CRP = C-Reactive Protein; PCT = Procalcitonin; SOFA = Sequential Organ Failure Assessment

**Appendix 4: Characteristics of participants, treatment plans of trials and outcomes in the network meta-analysis**

| **id** | **Study,year** | **Country** | **Design** | **Cases**  **(T/C)** | **Age(year)** | **Sex(M/F)** | **Intervention(T/C)** | **Dosage** | **Duration** | **Primary outcomes** | **Secondary outcomes** |
| --- | --- | --- | --- | --- | --- | --- | --- | --- | --- | --- | --- |
| 1 | Gallagher 2001 | USA | multicenter, open-label, randomized placebo-controlled study | 27/9 | T:(57.7±16.4)  C:(67.2±18.9) | T:5/4  C:13/14 | TNF-α MAb（afelimomab）/Placebo | 0.3/1.0/3.0 mg/kg | 72h(every 8 h for a total of nine doses, each infused over 20 min) | Pharmacokinetic parameters, safety(AE, immunogenicity) | 28d mortality, serum TNF-a and IL-6 concentrations |
| 2 | Cohen 1996 | Multiple countries | international, multicenter, prospective, randomized, placebo-controlled double-blind trial | 287/133 | T:(57.0±17.2)  C:(57.0±17.3) | T:170/117  C:80/53 | TNF-α MAb（BAY×1351）/Placebo | 3/15 mg/kg | single intravenous | 28d all-cause mortality | shock reversal time, organ failure occurrence, safety (AE, immune response) |
| 3 | Panacek 2004 | North America | multicenter, randomized, double-blind, placebo-controlled trial | 1305/1329 | T:(58.8±17.0)  C:(59.9±17.0) | T:810/495  C:775/554 | TNF-α MAb（afelimomab）/Placebo | 1 mg/kg | 72h(every 8 h for a total of nine doses, each infused over 15 min) | 28d all-cause mortality | MOD, SOFA scores; serum TNF-a and IL-6 concentrations; safety (AE, secondary infections) |
| 4 | Dhainaut 1995 | France and Belgium | multicenter, open-dosage, placebo-controlled, blinded, clinical trial | 32/10 | T:(61±15)  C:(68±13) | T:25/7  C:5/5 | TNF-α MAb（CDP571）/Placebo | 0.1/0.3/1.0/  3.0 mg/kg | single intravenous | Safety,pharmaco-  -kinetics, immunogenicity, cytokine concentrations |  |
| 5 | Abraham 1998 | USA and Canada | multicenter, randomized, double-blind, placebo-controlled trial | 949/930 | T:(59.2±17.1)  C:(59.0±17.2) | T:574/375  C:563/367 | TNF-α MAb/Placebo | 7.5 mg/kg | single intravenous | 28d all-cause mortality | shock reversal, new organ failure occurrence, coagulopathy, AE |
| 6 | Clark 1998 | New Zealand | Singlecenter, randomized, double-blind, placebo-controlled trial | 28/28 | T:(56.5±15.2)  C:(51.6±14.9) | T:19/9  C:13/15 | TNF-α MAb（cA2）/Placebo | 300mg（4-5mg/kg） | single intravenous | Cytokine concentrations, physiological indicators |  |
| 7 | Abraham 1995 | USA and Canada | Randomized,prospective, multicenter,double-blind,placebo-controlled clinical trial | 645/326 | T:(59.3±17.0)  C:(60.2±17.5) | T:376/269  C:184/142 | TNF-α MAb/Placebo | 15/7.5 mg/kg | single intravenous | 28d all-cause mortality |  |
| 8 | Reinhart 2001 | Europe and Israel | Multicenter, double-blind, randomized, placebo-controlled study | 224/222 | / | T:144/80  C:134/88 | TNF-α MAb（afelimomab）/Placebo | 1 mg/kg | 72h(every 8 h for a total of nine doses, each infused over 15 min) | 28d all-cause mortality |  |
| 9 | Angus 2000 | USA | multicenter, randomized, double-blind, placebo-controlled trial | 546/544 | T:(60.9±16.4)  C:(60.5±16.7) | T:295/251  C:305/239 | Anti-Endotoxin MAb（E5）/Placebo | 2 mg/kg | twice intravenous infusion 24h apart | 14d mortality | 28d mortality,AE,  mortality of the non-shock group |
| 10 | Wortel 1992 | the Netherlands and US | prospective, randomized, double- blind, multicenter, placebo-controlled trial | 41/41 | T:(57.0±16.7)  C:(57.6±14.0) | / | Anti-Endotoxin MAb（HA-lA）/Placebo | 100mg | single intravenous | 28d all-cause mortality,serum TNF-a and IL-6 concentrations |  |
| 11 | McCloskey 1994 | USA | Large, simple, group-sequential, randomized, double-blind, multicenter, placebo-controlled trial | 328/293 | / | / | Anti-Endotoxin MAb（HA-lA）/Placebo | 100mg | single intravenous | 14d all-cause mortality,AE |  |
| 12 | Ziegler 1991 | USA,Canada and Europe | Randomized,prospective,  multicenter,double-blind,placebo-controlled clinical trial | 105/92 | T:(58.0±17.7)  C:(62.3±15.1) | T:62/43  C:53/39 | Anti-Endotoxin MAb（HA-lA）/Placebo | 100mg | single intravenous | 28d all-cause mortality |  |
| 13 | Greenberg 1992 | USA | Singlecenter, randomized, double-blind, placebo-controlled trial | 23/9 | / | T:16/7  C:4/5 | Anti-Endotoxin MAb（E5）/Placebo | 2.5/7.5 mg/kg | twice intravenous infusion 24h apart | Satety(AE,skin test,antimurine antibodies);3d,  7d,21d survival rate;clinical outcomes(BP,urine output,WBC,T) |  |
| 14 | Bigatello 1994 | USA | multicenter, randomized, double-blind, placebo-controlled trial | 30/33 | T:(56±19)  C:(54±14) | T:21/9  C:26/7 | Anti-Endotoxin MAb（HA-lA）/Placebo | 100mg | single intravenous | Respiratory(PaO2/  FiO2,Chest X-ray score,ARDS severity score),28d mortality,MV duration |  |
| 15 | Reinhart 2004 | Germany and the Netherlands | multicenter, randomized, double-blind, placebo-controlled trial | 32/8 | T:(59.4±19.5)  C:(61.0±13.9) | T:23/9  C:7/1 | CD14 MAb（IC14）/Placebo | Single Dose: 1/4 mg/kg;Mutiple Dose: 4×4/4＋2×3 mg/kg | single (1 mg/kg or 4 mg/kg) or multiple doses (4 mg/kg daily for 4 days, or 4 mg/kg on day 1 followed by 2 mg/kg daily for 3 days) | Safety(AE,SAE),  pharmacokinetics,  changes in inflammatory markers | 28d all-cause mortality,MOD |
| 16 | Hotchkiss 2019 | USA | multicenter, randomized, double-blind, placebo-controlled trial | 20/4 | T:(58.4±13.9)  C:(49.2±11.9) | T:9/11  C:3/1 | anti-PD-L1（BMS-936559）/Placebo | 10/30/100/300/  900mg | single intravenous | 90d mortality,AE | Pharmacokinetics  (PK);mHLA-DR expression;IL-6,  IL-8,IL-10 |
| 17 | Albertson 2003 | USA | multicenter, randomized, double-blind, placebo-controlled trial | 411/415 | T:(57.9±17.2)  C:(57.2±17.5) | T:245/166  C:253/162 | MAB-T88/Placebo | 300mg | single intravenous | 28d all-cause mortality | AE |
| 18 | Laterre 2021 | Belgium, France, Germany and the Netherlands | multicenter, randomized, double-blind, placebo-controlled trial | 149/152 | T:(69.6±10.5)  C:(69.6±13.5) | T:98/51  C:86/66 | adrecizumab（HAM8101）/Placebo | 2/4 mg/kg | single intravenous infusion（duration: approximately 1h） | 90d all-cause mortality,TEAEs | Sepsis Support Index (SSI),SOFA scores,28d mortality,ICU-  LOS |
| 19 | Tugrul 2002 | Turkey | Singlecenter, prospective, randomized controlled study | 21/21 | T:(42.0±18)  C:(49.3±20.6) | T:12/9  C:10/11 | Plg(Pentaglobin®)/No intervention | 5 ml/kg/d | 3d（infused intravenously over 6h） | SOFA,APACHEⅡ,PCT levels,septic shock incidence,28d mortality,MV duration and ICU-LOS |  |
| 20 | Toth 2013 | Hungary,UK | Singlecenter, prospective, randomized controlled study | 16/17 | T:(57.1±12.2)  C:(57.5±10.5) | T:8/8  C:4/13 | IgM-enriched IVIG（Pentaglobin®）/Placebo | 5 ml/kg/d | 3d | MODS scores,28d mortality,CRP,PCT levels |  |
| 21 | Rodríguez 2005 | Spain and Argentina | Randomized,prospective,  multicenter,double-blind,placebo-controlled clinical trial | 29/27 | T:(61.3±19.9)  C:(65.9±18.2) | T:15/14  C:12/15 | IgM-enriched IVIG（Pentaglobin®）/Placebo | 7 ml/kg/d | 5d | mortality,MODS scores,reoperation rate,ICU-LOS |  |
| 22 | Hentrich 2006 | Germany | multicenter, open-label, randomized controlled study | 103/103 | T:(48.7±11.6)  C:(51.2±13.8) | T:63/39  C:58/45 | ivIGMA（Pentaglobin）/Placebo | 1300ml | infused intravenously within 72h | 28d all-cause mortality,28d sepsis-related mortality | 60d mortality,shock mortality,Gram-  negative bacterial infection mortality |
| 23 | Domizi 2019 | Italy | Singlecenter, randomized, double-blind, placebo-controlled trial | 10/9 | T:(62±20)  C:(67±16) | T:7/3  C:8/1 | IgM-enriched IVIG（Pentaglobin）/Placebo | 250 mg/kg/d(5 ml/kg/d) | continuous intravenous infusion for 72h | Perfused Vessel Density(PVD),  Microvascular Flow Index(MFI) | cytokine levels,SOFA scores |
| 24 | De Simone 1988 | Italy | Singlecenter, open-label, randomized controlled study | 12/12 | T:(45±4)  C:(45±5) | T:5/7  C:7/5 | IVIG(Sandoglobulin)+antibiotic/antibiotic | Initial dose of 0.4 g/kg, followed by 0.2 g/kg after 48 hours, and an additional 0.4 g/kg after 5 days if clinically necessary | mean 14d | Survival,Deferves--cence time,  Negativization of cultures,  Percentage of days on antibiotic treatment |  |
| 25 | Brunner 2013 | Austria | Singlecenter, prospective, randomized, double-blinded and placebo-controlled trial | 19/19 | T:(61±11)  C:(66±12) | T:12/7  C:9/10 | IgM-enriched IVIG（Pentaglobin）/Placebo | 0.25 g/kg/d | 3d | CIPNM severity  sum score | 28d all-cause mortality,ICU-  LOS |
| 26 | Hall 2016 | UK | Singlecenter, randomized controlled study | 30/30 | 64.1±12.2 | 33/27 | PUFA（Omegaven™）/No intervention | 0.092 g EPA+DHA/kg/d | Intravenous infusion, once daily for 14 days or until discharge/death | Fatty acid profiles in plasma PC, NEFAs, and PBMCs | 28d mortality |
| 27 | Galbán 2000 | Spain | multicenter, open-label, prospective,randomized controlled study | 89/87 | T:(52.9±15.5)  C:(57.7±16.9) | T:64/25  C:65/22 | Impact®(enriched with arginine, mRNA, and ω-3 fatty acids from fish oil)/high protein control feed | Caloric needs based on the Harris- Benedict formula | Continued until ICU discharge | Mortality,Episodes of bacteremia,  Nosocomial infections and ICU-LOS |  |
| 28 | Bower 1995 | USA | prospective, randomized, double-blind, multicenter clinical study | 147/132 | T:(39±18.2)  C:(39.9±18.2) | / | Impact(supplemented with arginine, dietary nucleotides, and fish oil)/Osmolite HN®(common use enteral formula) | 821ml/d | at least 7d | Hospital-LOS,  infectious complications,  mortality |  |
| 29 | Burkhart 2014 | Switzerland | Singlecenter, double-blind, randomized controlled study | 25/25 | T:(72±9)  C:(66±13) | T:13/12  C:14/11 | PUFA（Omegaven™）/No intervention | 2ml/kg/d  (equivalent to 0.12 mg/kg/day of n-3 fatty acids) | 7d | Changes in S-100β level | NSE,IL-6,IL-8,IL-10,CRP levels;  Incidence of sepsis-associated delirium;Survival rate |
| 30 | CHEN 2017-01 | China | Singlecenter, single-blind,randomized controlled study | 24/24 | T:(61.6±16.2)  C:(65.6±16.7) | T:18/6  C:16/8 | Omega-3/No intervention | 10g/d | 7d | 28d mortality;  APACHE II,Marshall scores;CD4/CD8 |  |
| 31 | Hosny 2013 | Egypt | Singlecenter,open-label,  randomized controlled study | 50/25 | T(high dose):  (52.8±18.87)  C:(50.5±14.77) | T(high dose):  10/15,  C:13/12 | Omega-3/No intervention | High dose: 9 g in 3 divided doses Low dose: 3 g in 3 divided doses | 7d | CRP,IL-6,PCT levels;SOFA scores;MV duration;28d mortality |  |
| 32 | Ibrahim 2018 | Egypt | Singlecenter, randomized, double-blind, placebo-controlled trial | 55/55 | T:(61.2±7.63)  C:(62.35±8.15) | T:29/26  C:28/27 | Omega-3(DHA＋EPA)/Placebo | 3000mg in 3 divided doses | Continued until ICU discharge | SOFA score,Organ failure-free days,Organ failure-  dysfunction days,Hemodynamic failure-free days,WBC,CRP levels,ICU-LOS,ICU mortality and in-hospital mortality |  |
| 33 | Pontes-Arruda 2006 | Brazil | multicenter, prospective, randomized, double-blind, placebo-controlled trial | 55/48 | T:(64.3±18.7)  C:(66±20) | T:35/20  C:26/22 | PUFA(enriched with EPA, GLA, and elevated antioxidants)/Placebo | achieve a minimum of 50% basal energy expenditure (BEE;determined using the Harris-Benedict equation) × 1.3 within the first 24 hrs, if well tolerated, advanced to achieve a minimum of 75% of BEE ×1.3 within 72 hrs | at least 4d,until patients were extubated or until interrupted at physician's discretion or due to the development of any adverse event that could be related to the enteral feeding | 28d all-cause mortality,PaO2/  FiO2,ICU-free days and ventilator free days |  |
| 34 | Barbosa 2010 | Portugal | Singlecenter, single-blind,randomized controlled study | 13/10 | T:(70±2)  C:(57±5) | T:5/8  C:4/6 | 50:40:10 mixture of medium-chain fatty acids, soybean oil and fish oil/50:50 mixture of medium-chain fatty acids and soybean oil | 6.4 g/d fish oil(equivalent to 2.3 g EPA+DHA/d) | 5d | Plasma phospha-  -tidylcholine fatty acid composition, Plasma cytokine and eicosanoid concentrations, PO2/FiO2, MV duration and ICU-LOS |  |
| 35 | CHEN 2017-02 | China | Singlecenter, double-blind, randomized controlled study | 41/37 | T:(68.0±13.0)  C:(69.1±15.5) | T:22/19  C:21/16 | long chain fatty acid soybean oil and FO/long chain fatty acid soybean oil | 50 g of long chain fatty acid soybean oil and 10 g of FO | 7d | 60d mortality, Marshall scores,T lymphocyte subsets |  |
| 36 | Bone 1987 | USA | multicenter, randomized, double-blind, placebo-controlled trial | 191/191 | T:(53.0±16)  C:(53.7±16) | 235/147 | methylprednisolone sodium succinate/placebo | 30mg/kg | 24h(every 6 hours,for a total of 4 doses) | Shock prevention and reversal,14d mortality |  |
| 37 | Briegel 2022 | Germany | multicenter, randomized, double-blind, placebo-controlled trial | 90/90 | T:(64.3±14.7)  C:(62.9±15.7) | T:60/30  C:53/37 | Hydrocortisone/Placebo | 200mg/d | 11d(for 5 days and a gradual dose reduction for another 6 days) | Septic shock incidence, in-hospital mortality, 28d mortality and 90d mortality |  |
| 38 | Agarwal 2021 | Indian | Singlecenter, single-blind,randomized controlled trial | 61/59 | T:(68.3±6.3)  C:(66.7±4.6) | T:40/21  C:33/26 | Hydrocortisone/No intervention | 200mg/d | intravenously in four divided doses, then tapered (100mg ×3 days → 50mg ×3 days → stop) after vasopressor withdrawal | 28d mortality |  |
| 39 | Lv 2017 | China | Singlecenter, randomized, double-blind, placebo-controlled trial | 58/60 | T:(68.8±12.6)  C:(64.8±16.7) | T:33/25  C:37/23 | Hydrocortisone/Placebo | 200mg/d | Continuous infusion for 6 days, then tapered (half dose ×3 days → quarter dose ×3 days) after vasopressor withdrawal | 28d all-cause mortality |  |
| 40 | Annane 2006 | France | multicenter, randomized, double-blind, placebo-controlled trial | 85/92 | T:(61±16)  C:(59±18) | T:56/29  C:65/27 | Steroids/Placebo | 50 mg of hydro-  -cortisone every 6 hrs  and 50 μg of 9--fludrocorti--sone once a day | 7d | 28d mortality,ICU mortality and Hospital mortality |  |
| 41 | Sevransky 2021 | USA | multicenter, randomized, double-blind, placebo-controlled trial | 252/249 | T:(60.6±13.4)  C:(61±16.4) | T:139/113  C:134/115 | Vitamin C,Thiamine and Steroids/Placebo | VitaminC(1.5g),Thiamine(100mg) and Steroids(50mg) every 6 hrs | Continuous infusion for 96h or ICU discharge | the number of consecutive ventilator- and vasopressor-free days in the first 30 days |  |
| 42 | Moskowitz 2020 | USA | multicenter, randomized, double-blind, placebo-controlled trial | 101/99 | T:(68.9±15.0)  C:(67.7±13.9) | T:57/44  C:54/45 | Vitamin C,Thiamine and Steroids/Placebo | VitaminC(1.5g),Thiamine(100mg) and Steroids(50mg) every 6 hrs | 4d | 72h △SOFA score |  |
| 43 | Arabi 2010 | Saudi Arabia | Singlecenter, randomized, double-blind, placebo-controlled trial | 39/36 | T:(60.6±12.6)  C:(59.3±12.2) | T:22/17  C:20/16 | Hydrocortisone/Placebo | 50mg every 6 hrs | until hemodynamic stability, then tapered over 8d | 28d all-cause mortality |  |
| 44 | Bollaert 1998 | France | multicenter, randomized, double-blind, placebo-controlled trial | 22/19 | T:(66±21)  C:(56±34) | T:15/7  C:12/7 | Hydrocortisone/Placebo | 100mg | iv,tid,5d | Shock reversal within 7d and 15d,28d all-cause mortality |  |
| 45 | Birudaraju 2022 | USA | Singlecenter,prospective, randomized, double-blind, placebo-controlled clinical trial | 21/22 | 51±2 | T:13/8  C:14/8 | Solumedrol / Placebo | 20 mg | every 8 h for 7 days | 28d mortality | 14d mortality,MV duration,vasopre--ssor support time,ICU-LOS,  APACHE II scores |
| 46 | Annane 2002 | France | Multicenter,placebo-  controlled, randomized, double-blind, parallel-group trial | 150/149 | T:(62±15)  C:(60±17) | T：96/54  C:104/45 | Hydrocortisone and fludrocortisone/Placebo | hydrocortisone 50mg,fludrocor--tisone 50 µg | hydrocortisone intravenous bolus every 6 hours and fludrocortisone tablet once daily for 7 days | 28d survival distribution | 28d survival rate,ICU mortality,  in-hospital mortality,time to vasopressor therapy withdrawal,AE |
| 47 | Peduzzi 1991 | USA | Multicenter randomized, double-masked trial | 112/111 | / | / | Steroid/Placebo | 30 mg/kg or 5mg/kg/hr | 30mg/kg infusion pump for 15 minutes, followed by a constant infusion of 5mg/kg/hr for 9 hours | 14d all-cause mortality | Incidence of adverse drug reactions and complications |
| 48 | Russell 2009 | Canada, Australia, and the United States | multicenter randomized blinded controlled trial | 589/190 | T:(60.2±16.0)  C:(61.7±17.0) | T：359/230  C:116/74 | Steroids/No Steroids | Corticosteroid administration based on the clinician's discretion (non-protocolized) | at least 1d | 28d mortality |  |
| 49 | Oppert 2005 | Germany | Prospective, randomized, double-blind, singlecenter study | 18/23 | / | T：13/5  C：19/4 | Hydrocortisone/Placebo | 50mg | 50-mg bolus followed by a continuous infusion of 0.18 mg/kg/hr, reduced to 0.06 mg/kg/hr after shock reversal and gradually tapered | Time to cessation of vasopressor support |  |
| 50 | Thompson 2020 | Australia | Multicenter,international investigator-initiated, blind, randomised controlled trial | 754/759 | T:（62.4±14.8）C:(62.8±14.8) | T：472/282  C：458/301 | Hydrocortisone /Placebo | 200 mg | 7d | mortality, health-related quality of life, and quality-adjusted life-years gained |  |
| 51 | Moreno 2011 | Europe | Multicenter, randomized, double-blind, placebo-controlled study | 251/248 | T:(62.7±15.6)  C:(63.5±15.8) | T:166/85  C:163/85 | Hydrocortisone / Placebo | 50mg | 11d | 28d mortality |  |
| 52 | Antcliffe 2018 | UK | factorial (2x2), multicenter, double-blind, randomized clinical trial | 58/59 | / | T;35/23  C:36/23 | Hydrocortisone / Placebo | 50mg | Intravenous injection every 6 hours, for a total of 5 days or until shock is relieved | 28d survival rate |  |
| 53 | Mohanty 2024 | Indian | Singlecenter, randomized, placebo-controlled trial | 23/23 | / | 25/21 | Hydrocortisone / Placebo | 50mg | every 6 hrs for 7 days | 28d mortality |  |
| 54 | Wunderink 2001 | USA | Multicenter, double-blind, randomized, placebo-controlled study | 12/6 | / | T:8/4  C:4/2 | Filgrastim/Placebo | 300μg/d | 5d | AE |  |
| 55 | root 2003 | USA, Canada, Australia, and Europe | Multicenter, double-blind, placebo-controlled study | 348/353 | T:(58.9±17.1)  C:(60.0±16.4) | T:240/108  C:247/106 | Filgrastim/Placebo | 300μg/d | 5d | 29d mortality |  |
| 56 | stephens 2008 | Australia | Singlecenter,randomized, placebo-controlled, double-blinded clinical trial | 81/83 | T:(51.0±15.1)  C:(48.9±16.1) | T:44/37  C:45/38 | G-CSF/placebo | 263μg | intravenously daily for 10 days | hospital mortality |  |
| 57 | Tanaka 2001 | Japan | Singlecenter,prospective randomized study | 12/13 | T:(49.8±6.4)  C:(54.8±5.7) | T:11/1  C:9/4 | G-CSF/placebo | 2μg/kg | once a day for 5 days |  |  |
| 58 | Meisel 2009 | Germany | prospective, randomized, double-blind, placebo-controlled, multicenter trial | 19/19 | T:(64.0±13.6)  C:(63.3±14.2) | T:16/3  C:15/4 | GM-CSF/placebo | 4μg/kg/d | 8d | mHLA-DR expression |  |
| 59 | Francois 2018 | USA and France | Multicenter,prospective, randomized, double-blind, placebo-controlled trial | 17/10 | / | T:13/4  C:8/2 | IL-7(CYT107) / Placebo | 10μg/kg | 4 weeks(twice a week for the first week ,low frequency group once a week,the high frequency group twice a week) | absolute lymphocyte count |  |
| 60 | Daix 2023 | USA and France | prospective, multicenter, randomized, double blind, placebo-controlled phase IIb trial | 15/6 | / | T:13/2  C;4/2 | IL-7(CYT107) / Placebo | 10μg/kg | 90d | change in ALC at day 29 |  |
| 61 | Zhou 2020 | China | multicenter,randomized controlled trial | 13/16 | T:(68.85±11.62)  C:(64.94±10.47) | / | UTI/No intervention | 100,000U | intravenous infusion during a period of 8 h | 28d mortality |  |
| 62 | Tiejun 2013 | China | singlecenter,randomized controlled trial | 30/30 | 54.3±16.2 | / | UTI/No intervention | 300kU | intravenous infusion, three times per day, consisted of 5 days | 28d mortality |  |
| 63 | He 2022 | China | singlecenter，randomized controlled trial | 50/50 | T:(41.36±5.24)  C(42.03±4.86) | T:36/14  C:34/16 | UTI/Placebo | 500,000 IU | intravenous pumping,for 7 consecutive days | ICU-LOS,hospital-  LOS, in-hospital mortality |  |
| 64 | Karnad 2014 | Indian | multicenter, randomized, double-blind, placebo-controlled trial | 55/59 | T:(37.5±12.9)  C:(36.7±12.5) | T:38/17  C:50/9 | UTI/Placebo | 200,000 IU | intravenously over 1 h every 12 h for 5 days | 28d all-cause mortality |  |
| 65 | Guo 2023 | China | singlecenter,randomized,  controlled trial | 48/51 | T:(61.06±8.20)  C:(59.35±10.78) | T:31/17  C:27/24 | UTI/No intervention | 200,000 IU | q8h for ＞3 days | indicators related to infection,  recovery of liver function indices |  |
| 66 | Wu 2013 | China | prospective, controlled, single-blinded, multicenter randomized clinical trial | 181/180 | T:(64.7±14.5)  C:(66.4±12.6) | T:141/40  C:131/49 | Tα1/Placebo | 1.6 mg | subcutaneous injection twice per day for five consecutive days, then once per day for two consecutive days | 28d all-cause mortality |  |
| 67 | Zhou 2009 | China | singlecenter prospective, randomized controlled trial | 45/46 | T:(58.95±19.87)  C:(61.91±19.41) | T:29/16  C:31/15 | Tα1/No intervention | 1.6 mg | subcutaneous injection once a day for 7 days | ICU-LOS, 28d MV duration, 28d mortality, and 90d mortality |  |
| 68 | Bai 2022 | China | singlecenter randomized controlled study | 43/43 | T:(65.63±7.55)  C:(66.77±6.35) | T:20/23  C:24/19 | Tα1/No intervention | 1.6 mg | subcutaneous injection,twice a week, with an injection interval of three days,continuously for 10 days | duration of shock, ICU-LOS, and incidence of adverse reactions |  |
| 69 | Chen 2007 | China | singlecenter,randomized,  controlled trial | 21/21 | / | T:13/8  C:14/7 | Tα1/No intervention | 1.6 mg | subcutaneous injection twice daily for 1 week | ICU-LOS, MV duration, total hospitalization cost and 28d mortality |  |
| 70 | Wu 2025 | China | Multicentre, double blinded, placebo controlled phase 3 trial | 542/547 | T:(63.6±16.35)  C:(62.55±15.61) | T:360/182  C:390/157 | Tα1/Placebo | 1.6 mg | subcutaneous injection every 12h for 7d | 28d all-cause mortality |  |
| 71 | Huang 2009 | China | singlecenter,prospective, randomized and placebo controlled trial | 36/34 | T:(55±18)  C:(53±13) | T:30/6  C:28/6 | UTI plus Tα1/Placebo | UTI：200 000 U/100 000 U  Tα1: 1.6mg | 200,000U UTI tid＋1.6mg Tα1 bid（the first 3 days）→ 100,000U UTI tid＋1.6mg Tα1 qd（the last 4 days） | cumulative survival rates |  |
| 72 | Su 2009 | China | singlecenter,randomized,controlled trial | 128/114 | T:(56.9±17.2）  C:(54.7±16.3） | T:85/43  C:89/25 | UTI plus Tα1/No intervention | UTI：200 000 U/100 000 U  Tα1: 1.6mg | 200,000U UTI bid＋1.6mg Tα1 bid（the first 4 days）→ 100,000U UTI bid＋1.6mg Tα1 qd（the last 4 days） | Duration of infection and MV，length of ICU stay，rate of development of MODS and 28d mortality |  |
| 73 | Lin 2007 | China | multicenter,prospective randomized controlled trial | 175/167 | T:(55.37±19.41)  C:(56.59±16.63) | T:131/44  C:128/39 | UTI plus Tα1/No intervention | UTI：200 000 U/100 000 U  Tα1: 1.6mg | 100,000U UTI tid＋1.6mg Tα1 qd（the first 7 days）→ 200,000U UTI tid＋1.6mg Tα1 bid（the last 7 days） | 28d mortality，90d mortality |  |
| 74 | Chen 2009 | China | singlecenter,prospective, randomized, controlled pilot study | 59/55 | T:(50±7.1)  C:(53±7.9) | T;36/23  C:33/32 | UTI plus Tα1/Placebo | UTI：200 000 U/100 000 U  Tα1: 1.6mg | 200,000U UTI tid＋1.6mg Tα1 bid（the first 3 days）→ 100,000U UTI tid＋1.6mg Tα1 qd（the last 4 days） | APACHE II, MOF and GCS，resolution of pre-existing organ dysfunction,the survival rate at 28, 60 and 90 days |  |
| 75 | Li 2009 | China | singlecenter,prospective, double-blinded clinical trial | 23/33 | T:(50±7.1)  C:(53±7.9) | T:14/9  C:19/14 | UTI plus Tα1/Placebo | UTI：200 000 U/100 000 U  Tα1: 1.6mg | 200,000U UTI tid＋1.6mg Tα1 bid（the first 3 days）→ 100,000U UTI tid＋1.6mg Tα1 bid（the last 4 days） | 28d cumulative survival rate |  |
| 76 | Zhang 2008 | China | Singlecenter, randomized, double-blind, placebo-controlled trial | 59/55 | T:(52±6.3)  C:(53±7.1) | T:34/25  C:31/24 | UTI plus Tα1/Placebo | UTI：200 000 U/100 000 U  Tα1 1.6mg | 200,000U UTI tid＋1.6mg Tα1 tid → 100,000U UTI tid＋1.6mg Tα1 qd(3d after) | 28d,60d,90d cumulative survival rate;APACHE II,MOF,GCS scores;Lymphocyte subsets and cytokine levels |  |

**Appendix 5: Potential risk of bias of each included randomized controlled trials.**

| **id** | **Author,year** | **Random sequence generation** | **Allocation concealment** | **Blinding of participants and personnel** | **Blinding of outcome assessment** | **Incomplete outcome data** | **Selective reporting** | **Other bias** | **Overall bias** |
| --- | --- | --- | --- | --- | --- | --- | --- | --- | --- |
| 1 | Gallagher 2001 | low | unclear | high | unclear | low | low | low | high |
| 2 | Cohen 1996 | low | low | low | unclear | low | low | low | moderate |
| 3 | Panacek 2004 | low | low | low | low | low | low | low | low |
| 4 | Dhainaut 1995 | unclear | unclear | low | low | low | low | unclear | moderate |
| 5 | Abraham 1998 | low | low | low | low | high | low | low | high |
| 6 | Clark 1998 | low | low | low | low | low | low | low | low |
| 7 | Abraham 1995 | unclear | unclear | low | low | high | low | low | high |
| 8 | Reinhart 2001 | low | low | low | low | low | low | low | low |
| 9 | Angus 2000 | unclear | unclear | low | low | high | low | unclear | high |
| 10 | Wortel 1992 | low | unclear | low | unclear | low | low | unclear | moderate |
| 11 | McCloskey 1994 | low | low | low | low | high | low | unclear | high |
| 12 | ZIEGLER 1991 | low | low | low | low | high | low | unclear | high |
| 13 | GREENBERG 1992 | low | unclear | low | unclear | high | low | low | high |
| 14 | Bigatello 1994 | low | unclear | low | unclear | low | low | low | moderate |
| 15 | Reinhart 2004 | low | low | low | low | low | low | low | low |
| 16 | Hotchkiss 2019 | low | low | low | low | low | low | low | low |
| 17 | Albertson 2003 | unclear | unclear | low | unclear | low | low | unclear | moderate |
| 18 | Laterre 2021 | low | low | low | low | low | low | low | low |
| 19 | Tugrul 2002 | low | high | high | unclear | low | unclear | low | high |
| 20 | Toth 2013 | low | low | high | low | low | low | low | high |
| 21 | Rodríguez 2005 | low | low | low | low | low | unclear | unclear | moderate |
| 22 | Hentrich 2006 | low | low | low | low | low | low | low | low |
| 23 | Domizi 2019 | low | low | low | low | low | low | low | low |
| 24 | De Simone 1988 | unclear | unclear | high | high | low | unclear | low | high |
| 25 | Brunner 2013 | low | low | low | low | low | low | low | low |
| 26 | Hall 2016 | low | low | high | unclear | low | low | low | high |
| 27 | Galbán 2000 | low | unclear | high | low | low | low | unclear | high |
| 28 | Bower 1995 | low | low | low | low | low | low | unclear | low |
| 29 | Burkhart 2014 | low | low | low | low | low | unclear | low | moderate |
| 30 | CHEN 2017-01 | low | unclear | high | low | low | low | low | high |
| 31 | Hosny 2013 | unclear | unclear | low | unclear | low | low | unclear | moderate |
| 32 | Ibrahim 2018 | unclear | unclear | low | unclear | low | low | low | moderate |
| 33 | Pontes-Arruda 2006 | unclear | unclear | low | low | low | low | low | moderate |
| 34 | Barbosa 2010 | unclear | unclear | high | high | high | low | unclear | high |
| 35 | CHEN 2017-02 | low | unclear | low | low | low | low | unclear | moderate |
| 36 | Bone 1987 | low | low | low | low | low | unclear | low | low |
| 37 | Briegel 2022 | low | low | low | low | low | low | low | moderate |
| 38 | Agarwal 2021 | low | low | low | unclear | low | low | low | moderate |
| 39 | Lv 2017 | low | low | low | low | low | low | low | low |
| 40 | Annane 2006 | low | unclear | low | unclear | low | low | low | moderate |
| 41 | Sevransky 2021 | low | low | low | low | low | low | low | low |
| 42 | Moskowitz 2020 | low | low | low | low | low | low | low | low |
| 43 | Arabi 2010 | low | low | low | unclear | unclear | low | high | high |
| 44 | Bollaert 1998 | low | low | low | unclear | low | low | low | moderate |
| 45 | Birudaraju 2022 | low | low | low | unclear | low | low | unclear | moderate |
| 46 | Annane 2002 | low | low | low | low | low | low | low | moderate |
| 47 | Peduzzi 1991 | low | low | low | unclear | low | low | unclear | low |
| 48 | Russell 2009 | low | low | low | low | low | low | low | low |
| 49 | Oppert 2005 | low | low | low | unclear | high | low | low | high |
| 50 | Thompson 2020 | low | low | low | low | low | low | low | low |
| 51 | Moreno 2011 | low | low | low | low | low | low | low | low |
| 52 | Antcliffe 2018 | low | low | low | low | low | low | low | low |
| 53 | Mohanty 2024 | unclear | unclear | low | unclear | low | low | unclear | moderate |
| 54 | Wunderink 2001 | unclear | unclear | low | low | low | low | low | moderate |
| 55 | root 2003 | low | unclear | low | low | unclear | low | low | moderate |
| 56 | stephens 2008 | low | low | low | low | low | low | low | low |
| 57 | Tanaka 2001 | unclear | unclear | unclear | unclear | low | low | low | moderate |
| 58 | Meisel 2009 | low | low | low | low | low | low | low | low |
| 59 | Francois 2018 | low | low | low | low | low | low | low | low |
| 60 | Daix 2023 | low | low | low | low | low | low | low | low |
| 61 | Zhou 2020 | unclear | unclear | high | high | low | low | low | high |
| 62 | Tiejun 2013 | low | unclear | high | high | low | low | low | high |
| 63 | He 2022 | low | unclear | high | high | low | low | low | high |
| 64 | Karnad 2014 | low | low | low | low | low | low | low | low |
| 65 | Guo 2023 | low | unclear | high | high | low | low | low | high |
| 66 | Jianfeng 2013 | low | low | high | high | low | low | low | high |
| 67 | Zhou 2009 | low | unclear | high | high | low | low | low | high |
| 68 | Bai 2022 | low | low | high | unclear | low | low | low | high |
| 69 | Chen 2007 | low | unclear | high | high | low | low | low | high |
| 70 | Wu 2025 | low | low | low | low | low | low | low | low |
| 71 | Huang 2009 | unclear | unclear | high | unclear | low | low | unclear | high |
| 72 | Su 2009 | unclear | unclear | high | unclear | low | low | unclear | high |
| 73 | Lin 2007 | low | low | high | unclear | low | low | unclear | high |
| 74 | Chen 2009 | low | unclear | low | unclear | low | low | low | moderate |
| 75 | Li 2009 | low | unclear | low | unclear | low | low | low | moderate |
| 76 | Zhang 2008 | low | unclear | low | unclear | low | low | low | moderate |

**Appendix 6: Pairwise Comparisons for Immunotherapy Interventions**

**1A: All-cause Mortality**

**
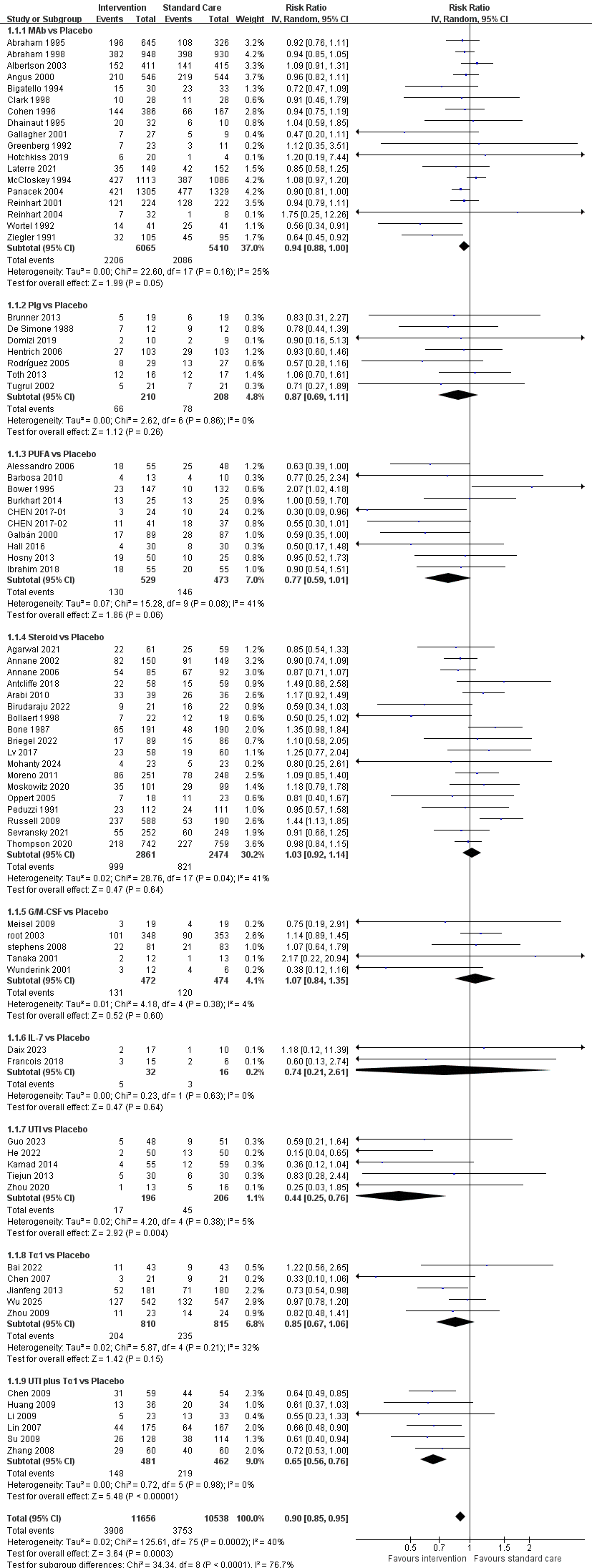
**

**
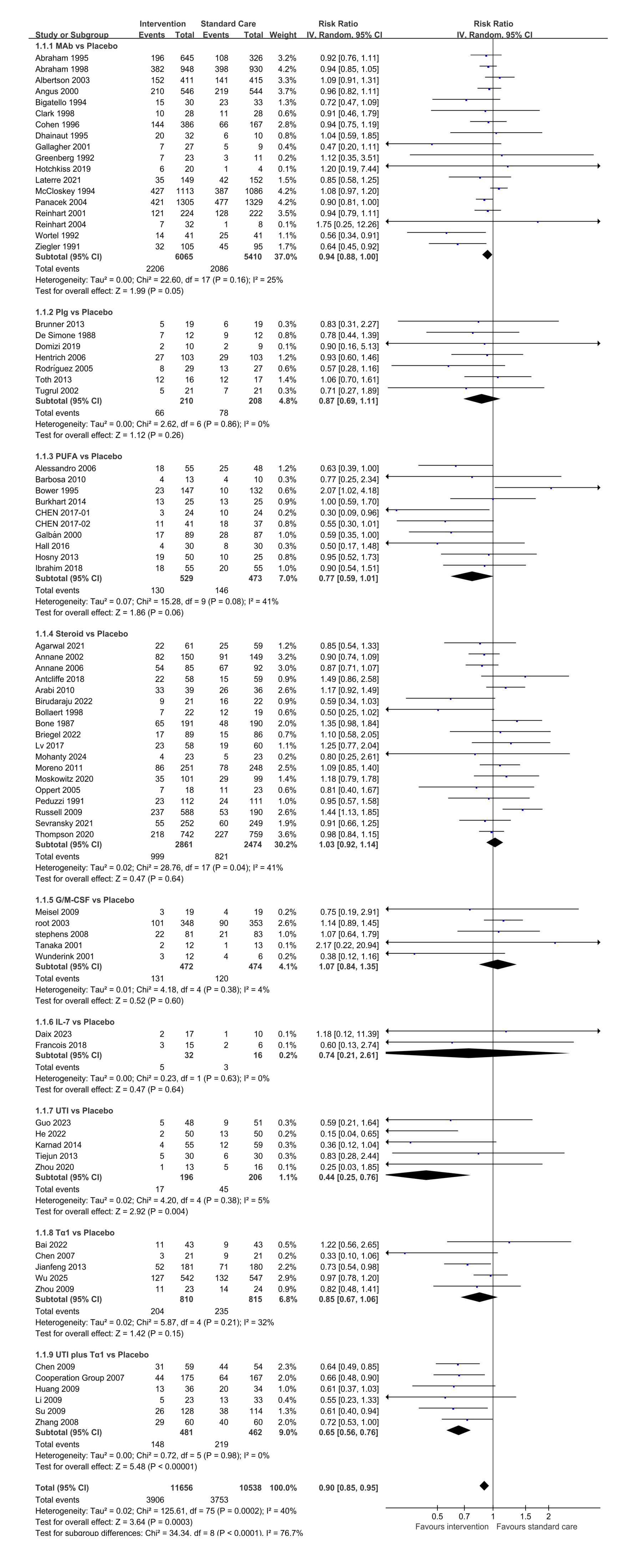
**

**1B: Length of ICU Stay**

**
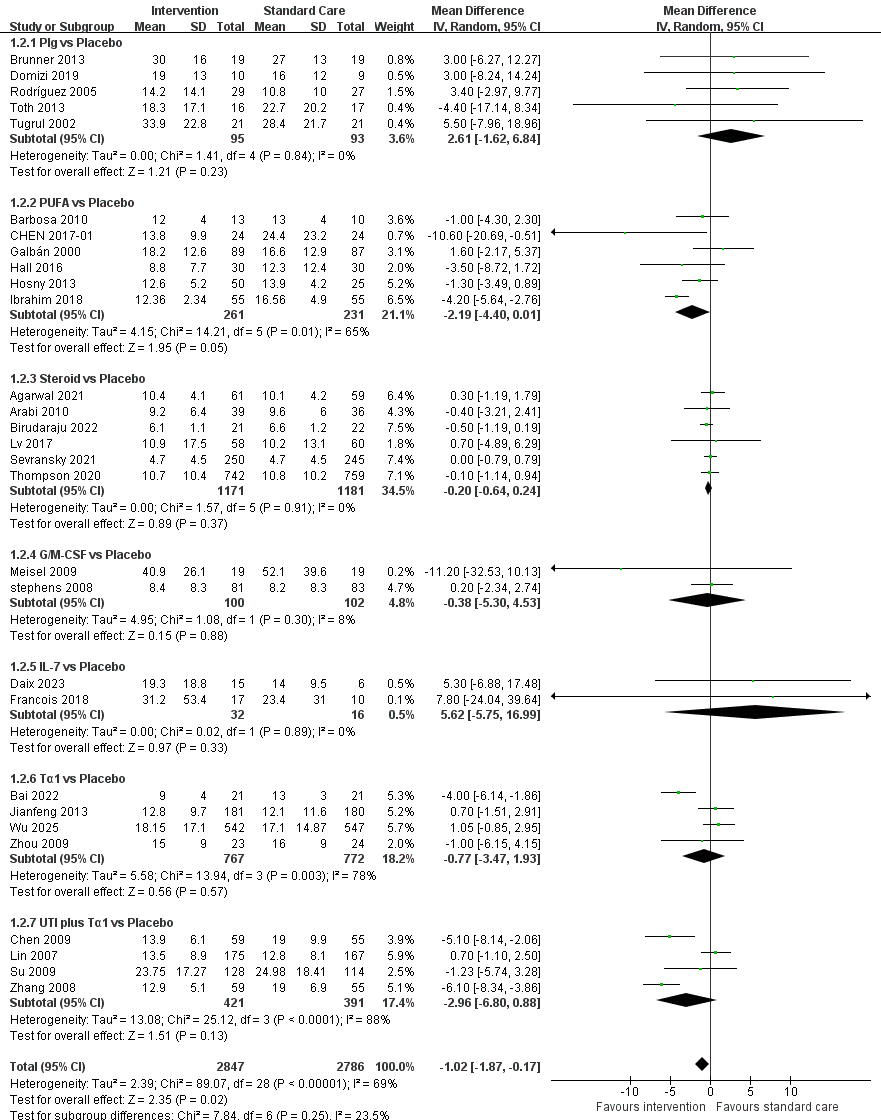
**

**1C: Length of Hospital Stay**

**
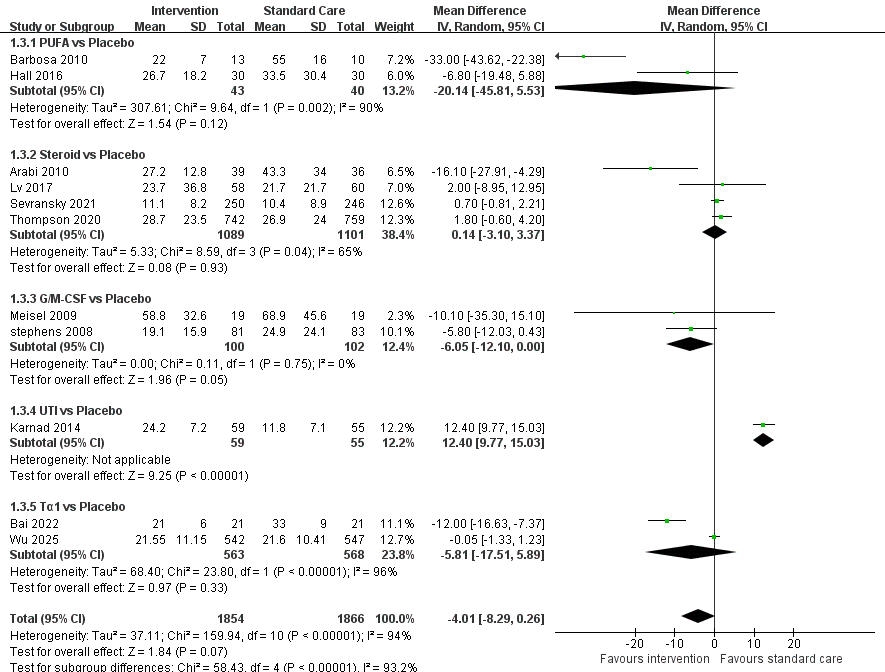
**

**1D: Duration of Mechanical Ventilation**

**
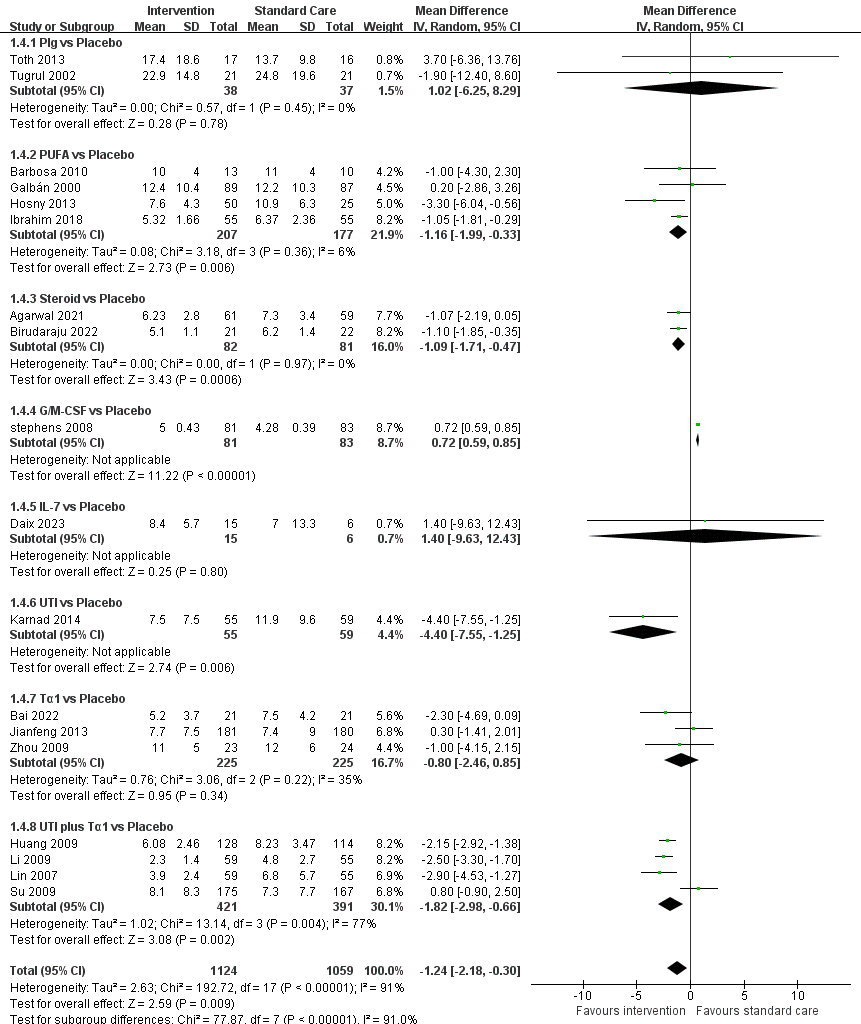
**

**1E: Adverse Event**

**
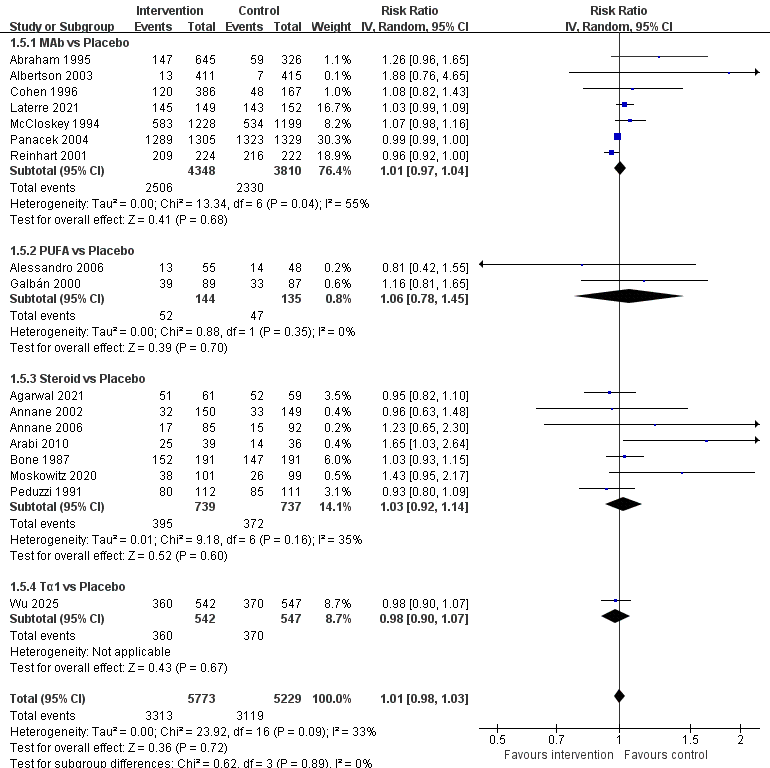
**

**1F: Serious Adverse Event**

**
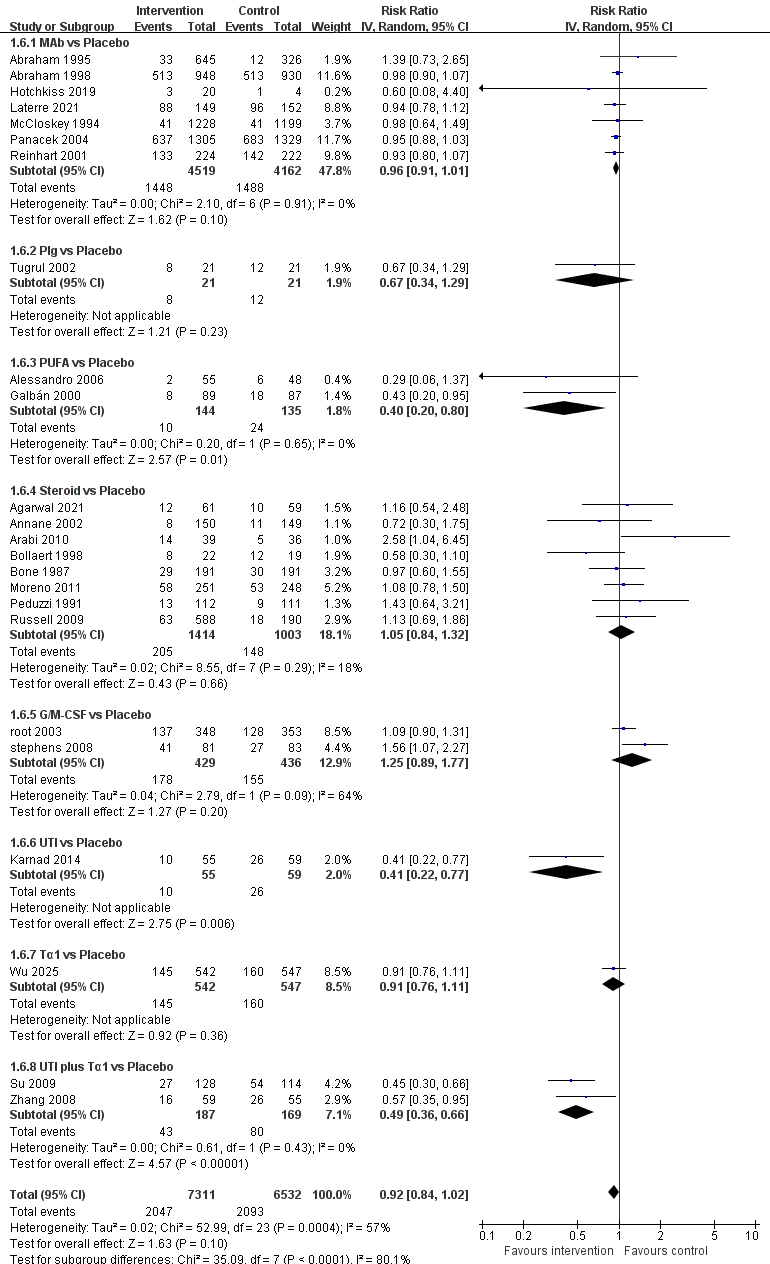
**

**
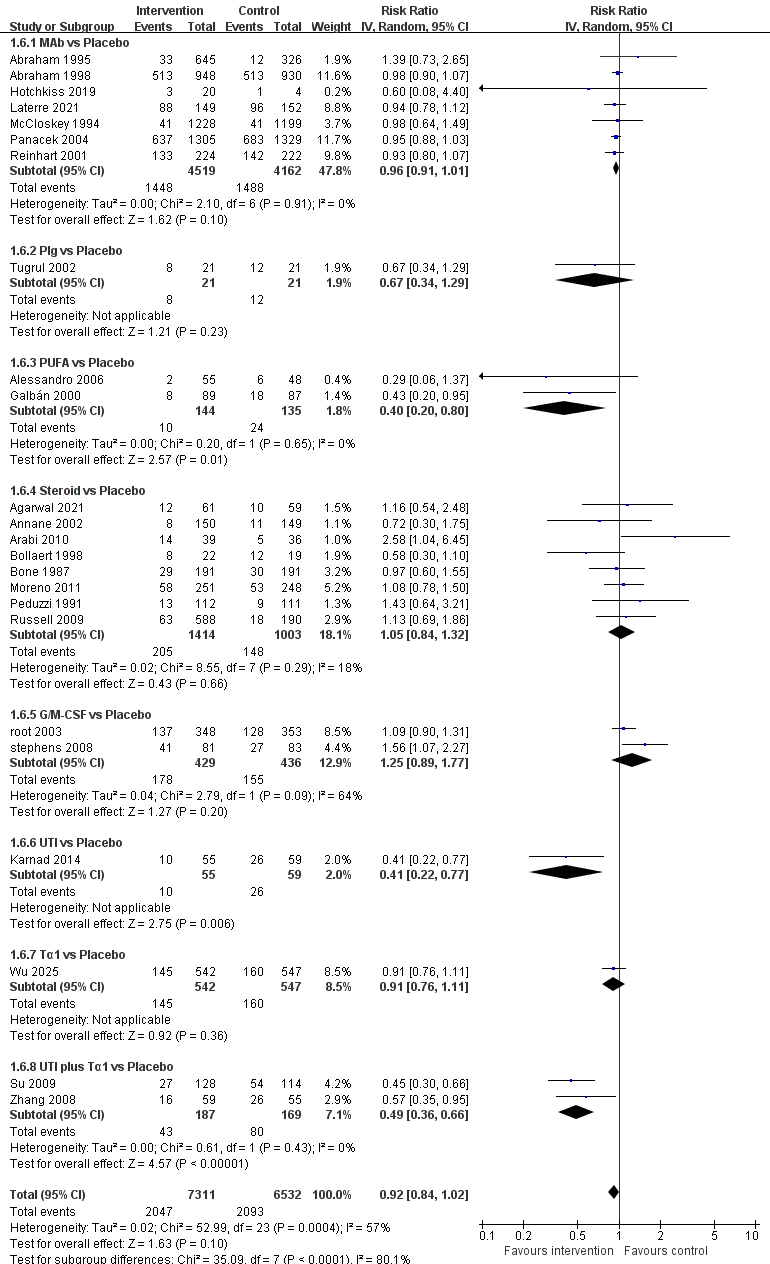
**

**Appendix 7: Model fit plots and consistency hypothesis test**

1. **All-cause Mortality**


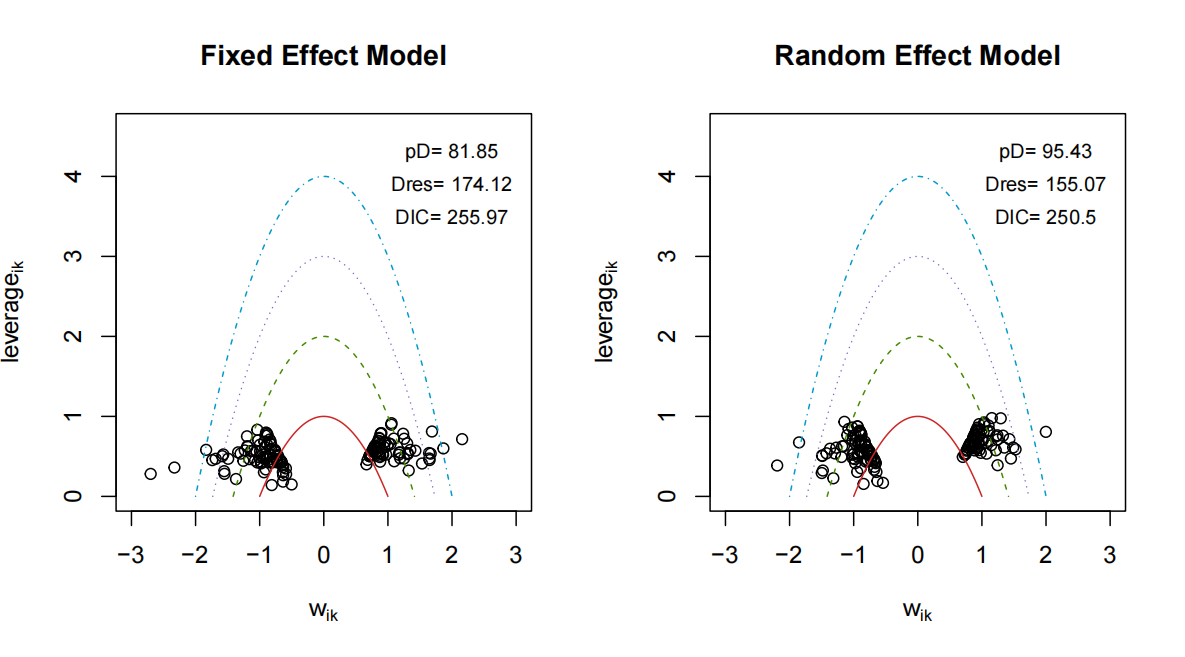


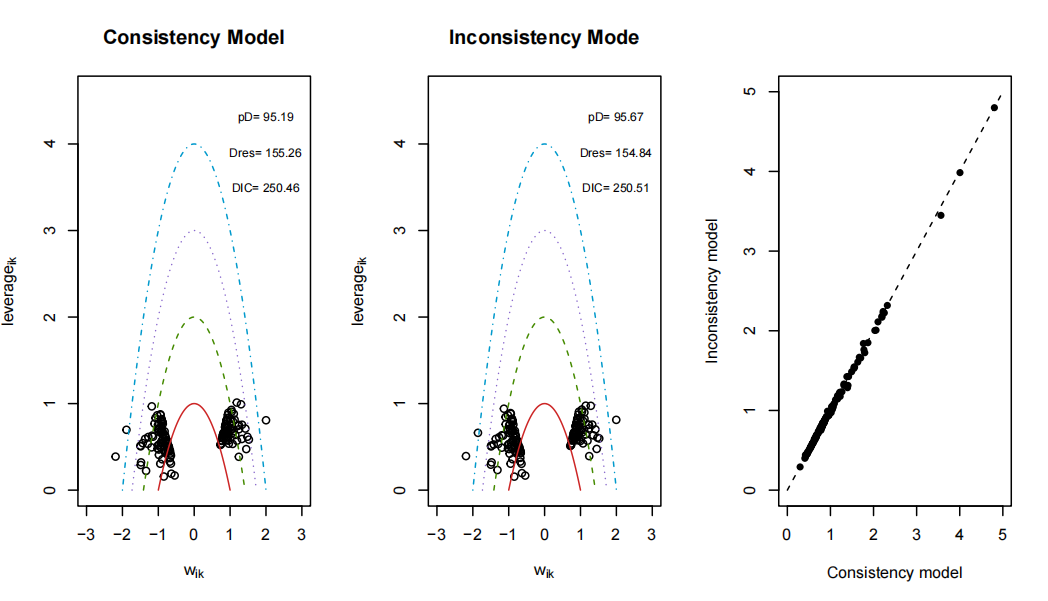


1. **Length of ICU Stay**


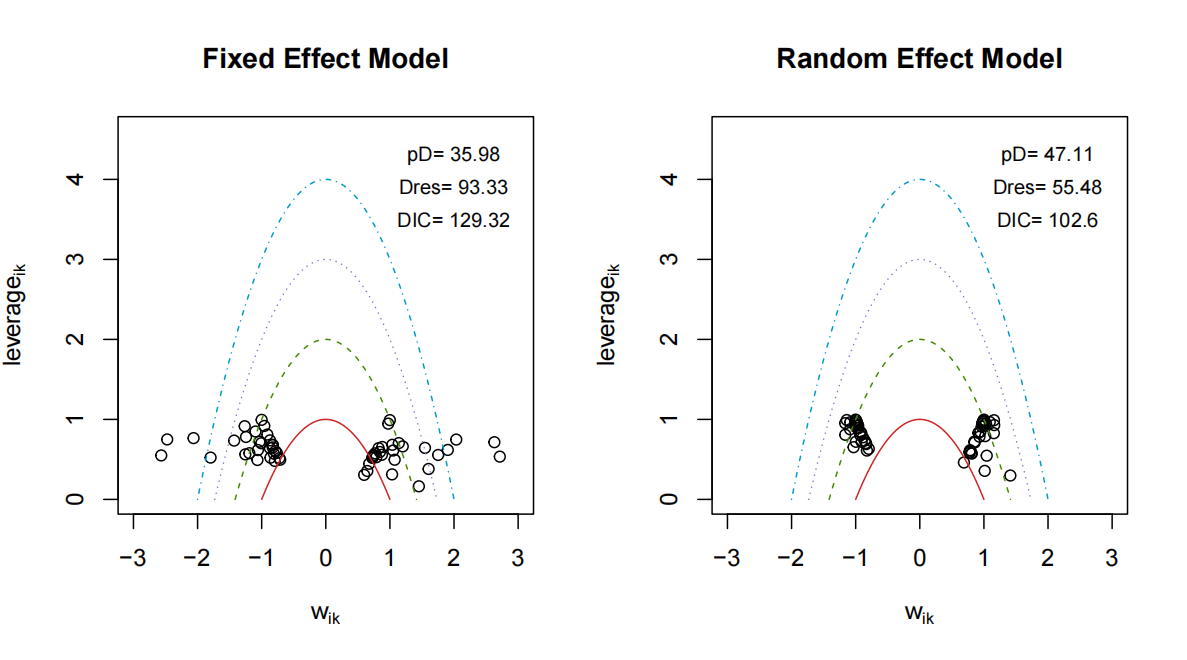


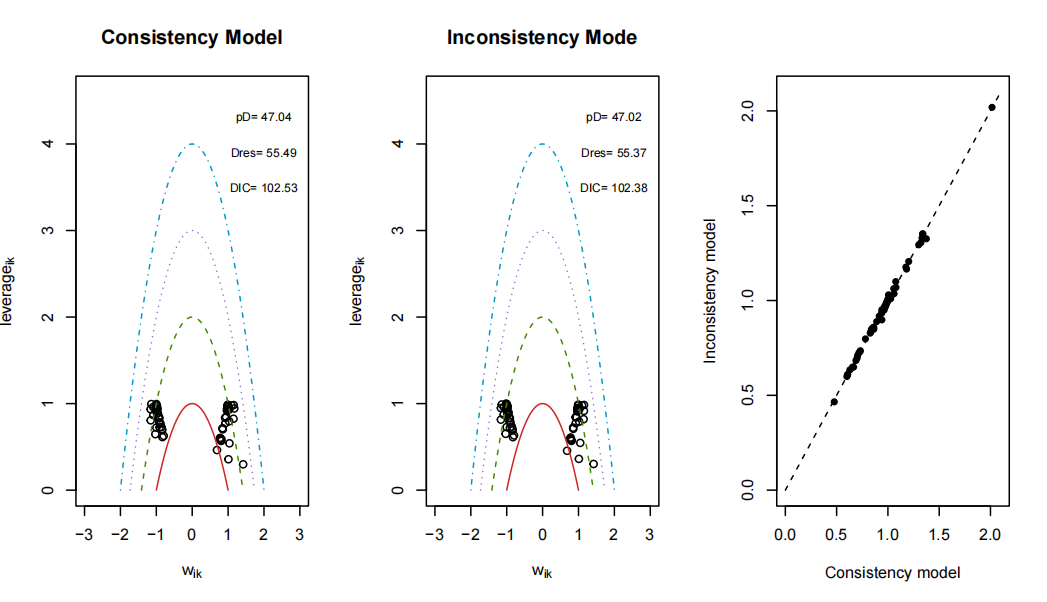


1. **Length of Hospital Stay**


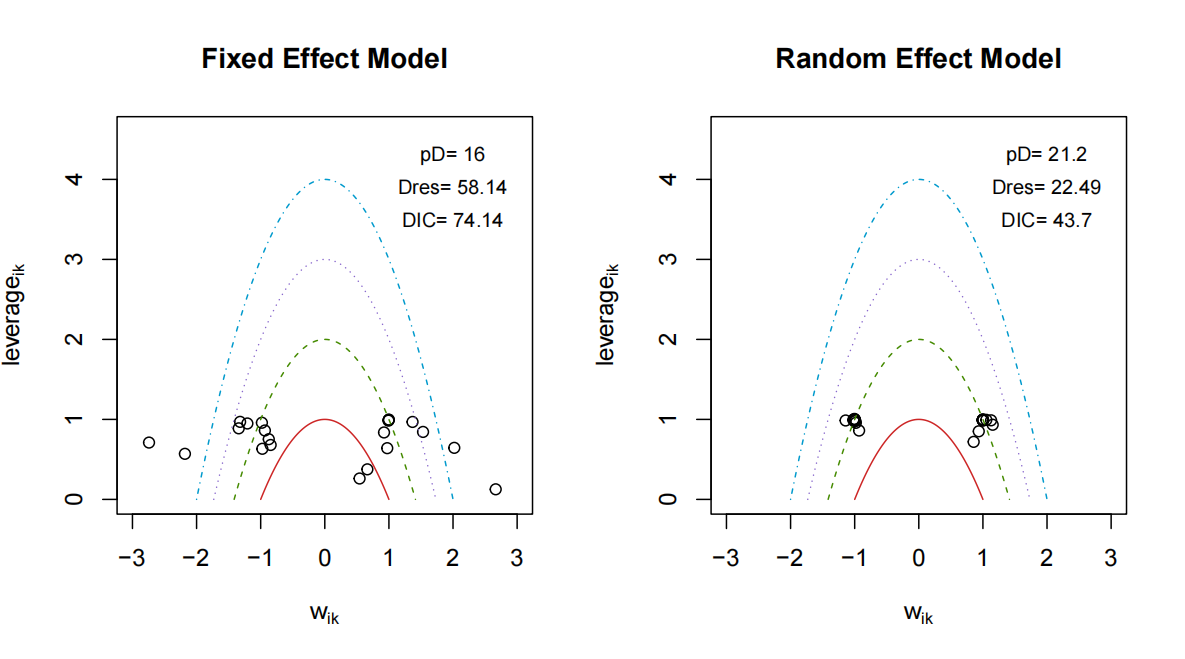


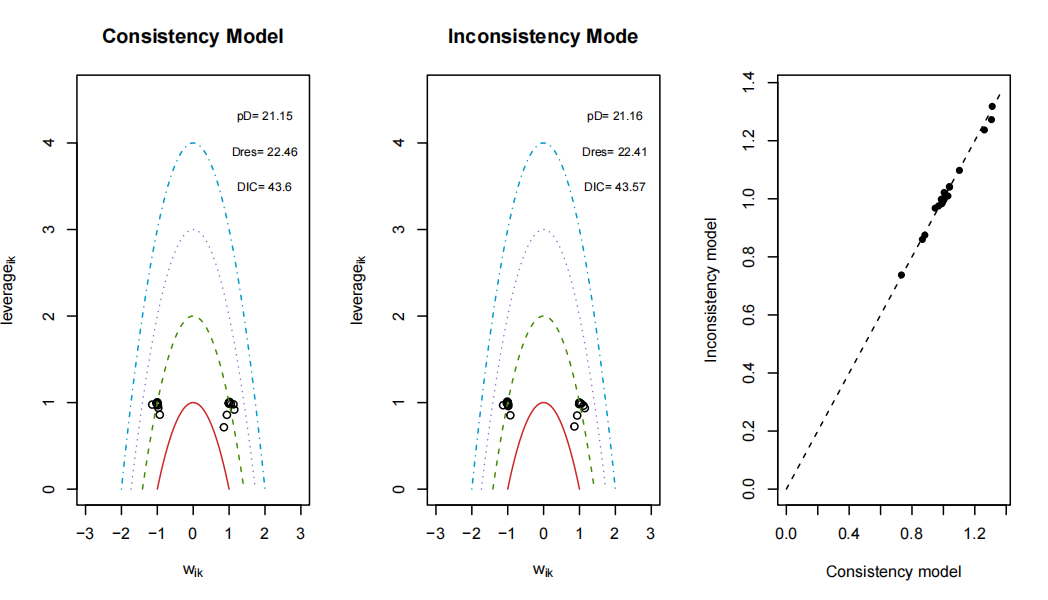


1. **Duration of Mechanical Ventilation**


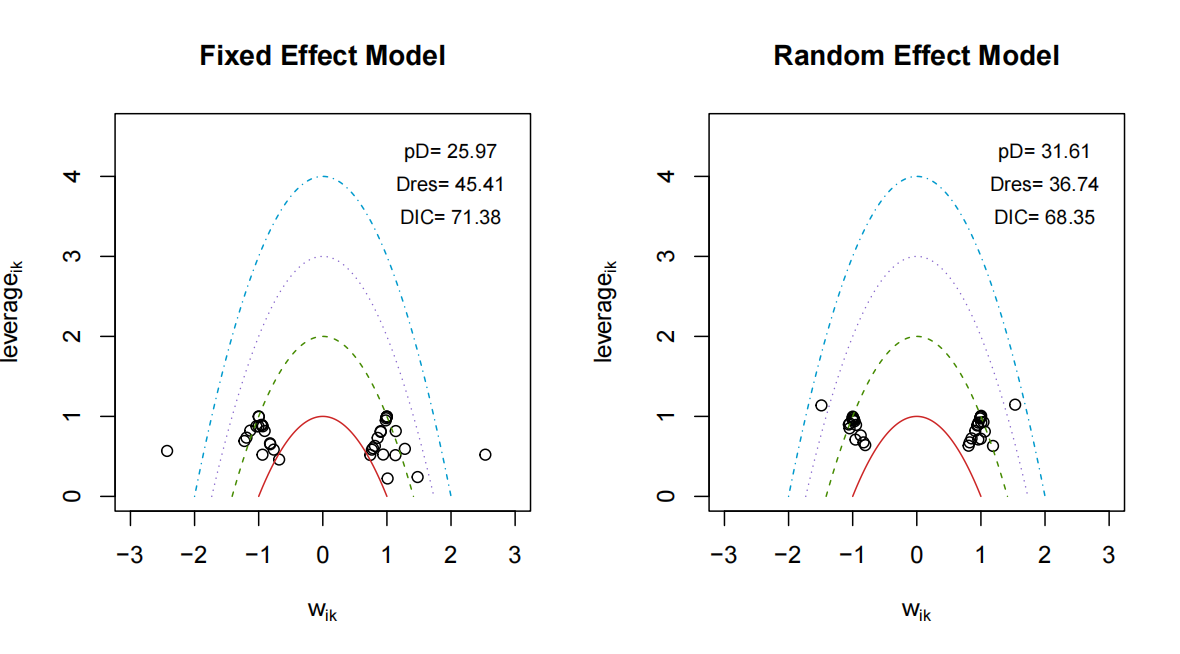


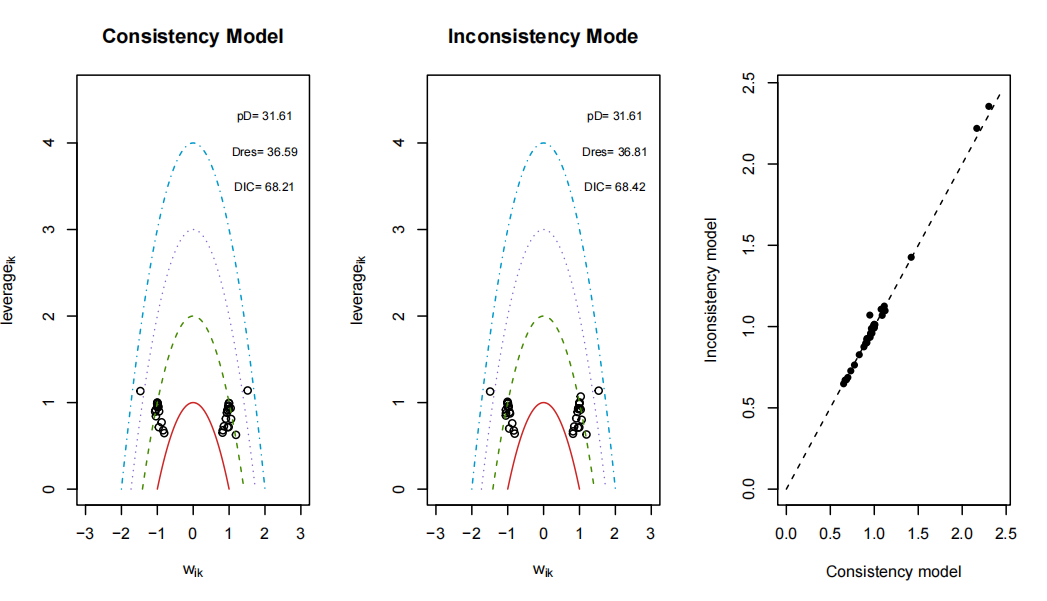


1. **Adverse Event**


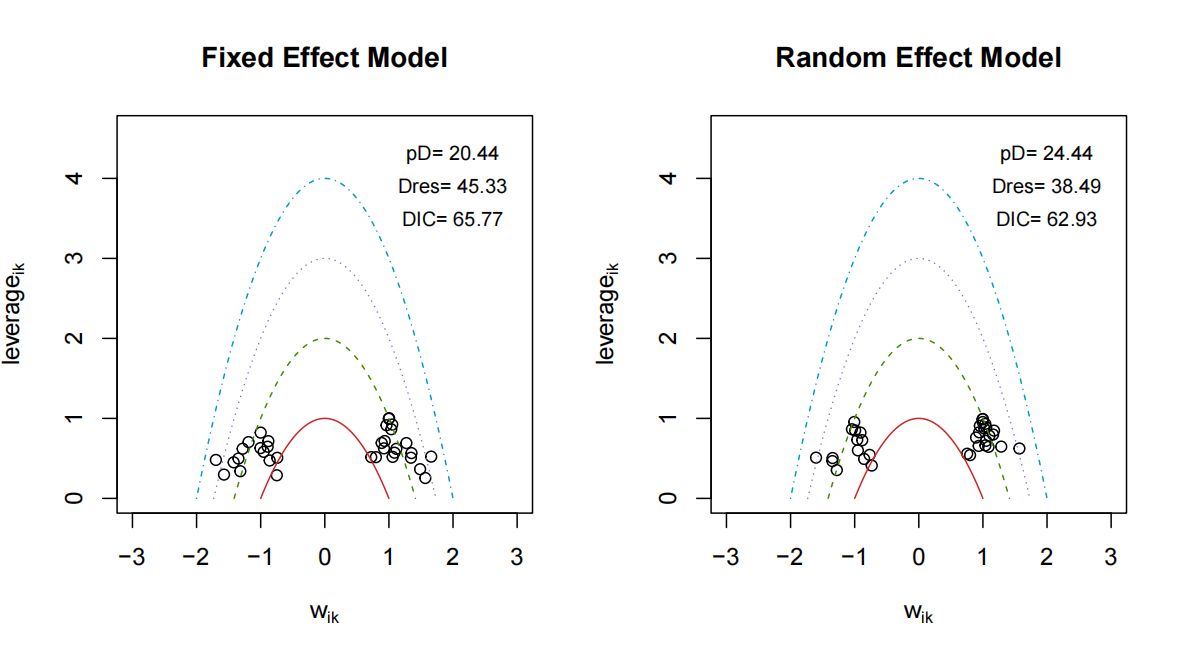


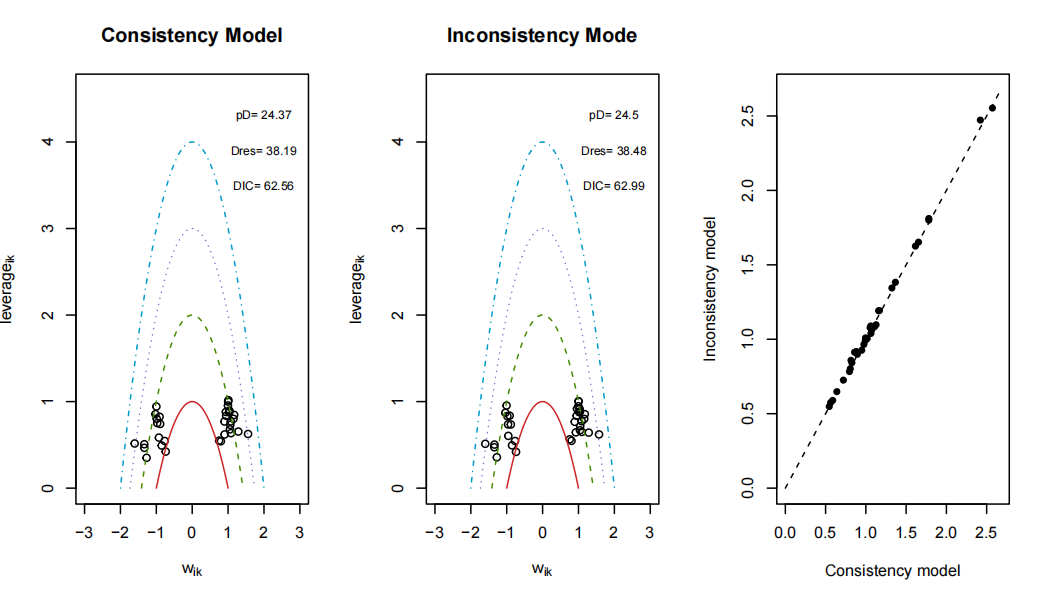


1. **Serious Adverse Event**


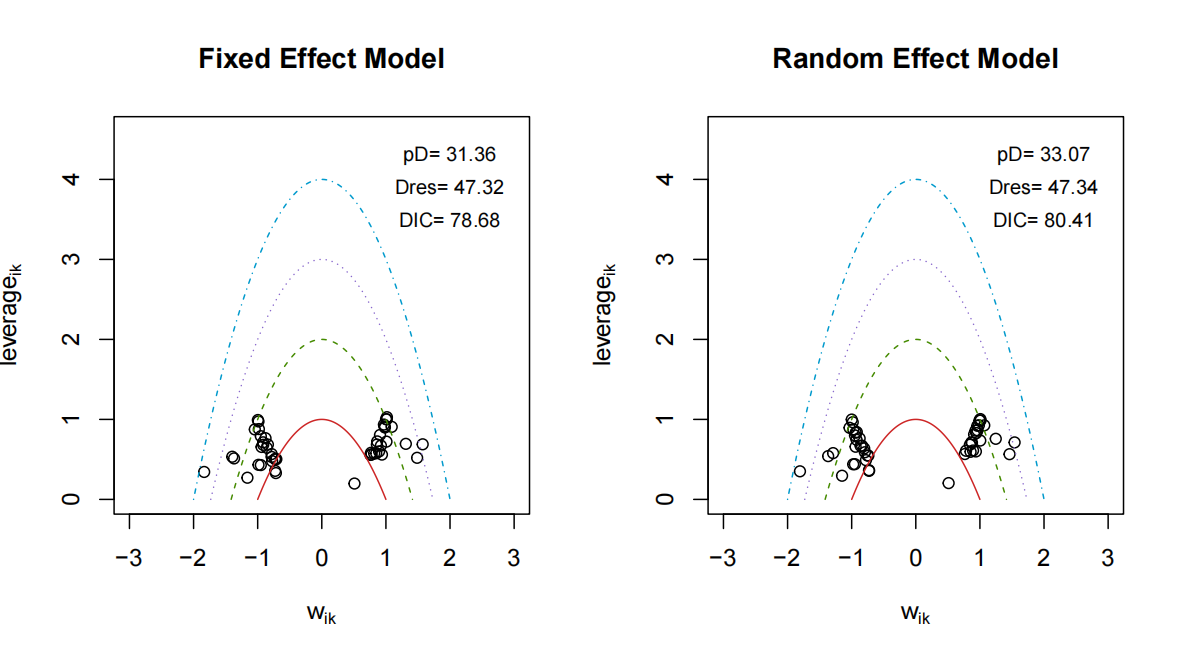


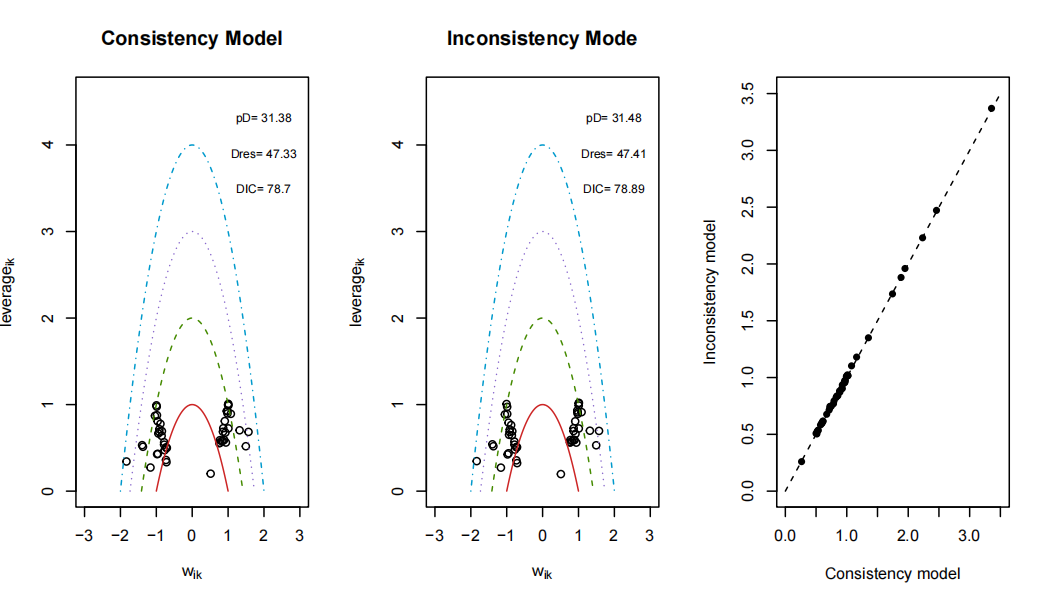


**Appendix 8: Funnel plot asymmetry assessments**

1. **All-cause Mortality**

**
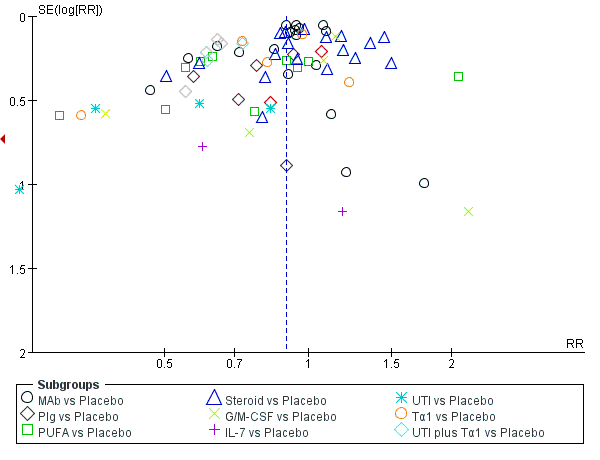
**

1. **Length of ICU Stay**

**
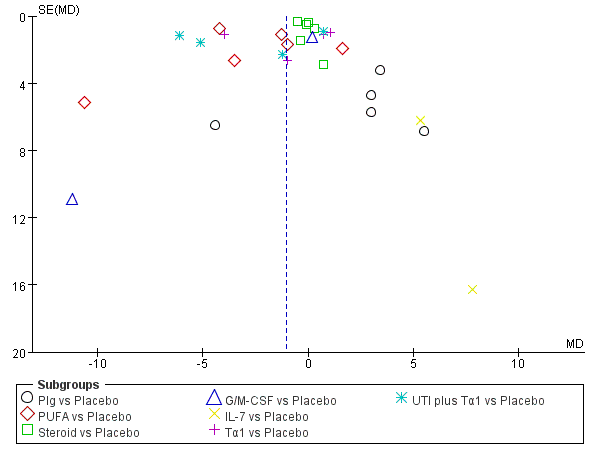
**

1. **Length of Hospital Stay**

**
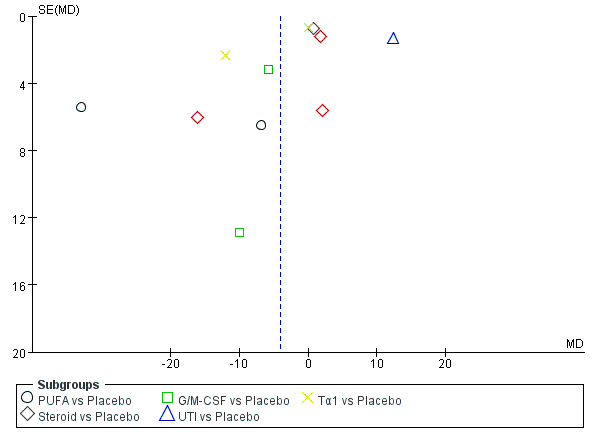
**

1. **Duration of Mechanical Ventilation**

**
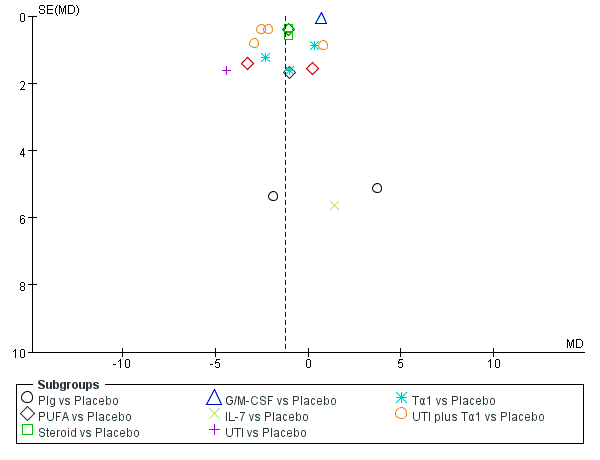
**

1. **Adverse Event**

**
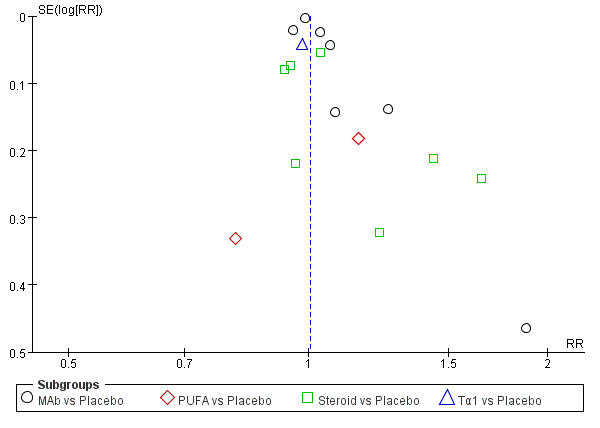
**

1. **Serious Adverse Event**

**
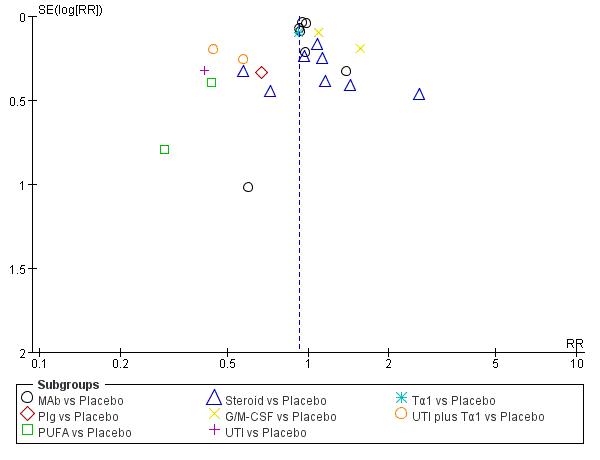
**

**Appendix 9: The rank probability and SUCRA plot for each treatment based on outcomes**

1. **Length of ICU Stay**


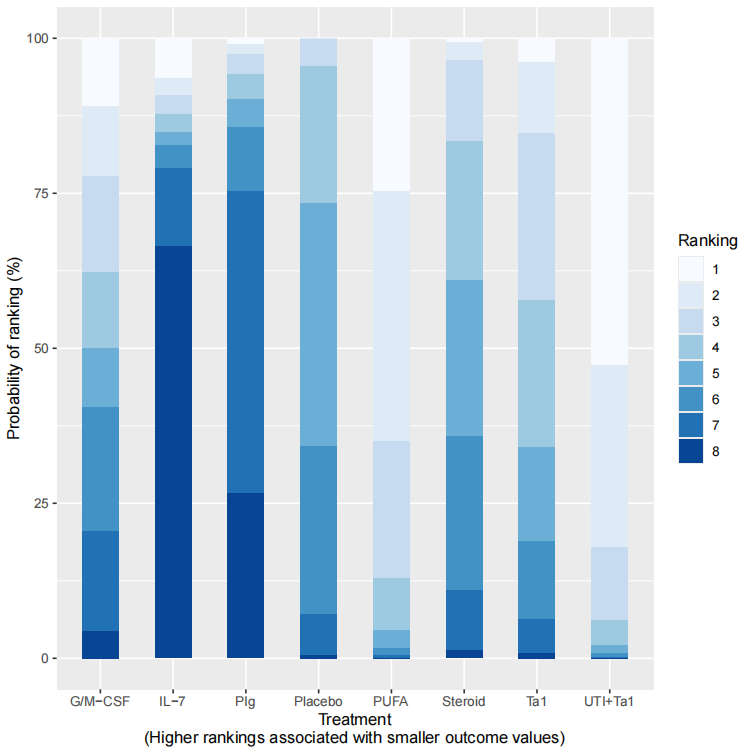

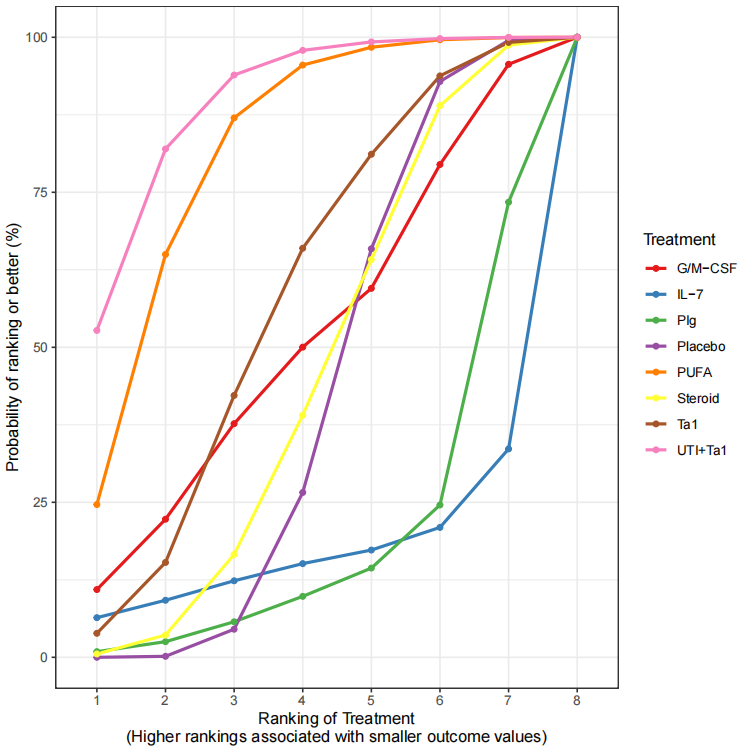


1. **Length of Hospital Stay**


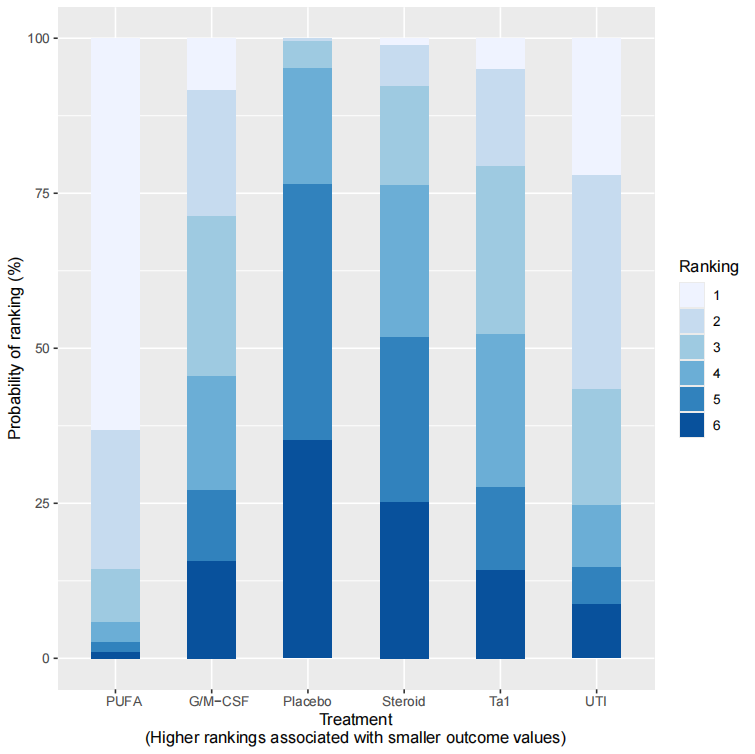

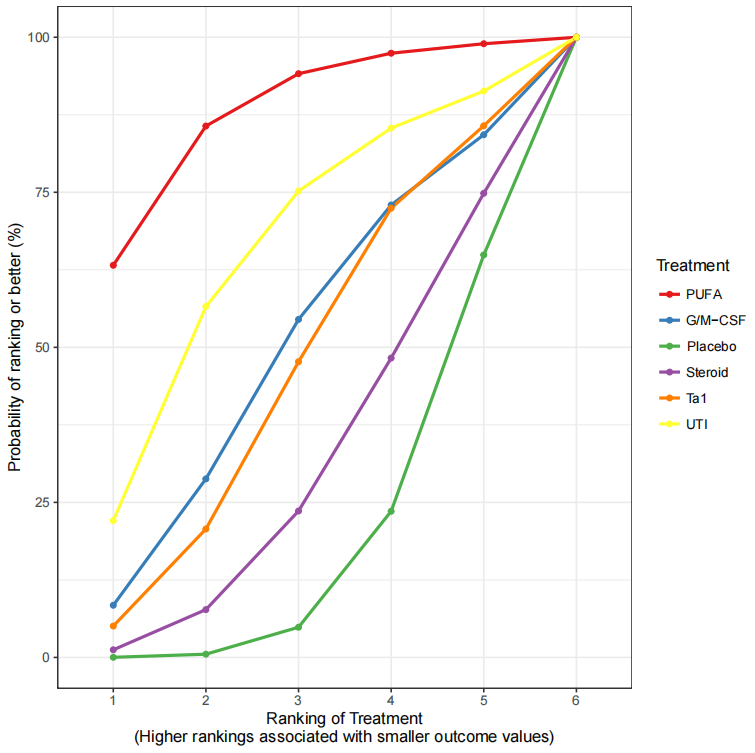


1. **Duration of Mechanical Ventilation**


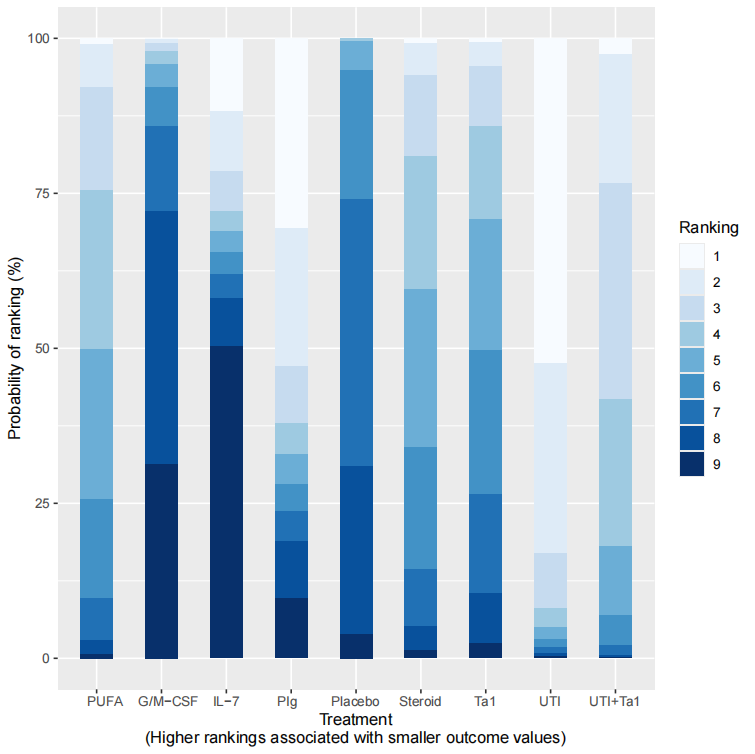

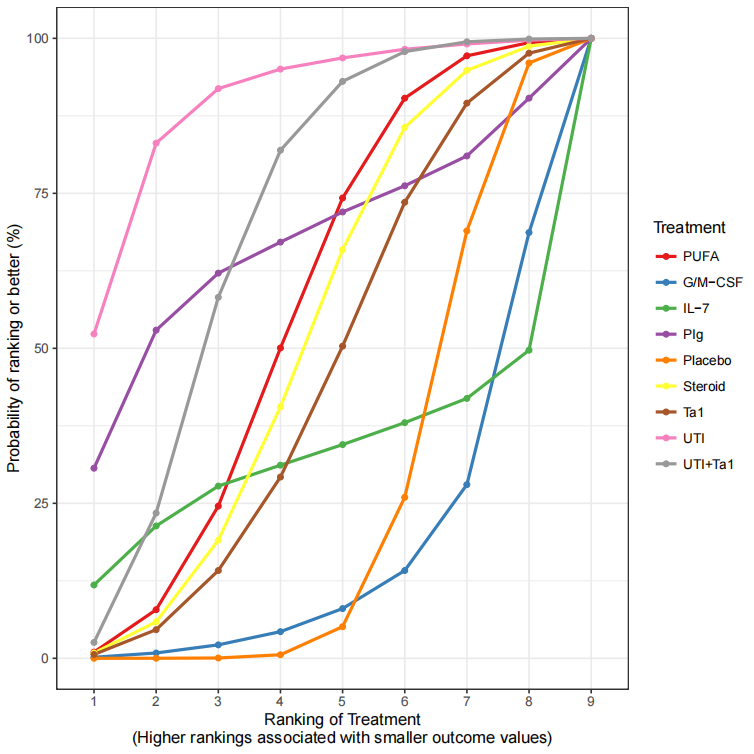


1. **Adverse Event**


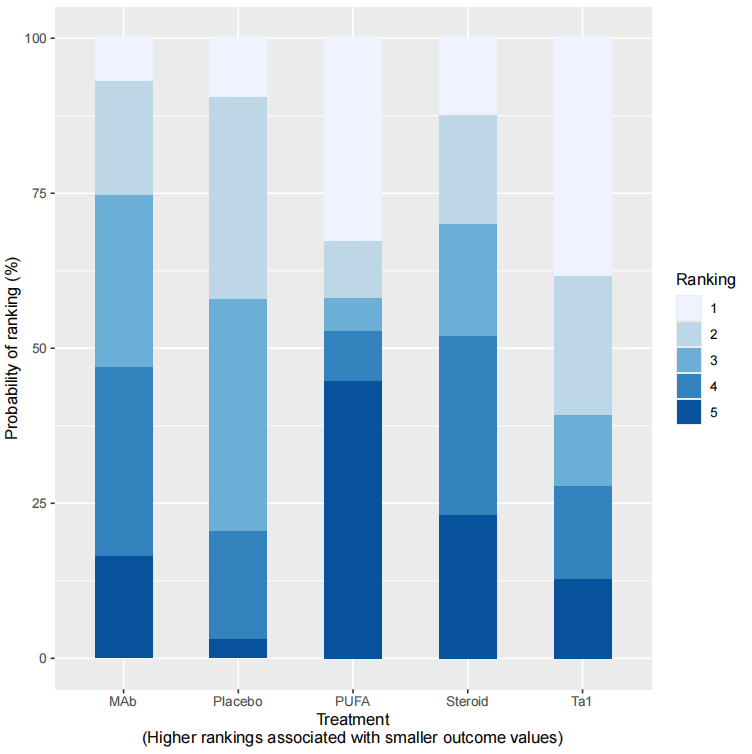

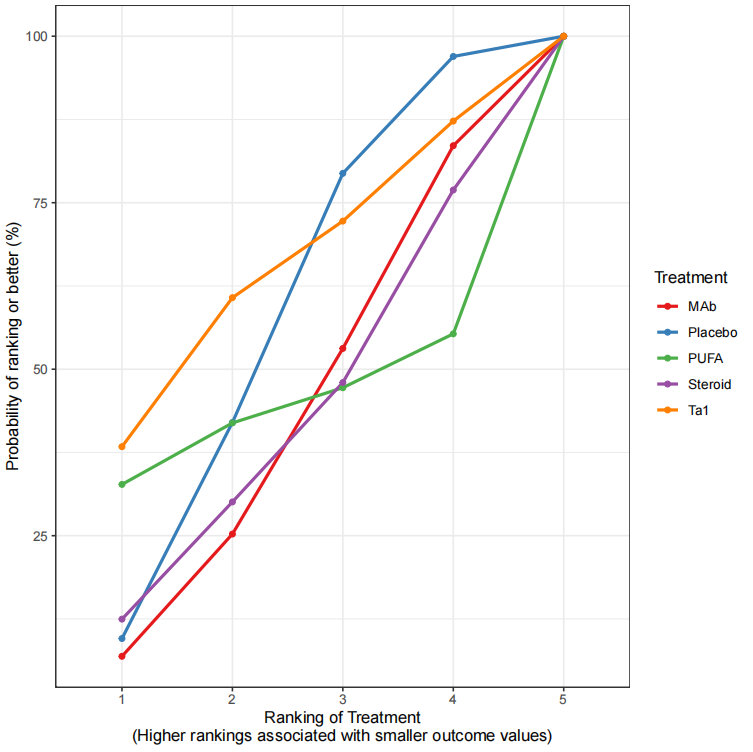


1. **Serious Adverse Event**


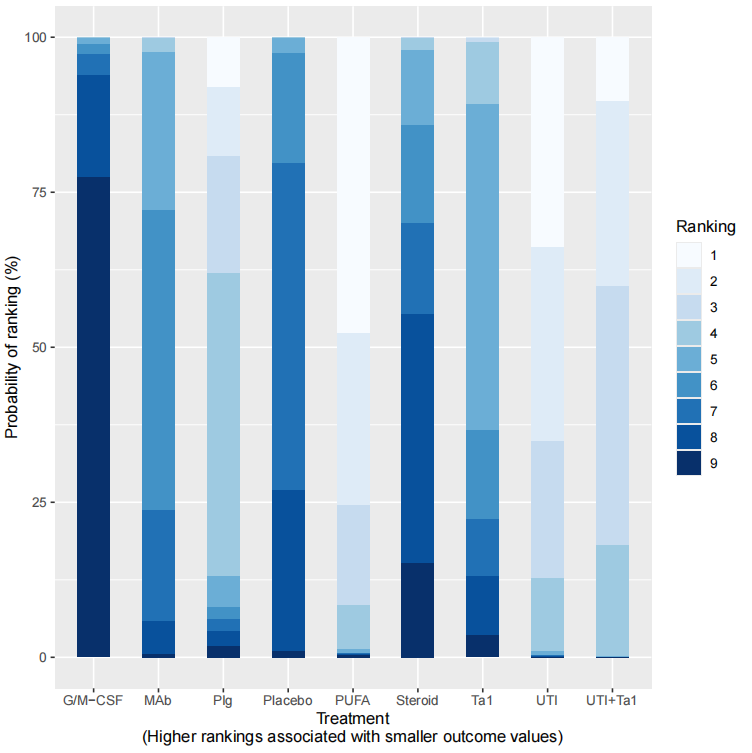

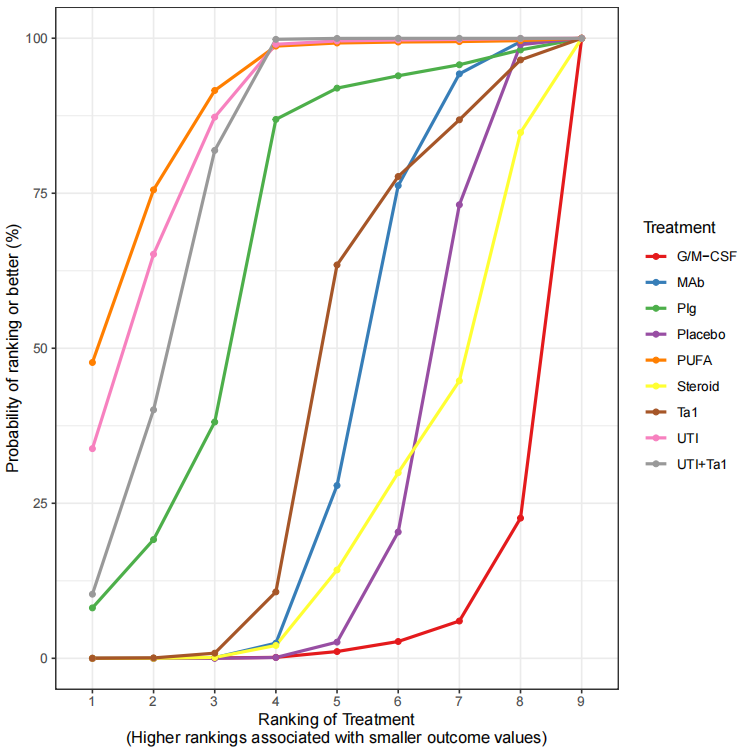


**Appendix 10: League heat table of pairwise RRs or MDs with 95% Crl**

1. **Length of ICU Stay（MD）**


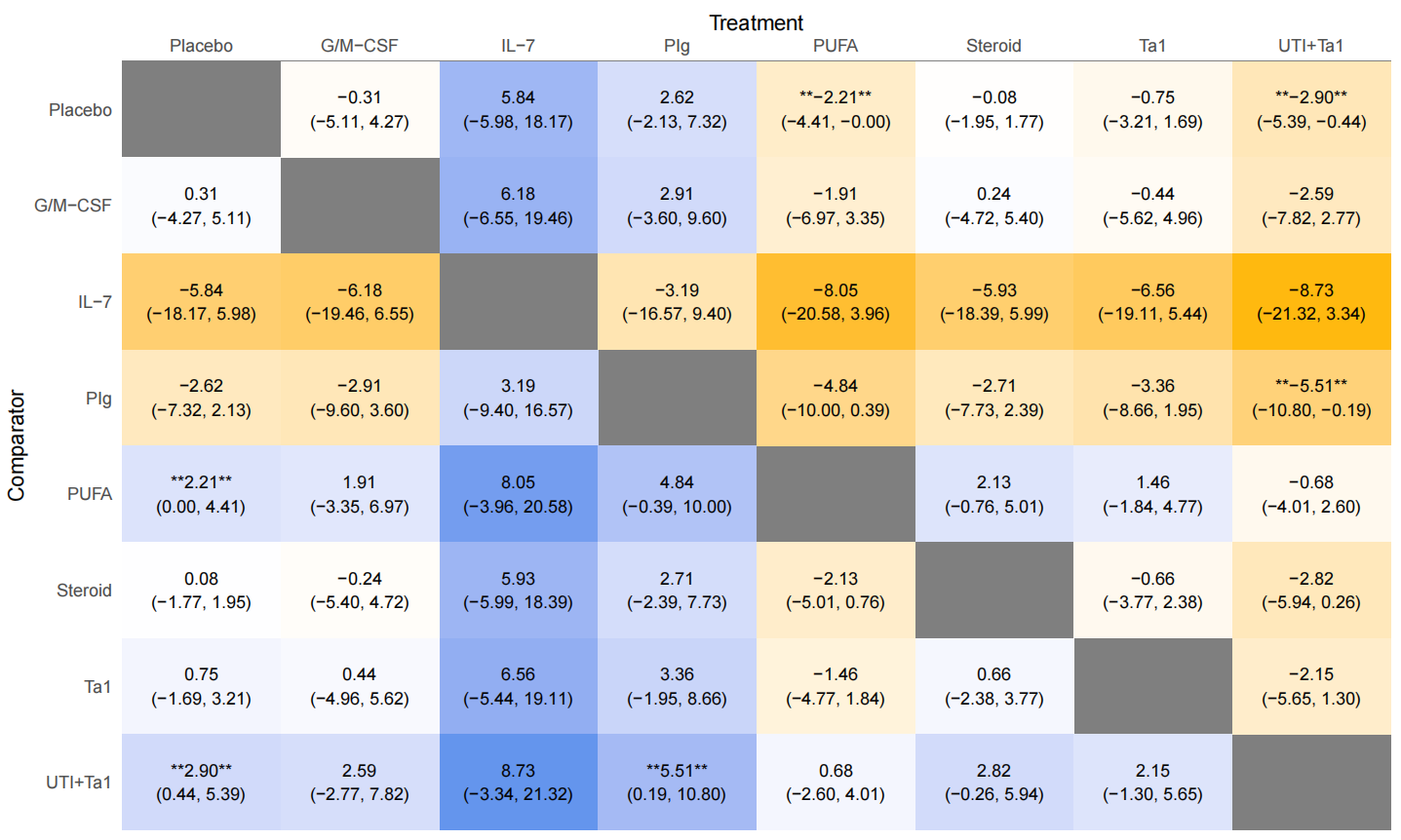


1. **Length of Hospital Stay（MD）**


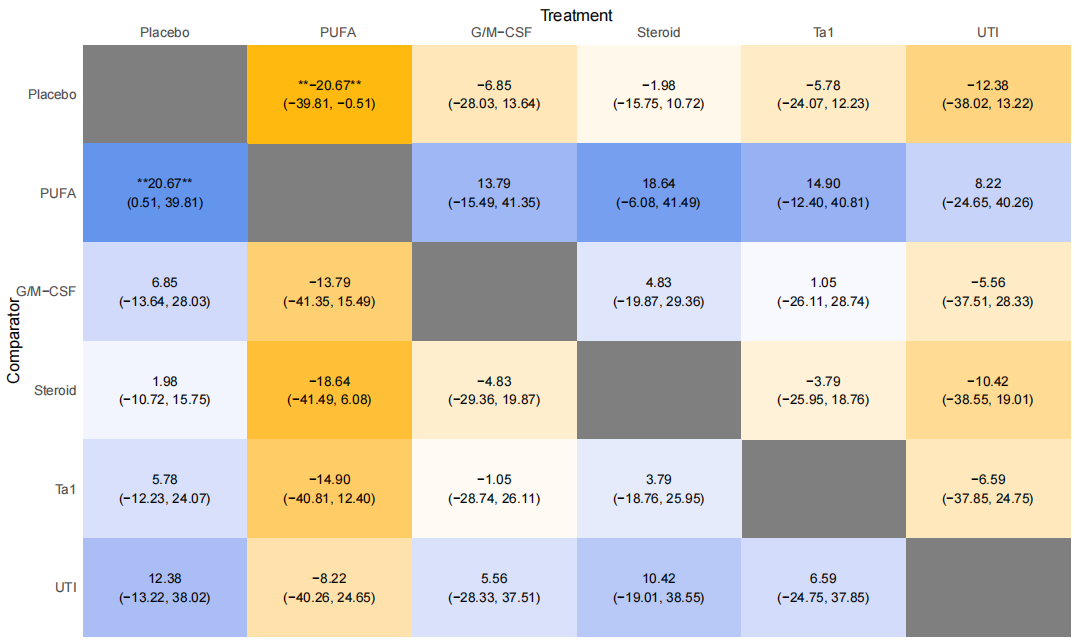


1. **Duration of Mechanical Ventilation（MD）**


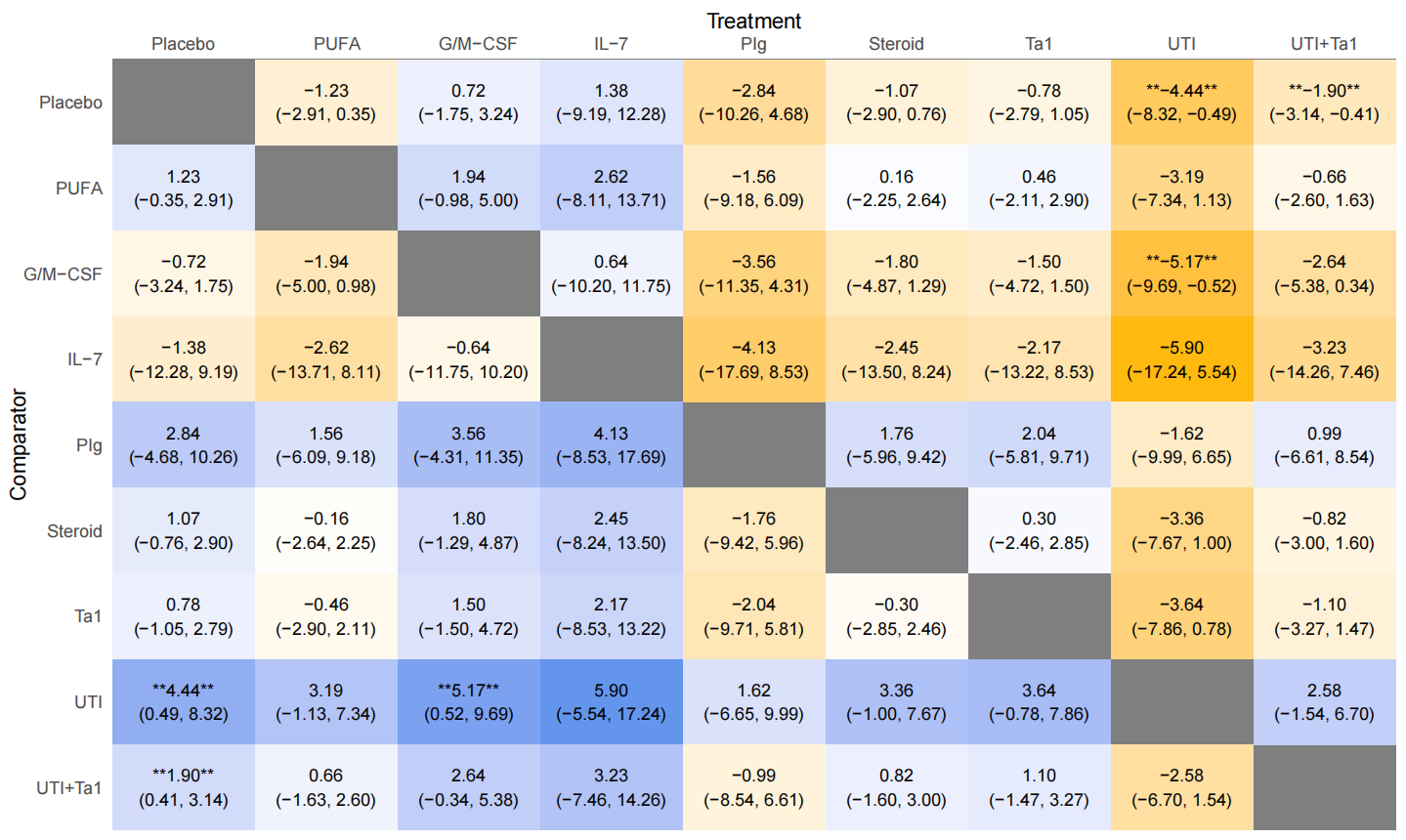


1. **Adverse Event（RR）**


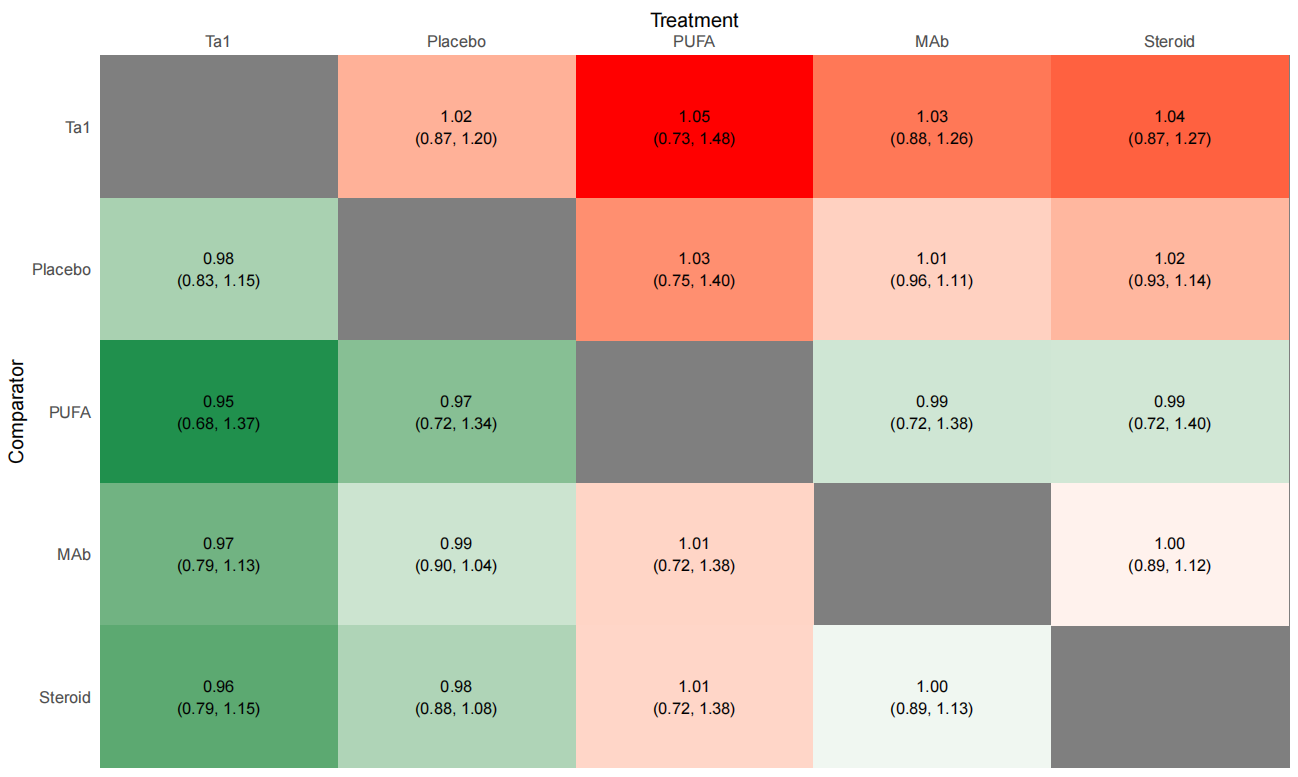


1. **Serious Adverse Event（RR）**


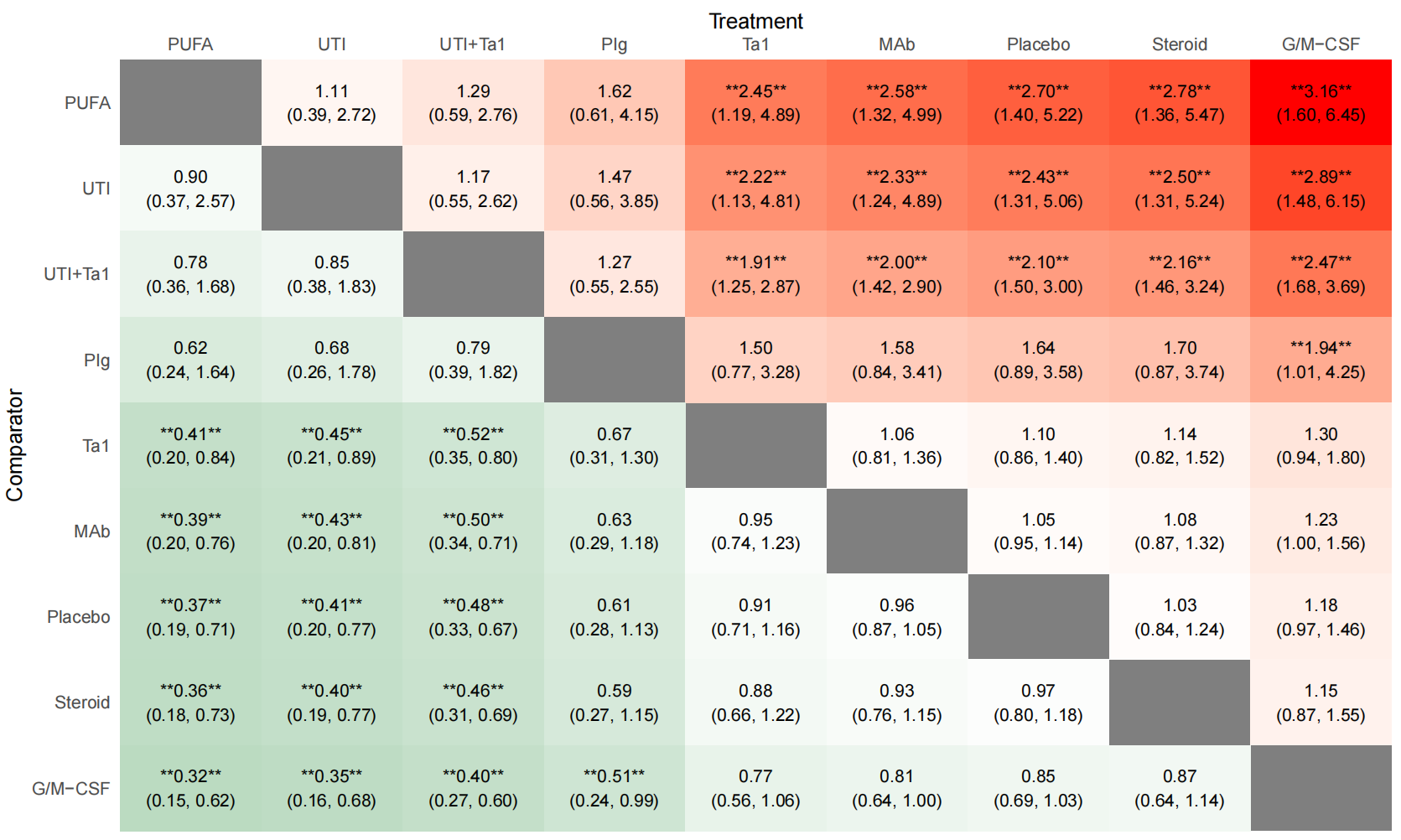


**Appendix 11: Results of the direct pairwise comparisons and GRADE assessments for all outcomes**

1. **Length of ICU Stay**

| Total studies:29 Total partici- pant:56,33 | Number of parti- cipants(trials) | risk of bias | inconsistency | indirectness | imprecision | Certainty of the evidence |
| --- | --- | --- | --- | --- | --- | --- |
| PIg vs Placebo | Direct evidence: 188（5 trials） | serious | not serious | not serious | very serious | ⨁◯ ◯ ◯ Very low |
| PUFA vs Placebo | Direct evidence: 492（6 trials） | very serious | serious | not serious | serious | ⨁◯ ◯ ◯ Very low |
| Steroid vs Placebo | Direct evidence: 2352（6 trials） | not serious | not serious | not serious | serious | ⨁⨁⨁◯ Moderate |
| G/M-CSF vs Placebo | Direct evidence: 202（2 trials） | not serious | not serious | not serious | very serious | ⨁⨁◯ ◯ Low |
| IL-7 vs Placebo | Direct evidence: 48（2 trials） | not serious | not serious | not serious | extremely serious | ⨁◯ ◯ ◯ Very low |
| Tα1 vs Placebo | Direct evidence: 1539（4 trials） | very serious | serious | not serious | serious | ⨁◯ ◯ ◯ Very low |
| UTI plus Tα1 vs  Placebo | Direct evidence: 812（4 trials） | serious | very serious | not serious | serious | ⨁◯ ◯ ◯ Very low |

1. **Length of Hospital Stay**

| Total studies:11 Total partici- pant:37,20 | Number of parti- cipants(trials) | risk of bias | inconsistency | indirectness | imprecision | Certainty of the evidence |
| --- | --- | --- | --- | --- | --- | --- |
| PUFA vs Placebo | Direct evidence: 83（2 trials） | very serious | very serious | not serious | extremely serious | ⨁◯ ◯ ◯ Very low |
| Steroid vs Placebo | Direct evidence: 2190（4 trials） | not serious | serious | not serious | serious | ⨁⨁◯ ◯ Low |
| G/M-CSF vs Placebo | Direct evidence: 202（2 trials） | not serious | not serious | not serious | serious | ⨁⨁⨁◯ Moderate |
| UTI vs Placebo | Direct evidence: 114（1 trials） | not serious | not serious | serious | serious | ⨁⨁◯ ◯ Low |
| Tα1 vs Placebo | Direct evidence: 1131（2 trials） | serious | very serious | not serious | extremely serious | ⨁◯ ◯ ◯ Very low |

1. **Duration of Mechanical Ventilation**

| Total studies:18 Total partici- pant:21,83 | Number of parti- cipants(trials) | risk of bias | inconsistency | indirectness | imprecision | Certainty of the evidence |
| --- | --- | --- | --- | --- | --- | --- |
| PIg vs Placebo | Direct evidence: 75（2 trials） | very serious | not serious | not serious | extremely serious | ⨁◯ ◯ ◯ Very low |
| PUFA vs Placebo | Direct evidence: 384（4 trials） | serious | not serious | not serious | serious | ⨁⨁◯ ◯ Low |
| Steroid vs Placebo | Direct evidence: 163（2 trials） | not serious | not serious | not serious | serious | ⨁⨁⨁◯ Moderate |
| G/M-CSF vs Placebo | Direct evidence: 164（1 trials） | not serious | not serious | serious | serious | ⨁⨁◯ ◯ Low |
| IL-7 vs Placebo | Direct evidence: 21（1 trials） | not serious | not serious | serious | extremely serious | ⨁◯ ◯ ◯ Very low |
| UTI vs Placebo | Direct evidence: 114（1 trials） | not serious | not serious | serious | serious | ⨁⨁◯ ◯ Low |
| Tα1 vs Placebo | Direct evidence: 450（3 trials） | very serious | not serious | not serious | serious | ⨁◯ ◯ ◯ Very low |
| UTI plus Tα1 vs  Placebo | Direct evidence: 812（4 trials） | very serious | serious | not serious | not serious | ⨁◯ ◯ ◯ Very low |

1. **Adverse Event**

| Total studies:17 Total partici- pant:11,002 | Number of parti- cipants(trials) | risk of bias | inconsistency | indirectness | imprecision | Certainty of the evidence |
| --- | --- | --- | --- | --- | --- | --- |
| MAb vs Placebo | Direct evidence: 8,158（7 trials） | serious | not serious | not serious | serious | ⨁⨁◯ ◯ Low |
| PUFA vs Placebo | Direct evidence: 279（2 trials） | serious | not serious | not serious | serious | ⨁⨁◯ ◯ Low |
| Steroid vs Placebo | Direct evidence: 1,476（7 trials） | serious | not serious | not serious | serious | ⨁⨁◯ ◯ Low |
| Tα1 vs Placebo | Direct evidence: 1,089（1 trials） | not serious | not serious | not serious | serious | ⨁⨁⨁◯ Moderate |

1. **Serious Adverse Event**

| Total studies:24 Total partici- pant:1,3843 | Number of parti- cipants(trials) | risk of bias | inconsistency | indirectness | imprecision | Certainty of the evidence |
| --- | --- | --- | --- | --- | --- | --- |
| MAb vs Placebo | Direct evidence: 8,681（7 trials） | serious | not serious | not serious | serious | ⨁⨁◯ ◯ Low |
| PIg vs Placebo | Direct evidence: 42（1 trials） | very serious | not serious | serious | very serious | ⨁◯ ◯ ◯ Very low |
| PUFA vs Placebo | Direct evidence: 279（2 trials） | serious | not serious | not serious | not serious | ⨁⨁⨁◯ Moderate |
| Steroid vs Placebo | Direct evidence: 2,417（8 trials） | serious | not serious | not serious | serious | ⨁⨁◯ ◯ Low |
| G/M-CSF vs Placebo | Direct evidence: 865（2 trials） | serious | serious | not serious | serious | ⨁◯ ◯ ◯ Very low |
| UTI vs Placebo | Direct evidence: 114（1 trials） | not serious | not serious | serious | not serious | ⨁⨁⨁◯ Moderate |
| Tα1 vs Placebo | Direct evidence: 1,089（1 trials） | not serious | not serious | not serious | serious | ⨁⨁⨁◯ Moderate |
| UTI plus Tα1 vs  Placebo | Direct evidence: 356（2 trials） | serious | not serious | not serious | not serious | ⨁⨁⨁◯ Moderate |
